# Refinement of rice blast disease resistance QTLs and gene networks through meta-QTL analysis

**DOI:** 10.1038/s41598-024-64142-0

**Published:** 2024-07-16

**Authors:** Basavantraya Navadagi Devanna, Sumali Sucharita, N. C. Sunitha, C. Anilkumar, Pankaj K. Singh, D. Pramesh, Sanghamitra Samantaray, Lambodar Behera, Jawahar Lal Katara, C. Parameswaran, Prachitara Rout, Selvaraj Sabarinathan, Hosahatti Rajashekara, Tilak Raj Sharma

**Affiliations:** 1 ICAR-National Rice Research Institute, Cuttack, Odisha 753006 India; 2 Department of Biotechnology, University Centre for Research and Development, Chandigarh University, Mohali, Punjab 140413 India; 3 University of Agricultural Sciences, Raichur, Karnataka India; 4 ICAR-Directorate of Cashew Research, Puttur, Karnataka 574202 India; 5 Division of Crop Science, Indian Council of Agricultural Research, Krishi Bhavan, New Delhi, 110001 India

**Keywords:** Candidate genes, Characterized genes, *Magnaporthe oryzae*, Meta-QTL, Orthologous genes, Rice blast, Resistance gene analogs, Biotechnology, Genetics, Microbiology, Plant sciences

## Abstract

Rice blast disease is the most devastating disease constraining crop productivity. Vertical resistance to blast disease is widely studied despite its instability. Clusters of genes or QTLs conferring blast resistance that offer durable horizontal resistance are important in resistance breeding. In this study, we aimed to refine the reported QTLs and identify stable meta-QTLs (MQTLs) associated with rice blast resistance. A total of 435 QTLs were used to project 71 MQTLs across all the rice chromosomes. As many as 199 putative rice blast resistance genes were identified within 53 MQTL regions. The genes included 48 characterized resistance gene analogs and related proteins, such as NBS–LRR type, LRR receptor-like kinase, NB-ARC domain, pathogenesis-related TF/ERF domain, elicitor-induced defense and proteins involved in defense signaling. MQTL regions with clusters of RGA were also identified. Fifteen highly significant MQTLs included 29 candidate genes and genes characterized for blast resistance, such as *Piz*, *Nbs-Pi9*, *pi55-1, pi55-2, Pi3/Pi5-1, Pi3/Pi5-2, Pikh, Pi54, Pik/Pikm/Pikp*, *Pb1* and *Pb2*. Furthermore, the candidate genes (42) were associated with differential expression (in silico) in compatible and incompatible reactions upon disease infection. Moreover, nearly half of the genes within the MQTL regions were orthologous to those in *O. sativa* indica, *Z. mays* and *A. thaliana,* which confirmed their significance. The peak markers within three significant MQTLs differentiated blast-resistant and susceptible lines and serve as potential surrogates for the selection of blast-resistant lines. These MQTLs are potential candidates for durable and broad-spectrum rice blast resistance and could be utilized in blast resistance breeding.

## Introduction

Rice is one of the major staple crops, feeding more than 30% of the global population. Expected rice production is threatened by an increased incidence of biotic and abiotic stresses hampering food security. Rice blast disease caused by *Pyricularia oryzae* Cavara (teleomorph: *Magnaporthe oryzae* B. Couch) is a destructive fungal disease that causes losses of 10–100%^1^. It causes infection at all stages of the crop cycle, with an incidence in all agro-ecologies in both temperate and tropical environments. It is typically designated as leaf blast (leaves), collar blast (collar), neck blast (culm, culm node, panicle neck nodes), or panicle blast (panicles) based on the rice tissue part showing infection and symptoms. However, it is also known to affect roots^2^. *Magnaporthe* is an ascomycete fungus that causes airborne disease to spread through conidia and is more predominant under high humidity^3^. It is globally regarded as the most notorious disease due to its distribution across 85 countries^4^.

The enormous diversity among the pathogenic *M. oryzae* races has a negative impact on its control and management. Cultural, chemical, and biological strategies have been developed and deployed to control this disease. Biological control, such as the use of biocontrol agents^5,6^ and deployment of resistant cultivars, is an eco-friendly approach. However, wide application, effectiveness, and durability can be achieved only through the identification of stable resistant sources and their utilization in crop breeding. Moreover, molecular markers have been used effectively to reduce screening and increase the efficiency of transfer of resistance in terms of pace and precision. Over a hundred blast resistance genes have been reported across all the rice chromosomes^1,7^. Most of these treatments are race specific and have limited durability. However, blast resistance seldom shows quantitative inheritance encompassing a cluster of genes^1^. Riveting on race-nonspecific and broad-spectrum genes helps achieve durable horizontal resistance^8^. In this context, the discovery and characterization of quantitative trait loci (QTLs) governing rice blast resistance are of paramount interest.

Several QTLs linked to blast resistance in rice have been reported from different environments using different mapping populations^9–12^. In addition, most of them (except for a few QTLs with major effects) are restrained from deployment in marker assisted selection (MAS), speculating uncertainty. The uncertainty or instability of QTLs is associated with different genetic backgrounds and growing environments^13^. QTL effects are influenced by epistasis and modifier effects, both of which are more sensitive to genetic background^14^. Furthermore, biased QTL-environment correlations will restrict the use of a QTL across different environments. Limited recombination in biparental mapping populations causes several problems, such as low population size, low logarithm of odds (LOD) score, genotyping errors, and phenotyping in minimal environments, which reduce the effectiveness of QTLs in MAS^15^. Despite these limitations, QTL mapping is a modest way to understand the genetics underlying the trait of interest^16^. Hence, identifying a robust consensus genomic region using QTL information from independent studies has found relevance. With the availability of vast rice genomic resources and databases, cataloging and compiling QTL information on a consensus map is feasible. Meta-QTL analysis is instrumental in integrating QTL information from independent studies to identify and project robust QTLs on a consensus map^16–18^. This approach aims to discover whether the QTL for a trait or related traits from different studies colocalize on the consensus map. Furthermore, this method identifies ‘real QTL’ with refined QTL positions and narrows its confidence interval (CI)^19^. A narrowed CI of a QTL decreases the number of genes predicted within the interval by more than that of the original QTL, thereby increasing the recovery of probable candidates for the trait^17^.

With this perspective, we framed the study by hypothesizing the presence of stable large-effect QTLs associated with rice blast resistance in the rice genome. Meta-QTL analysis was implemented to test this hypothesis with the following objectives: (a) projection of MQTLs conferring rice blast resistance considering the original QTL; (b) identification of peak (nearest) markers linked to MQTLs; and (c) mining of the candidate genes underlying the MQTL region and predicting their role in the rice blast resistance.

## Results

### Discovery of MQTLs for rice blast resistance

**Figure 1:**
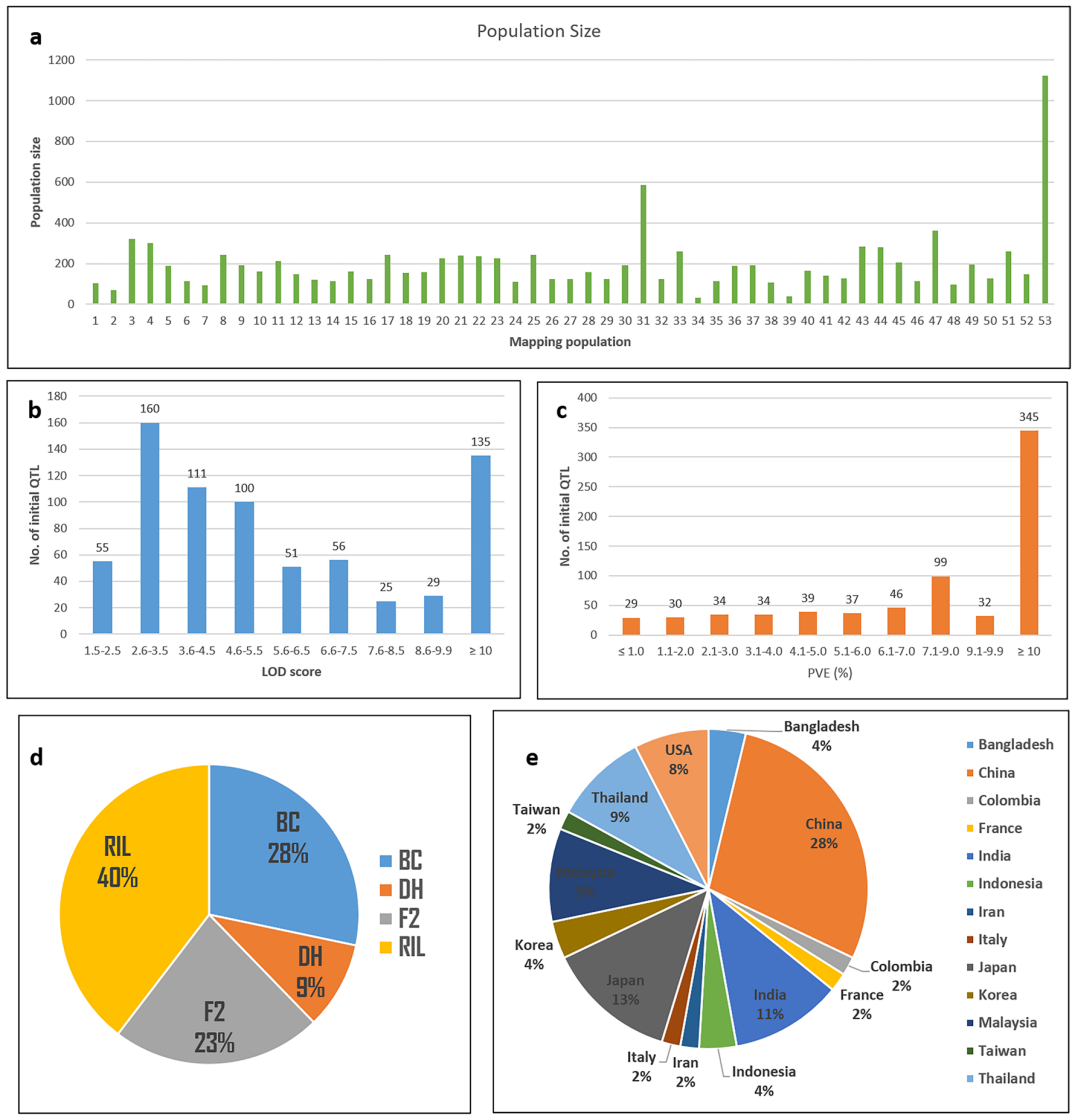
Frequency distribution of (**a**) population size of each of the mapping populations used, (**b**) LOD score of the original QTLs and (**c**) phenotypic variation (%) explained by the original QTL. Graphical representation of the proportions of independent studies (**d**) using different biparental mapping populations and (**d**) countries from where the study is reported.

**Figure 2:**
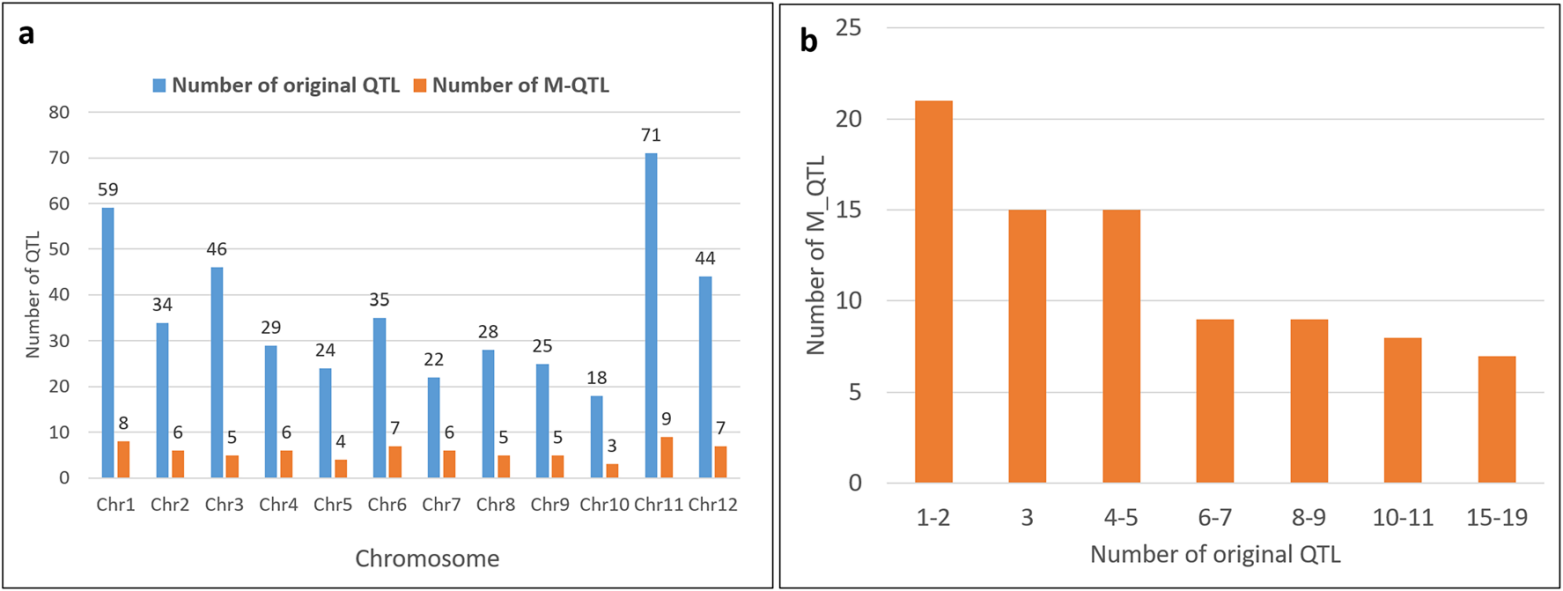
Depiction of the decrease in the number of original QTLs through MQTL analysis as represented in the chromosome-wise distribution (**a**) and (**b**) the number of original QTLs within MQTLs.

Information on a total of 737 QTLs associated with rice blast resistance was collected from 53 independent studies reported over the past two decades (Table 1). These studies included recombinant inbred line (RIL, 40%), backcross (BC, 28%), F_2_ (23%), and doubled haploid (DH, 9%) mapping populations (Fig. 1d) with sizes ranging from 31 to 1125 (Fig. 1a), which were evaluated for rice blast resistance across 14 different countries (Fig. 1e). Blast-resistant QTLs were reported across all the rice chromosomes, with the maximum occurring on chromosome 11 with 71 initial QTLs, and a minimum of 18 initial QTLs reported on chromosome 10 (Fig. 2a). The majority (682) of the initial QTLs were significant with a LOD > 2.5, except for a few (55) (Fig. 1b). The initial QTLs included 345 major-effect QTLs with a PVE ≥ 10%, while the remaining were minor-effect QTLs (Fig. 1c). A consensus map was generated using the individual maps from each of the 53 experiments.

**Table 1:** Details of the QTL mapping studies on rice blast resistance used for meta-QTL analysis.

Sl. No.	Parentage	Mapping population type	Population size	Experiment place	Experiment year	No. of QTL	References
1	Akhanaphou × Leimaphou	RIL	103	India	2017	2	^12^
2	Suweon_365 × Ki_313	F_2_	69	Korea	2000	2	^60^
3	Pongsuseribu2 × Mahsuri	F_2_	320	Malaysia	2011	28	^61^
4	Mahsuri × Pongsuseribu2	F_2_	300	Malaysia	2012	14	^62^
5	Mahsuri × Pongsuseribu2	F_2_	188	Malaysia	2013	13	^63^
6	IR64 × Azucena	DH	114	India	2000	12	^64^
7	IR64 × Azucena	DH	93	India	2007	13	^65^
8	Zhenshan × Minguhi_63	RIL	241	China	2003	9	^66^
9	Suweon365 × Chucheong	RIL	190	Korea	2008	8	^67^
10	Heikezijing × Suyunuo	RIL	162	China	2013	2	^68^
11	Bodao × Suyunuo	RIL	212	China	2014	2	^26^
12	Nipponbare × Owarihatamochi	F_2_	146	Japan	2001	4	^69^
13	Fanny × Ol5	RIL	120	Colombia	2006	59	^70^
14	Dongnong415 × Lijiangxintuanheigu	RIL	114	China	2016	10	^71^
15	Heikezijing × Suyunuo	RIL	162	China	2017	5	^72^
16	IR64 × Azucena	F2	125	India	2003	3	^73^
17	Minguhi63 × Zhenshan97	RIL	241	China	2008	4	^74^
18	Bodao × Suyunuo	BC	155	China	2014	52	^75^
19	Miyazakimochi × Bikei22	BC	158	Japan	2014	2	^76^
20	Lemont × Jasmine_85	RIL	227	USA	2010	9	^77^
21	CR071 × Jin23b	BC	239	China	2020	47	^10^
22	Qingguai3 × Jin23b	BC	237	China	2020	27	^10^
23	Lemont × Jasmine_85	RIL	227	USA	2015	6	^78^
24	IR64 × Azucena	DH	111	Thailand	2010	8	^79^
25	Zhenshan_97 × Minghui_63	RIL	241	China	2008	29	^80^
26	Lijiangxintuanheigu × Sa0169	F2	122	Taiwan	2022	2	^81^
27	Danteswari × Dagaddeshi	BC	122	India	2017	5	^82^^]^
28	Kahei × Owarihatamochi	BC	158	Japan	2001	2	^83^
29	Ingngopportinawon × Koshihikari	BC	124	Japan	2014	1	^84^
30	Nekken2 × Hokuriko193	F2	193	Japan	2017	3	^85^
31	Kdml_105 × Jhn	RIL	587	Thailand	2006	7	^86^
32	Way_rarem × Oryzica_lianos5	BC	123	Indonesia	2011	16	^87^
33	*Oryza rufipogo*n × Mr219	BC	261	Malaysia	2012	9	^88^
34	*Oryza rufipogon* × Mr219	BC	31	Malaysia	2012	13	^88^
35	*Oryza minuta* × Junambyeo	BC	112	Bangladesh	2011	5	^89^
36	Ingr15002 × Bpt5204	BC	188	India	2020	7	^9^
37	Tarommahalli × Khazar	F2	192	Iran	2011	7	^90^
38	IR64 × Azucena	DH	105	France	2003	9	^91^
39	Urn12 × Koshihikari	BC	39	Japan	2006	2	^92^
40	Heikezijing × Suyunuo	RIL	166	China	2010	22	^93^
41	CT9993 × Kdml105	RIL	141	Thailand	2002	5	^94^
42	Laka × Kencanabali	RIL	126	Indonesia	2016	1	^95^
43	IR64 × Jhn	F2	282	Thailand	2010	4	^96^
44	Lemont × Teqing	RIL	280	USA	2002	9	^97^
45	Bala × Azucena	RIL	205	Bangladesh	2005	21	^98^
46	Gigantevercelli × Vialonenano	F2	114	Italy	2016	2	^99^
47	Tianjingyeshengdao × Co39	RIL	363	China	2012	22	^100^
48	Jaohomnin × Rd6	BC	98	Thailand	2010	2	^101^
49	Indica9024 × Japonicalh422	BC	194	USA	1995	47	^102^
50	Zyo8 × Jx17	DH	127	China	2004	124	^103^
51	93_11 × Nipponbare	RIL	259	China	2013	7	^104^
52	Norin29 × Chubu32	F2	149	Japan	2002	2	^105^
53	Sanhuangzhan2 × Lijiangxintuanheigu	RIL	1123	China	2011	11	^105^

In our study, all the chromosomes were projected with > 10 initial QTLs; hence, we followed Veyriera's approach for MQTL identification. A model with the lowest Akaike information criterion (AIC), corrected AIC (AICc), AIC3, Bayesian information criterion (BIC) and average weight of evidence (AWE) criteria was chosen for MQTL projection. Among the 737 initial QTL, only 435 QTLs were used in the meta-QTL projection due to low LOD scores and/or low PVE% and a large CI^13^ (Fig. S1). As many as 71 MQTLs were distributed across all chromosomes, with each MQTL consisting of one to 19 initial QTLs projected (Fig. 2). Interestingly, the confidence interval of the MQTL region decreased to an extent of 0.04 Mb, while as many as 60 MQTLs showed a confidence interval ≤ 2 Mb. The significance of each MQTL was depicted by its weight, which ranged from 0.03 to 0.62. A total of 31 MQTLs were associated with weight > 0.15 (Table S1). Among these, the top fifteen MQTLs with a confidence interval ≤ 2 Mb and a weight > 0.15 were considered highly significant (Table 2).

**Table 2:** List of the 15 significant MQTLs associated with rice blast resistance.

MQTL	Chr	Position	Peak/Nearest marker	Weight	No. of genes in the interval	No. of original QTLs	Interval (Mb)	Genes related to rice blast resistance and RGA
M-QTL3.1	3	47.32	RM14875	0.19	47	10	0.39	Os03g0328000
M-QTL3.2	3	56.92	RM3033	0.18	27	8	0.19	Os03g0364400 (OsBBS1)
M-QTL3.4	3	108.76	RM6053	**0.40**	124	19	1.12	Os03g0674700 (OsGRF9), Os03g0680800 (OsBIHD1)
M-QTL4.5	4	115.89	RM17377	**0.38**	53	11	0.43	Os04g0573200 (SOD), Os04g0578000 (ACS2)
M-QTL6.3	6	38.58	RM19274	0.16	68	5	1.20	Os06g0286500 (Nbs1-Pi9) Os06g0286700 (Piz)
M-QTL6.4	6	62.73	RM20058	0.19	46	7	0.83	–
M-QTL6.7	6	110.28	RM20593	**0.27**	6	11	0.04	–
M-QTL8.4	8	87.74	RM23224	**0.35**	18	10	0.19	Os08g0448000 (4CL5)
M-QTL8.5	8	102.28	RM23434	0.19	114	5	0.78	Os08g0508800 (Lox2:Os:1), Os08g0509600 (WFP), Os08g0511700 (pi55-1), Os08g0512200 (pi55-2)
M-QTL9.2	9	38.49	RM24030	0.16	26	3	0.26	Os09g0327575, Os09g0327600 (Pi3), Os09g0327800 (Pi3)
M-QTL9.3	9	54.16	RM24236	**0.33**	62	8	0.90	Os09g0396900 (OsVIT2)
M-QTL9.4	9	83.38	RM24721	**0.25**	74	7	0.48	Os09g0533600 (OsRLCK278)
M-QTL10.3	10	85.01	RM25833	**0.22**	143	4	0.92	Os10g0536700 (prx128), Os10g0537800, Os10g0541000 (OsMLO4), Os10g0542800 (OsBSK1-2), Os10g0548200 (OsBSR-K1), Os10g0548300 (OsPUB53), Os10g0548700 (RLCK306), Os10g0552400 (OsPUB67), Os10g0552600 (OsHyPRP18), Os10g0552700 (OsHyPRP19)
M-QTL11.6	11	74.56	RM26835	0.19	42	18	0.68	–
M-QTL12.7	12	98.6	RM28598	0.15	76	6	0.84	Os12g0593000

Significant values are in bold.

### Mining candidate genes within the MQTL regions

Using the flanking positions of each of the 71 MQTL regions, putative candidate genes linked to rice blast disease resistance were mined. In total, 8565 genes were identified within the MQTL region on all chromosomes according to the IRGSP 1.0 *O. sativa* japonica group genome. MQTL2.3 encompassed a maximum of 416 genes, while MQTL6.7 consisted of only 6 genes (Fig. S2). MQTLs were mined for resistance (R) gene analogs (RGAs) based on gene descriptions to obtain 48 characterized RGA candidates for blast resistance in 21 different MQTLs (Table S2). The RGA candidates coding for NBS–LRR-type resistance proteins on chromosome 11 included the characterized blast resistance genes RGA4 (*Pia*) and RGA5 (*Pia*) in MQTL11.1, *PB1* in MQTL11.7, *Pikh* and *Pi54* in MQTL11.8 and *Pik* in MQTL11.9. Genes encoding proteins similar to NBS–LRR-type resistance proteins, such as r*NBS41*, *YR48 Nbs1-Pi9*, *RGA3, RPP8* and *DRP-RP13,* were found in MQTL2.1, MQTL5.3, MQTL6.3, MQTL11.1, MQTL11.8 and MQTL11.9, respectively. The genes deciphering the LRR domain-containing proteins *LRR-RLK* (MQTL1.5), *OsIRL2* (MQTL2.3), *Os_F0767* (MQTL2.5), *OsFbox227* (MQTL4.4), *OsFbox316* (MQTL6.6), and *OsFbox661* (MQTL12.5) were also found. The LRR receptor-like kinase genes *Docs1* and *OsRPK1* were found in MQTL2.1 and MQTL5.3, respectively. Other receptor-like kinases involved in disease resistance, such as RLCK306 and OsLSK1, were found on chromosomes 1 and 10. Similarly, genes encoding NB-ARC domain-containing proteins, such as *Xa39, YR1, OsOSC12, PB1, RPP13*-like protein 3 and *YR2/6/9/10/21/23*, were identified. Pathogenesis-related TF/ERF domain-containing genes (*OsPR10a*, *OsERF#045, OsWR4, Sub1C, AP2/EREBP#127*, and *OsERF#057*) were found on six MQTLs. Furthermore, elicitor-induced defense response-related genes such as *OsRLCK278*, *OsERF#039, Rpp17, UBC5B*, and *TPC1* were found within the identified MQTL regions. The genes encoding blast-induced MAPK signaling (*OsMPK20-4*), jasmonic acid-mediated defense signaling (*OsRab11*), and membrane-trafficking proteins involved in blast resistance (*OsVAMP714*) were found in MQTL1.5, MQTL6.5, and MQTL10.1, respectively. The pathogen-resistance protein *PBZ1,* encoded by *OsPR10a,* was identified in MQTL12.6. However, 35 different MQTLs were associated with as many as 125 uncharacterized RGA (Table S3). These 125 uncharacterized RGA included proteins similar to *NBS–LRR* (*NLR*), encoded by nine genes (six MQTLs); *LRR kinase* and domain-containing proteins, encoded by 26 genes (16 MQTLs); proteins similar to *LRR, encoded* by 17 genes (nine MQTLs); *NB-ARC* domain-containing proteins, encoded by 37 genes (26 MQTLs); and proteins similar to those encoded by 11 genes (three MQTLs). They also included plant disease resistance protein domains and other important yet uncharacterized RGA. Furthermore, *Os10g0163040* in MQTL 10.1 was associated with a protein similar to the blast resistance protein.

Race-specific and broad-spectrum clusters of resistance (R) and/or (RGAs have been reported on rice chromosomes 1, 2, 4, 6, 11 and 12^10,20–22^. Similarly, a few genes appeared to be in pairs or clusters within the identified MQTLs. For example, five genes (*Os11g0224900/RGA3, Os11g0225100/RGA4/Pia, Os11g0225300/RGA5/Pia, Os11g0211300, and Os11g0213800*) were identified in MQTL11.1; three genes (*Os11g0493700, Os11g0492900* and *Os11g0494100)* were identified in MQTL11.5; two genes (*Os11g0639100/Pi54/PIKH* and *Os11g0633500/RPP8*) were identified in MQTL11.8; and three genes (*Os11g0689100/PIKM/PIK, Os11g0688832/PIKM/PIK* and *Os11g0676200*) were identified in MQTL11.9 (Tables 2, S3). Furthermore, clusters (≥ 5 genes) of different classes of RGAs, including those encoding NLR, NB-ARC*,* disease resistance proteins, and LRR proteins, were also observed. A cluster of five RGAs was observed within each of the MQTL1.5, MQTL4.4, MQTL5.3 and MQTL10.1. Similarly, clusters of six genes were observed within MQTL8.3 and MQTL12.6, while seven and eight RGAs were clustered within MQTL2.3 and MQTL12.2, respectively. Furthermore, five clusters were identified within chromosome 11 within MQTL11.1 (14 RGA), MQTL11.5 (7), MQTL11.7 (13), MQTL11.8 (6) and MQTL11.9 (21).

**Figure 3:**
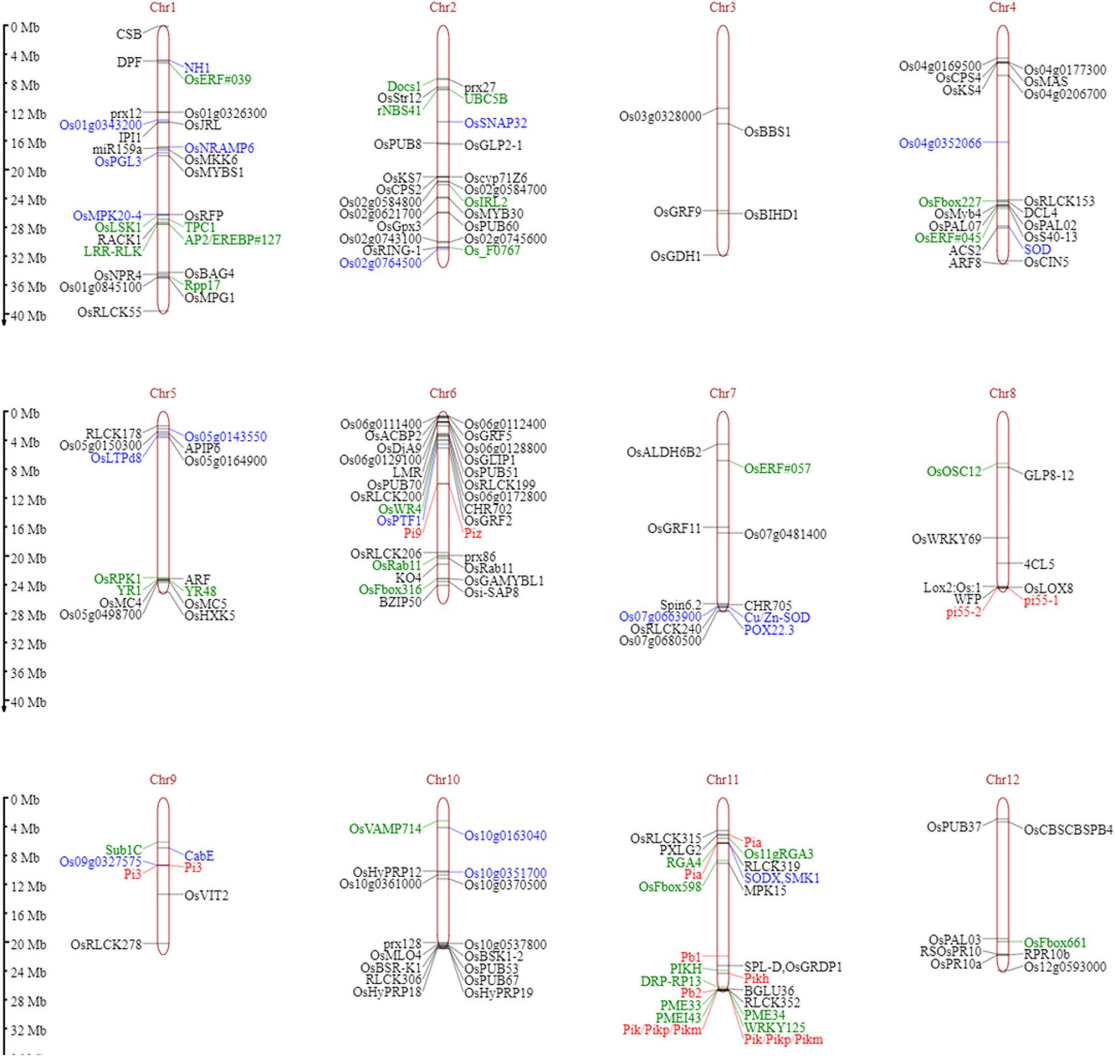
Representation of MQTLs harboring genetic loci associated with blast resistance. Loci represented in red indicates characterized blast resistance genes, green indicates RGAs, blue indicates genes associated with blast resistance as per the gene description, and black indicates loci with trait ontology associated with blast resistance.

Genes specific to rice blast resistance were identified through a custom search in the Rice Annotation Project database (RAP-DB). Based on the reported characterized gene names, gene descriptions, and trait ontologies associated with blast resistance and characterized RGAs, a total of 199 candidate genes within 53 MQTLs were identified (Fig. 3, Table S4). These genes were associated with different defense mechanisms, such as MAPK signaling (e.g., *OsMPK20-4*/MQTL1.5), JA-induced defense response (*NH1*/MQTL1.2), *R* genes (*Nbs1-Pi9/Piz/*MQTL6.3), their analogs (e.g., *OsRFP*/MQTL1.5), and elicitors, such as PAMP (e.g., *OsDjA9*/MQTL6.1; *OsRLCK55*/MQTL1.8). A total of 13 characterized genes for blast resistance on MQTLs located on chromosomes 6, 8, 9 and 11 were identified in our study (Table 3). Seven of these genes were associated with significant MQTLs (weight > 0.15): *Os06g0286700/Piz* and *Os06g0286500/Nbs1-Pi9* in MQTL6.3; *Os08g0511700/pi55-1* and *Os08g0512200/pi55-2* in MQTL8.5; *Os09g0327600/Pi3 (Pi5-1)* and *Os09g0327800/Pi3 (Pi5-2)* in MQTL9.2; and *Os11g0639100/Pi54/Pikh* in MQTL11.8 for blast resistance. Moreover, six genes characterized for blast resistance were identified within MQTL11.1 (*Os11g0225300/Pia/RGA4* and *Os11g0225100/Pia*/*RGA5*), MQTL11.7 (*Os11g0598500/Pb1*) and MQTL11.9 (*Os11g0682600/Pb2, Os11g0689100/Pik* and *Os11g0688832/Pik*). Among the 15 most significant MQTL intervals, a total of 887 genes were identified, including 29 candidates for rice blast resistance (Table 2). These top 15 most significant MQTL regions included six characterized genes (*Piz, Nbs-Pi9, pi55-1, pi55-2, Pi3/Pi5-1* and *Pi3/Pi5-2*). Furthermore, they included other important genes, such as *Os10g0548700/RLCK306, Os10g0552400/OsPUB67*, and *Os10g0548300/OsPUB53,* in MQTL10.3; these genes are associated with PUB proteins with U-box domains (Table S4).

**Table 3:** Details of meta-QTL regions associated with characterized blast resistance genes.

Meta-QTL	Weight	Peak marker	MQTL interval (Mb)	Gene stable ID	Gene name	Gene description and gene synonyms
M-QTL6.3	**0.16**	RM19801	1.21	*Os06g0286700*	*Piz*	Similar to *Piz(t)*
				*Os06g0286500*	*Nbs1-Pi9*	Similar to NBS–LRR disease resistance protein homolog
M-QTL8.5	**0.19**	RM23442	0.78	*Os08g0511700*	*pi55-1*	LRR protein; candidate gene for *pi-55*
				*Os08g0512200*	*pi55-2*	Heavy metal associated domain; candidate gene for* pi-55*
M-QTL9.2	**0.16**	RM24030	0.26	*Os09g0327600*	*Pi3*	Similar to *Pi5-1*
				*Os09g0327800*		Similar to *Pi5-2*
M-QTL11.1	0.11	RM26220	1.72	*Os11g0225100*	*Pia/RGA4*	Nucleotide binding site-leucine rich repeats (NBS–LRRs) protein; RGA5, *Pia-1*
				*Os11g0225300*	*Pia/RGA5*	NBS–LRR protein, RGA4, *Pia-2*
M-QTL11.7	0.06	RM27016	0.96	*Os11g0598500*	*Pb1*	Pb1-like protein; NB-ARC domain containing disease resistance protein; *Pb1*
M-QTL11.8	**0.20**	RM4112	1.25	*Os11g0639100*	*Pikh/Pi54*	NBS–LRR protein, Broad spectrum resistance against *M. oryzae*; *Pi-kh*, *Pi54*
M-QTL11.9	0.13	RM27285	1.40	*Os11g0682600*	*Pb2*	NB-ARC domain containing disease resistance protein, Panicle blast resistance
				*Os11g0689100*	*Pik/Pikp/Pikm*	Coiled-coil NBS–LRR (CC-NBS–LRR) disease resistance protein; *Pikp-2*, *Pikm6-NP*, *Pik6-NP*
				*Os11g0688832*		CC-NBS–LRR disease resistance protein; *Pikp-1*, *Pikm5-NP*, *Pik5-NP*

Significant values are in bold.

### Ontology of genes within the MQTL region

**Figure 4:**
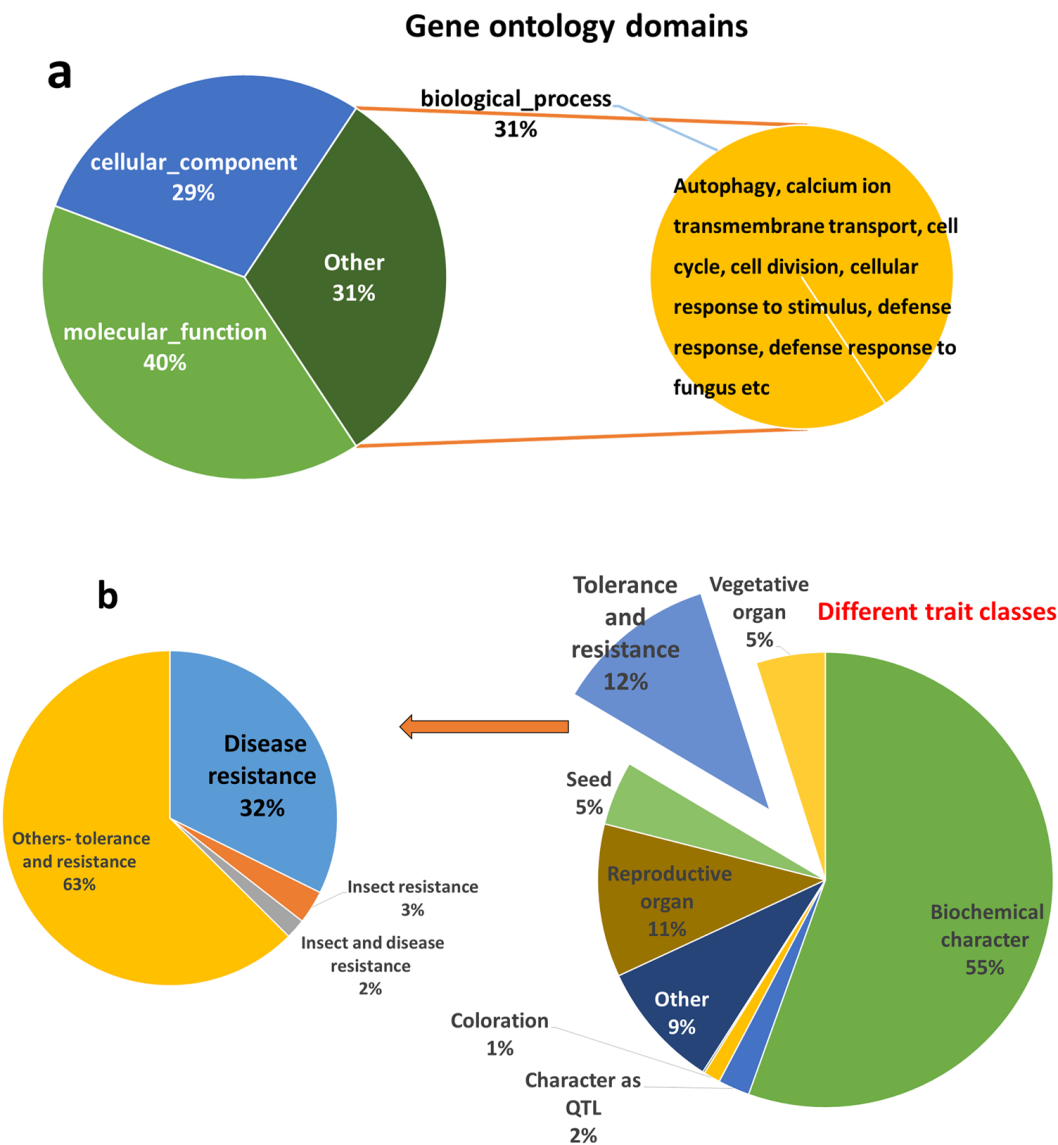
Different (**a**) Gene Ontology terms and (**b**) trait classes associated with genes within M-QTL regions.

Gene ontologies of all the genes within MQTL regions were retrieved using the Ensembl database. Approximately 40% of the genes were associated with molecular functions, 29% were associated with cellular components, and 31% were associated with biological processes. The GO annotations of some of the genes involved in the biological processes included “autophagy”, “calcium ion transmembrane transport”, “cell cycle”, “cell division”, “cellular response to stimulus”, “defense response”, and “defense response to fungus”, which may have a role in rice blast disease resistance” (Fig. 4a). Furthermore, 12% of the genes were assigned to the “tolerance and resistance’ trait class, which included 32% of the disease resistance-conferring genes (Fig. 4b).

**Figure 5:**
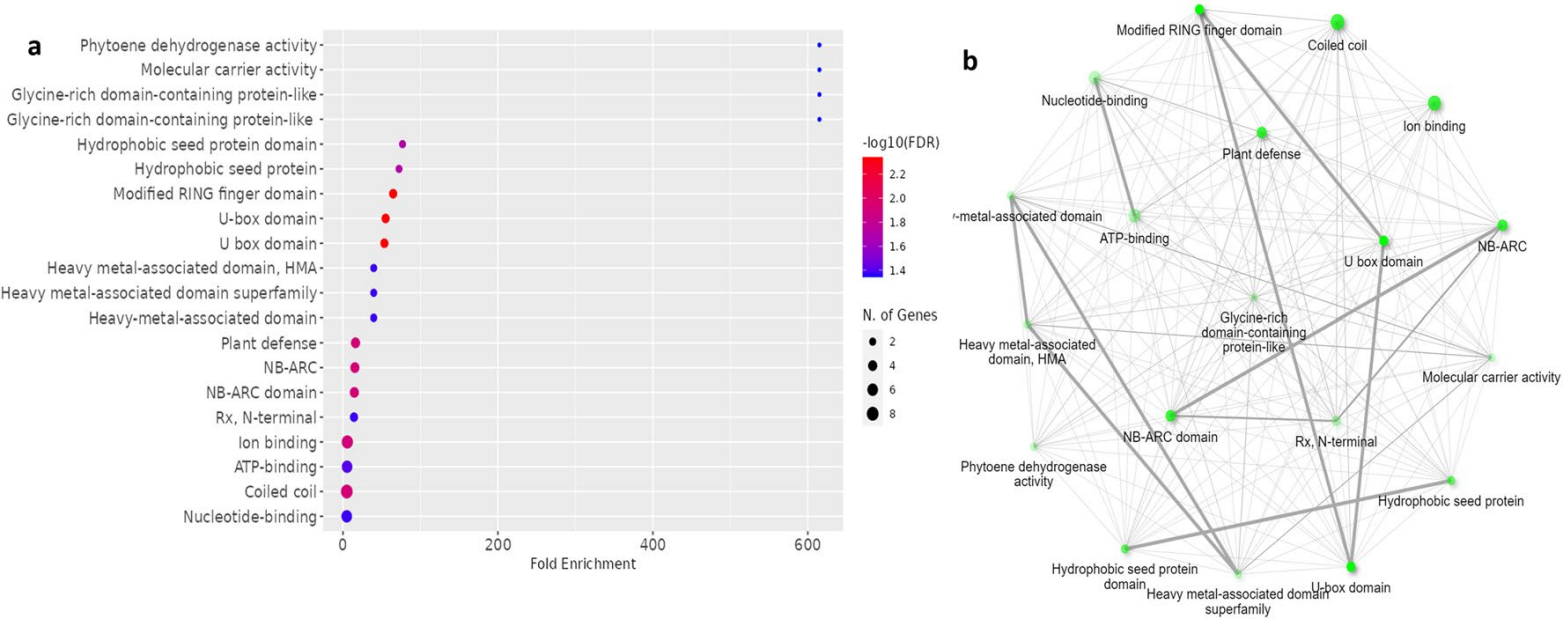
Functional annotation of genes related to rice blast resistance within the top 15 MQTLs. (**a**) Representation of the number of genes involved in different functional annotations (domain/motif/pathway) with fold enrichment. (**b**) network of genes involved in the expression of a function (domain/motif/pathway).

The functional annotation of 199 candidate genes related to rice blast resistance revealed their significant involvement in 30 different functions (Table S5) with their fold enrichment, and an FDR < 0.05. These genes were predominantly associated with gene networks related to the response to stimulus, the response to stress and the defense response, protein modification, signal transduction, and the diterpenoid and cinnamic acid biosynthetic/metabolic pathways. The number of genes shared among the functional annotations was represented by the network and clustering of pathways (Fig. S3). The genome-wide fold enrichment of the candidate genes indicated three enriched regions, two on chromosome 11 (MQTL11.8 and 11.9) and one on chromosome 6 (MQTL6.3), which were associated with genes characterized for blast resistance (Fig. S4). Furthermore, we considered only the 29 rice blast candidate genes within the 15 most significant MQTLs for functional annotation. Genes within eight of these MQTLs were associated with 11 different pathways (Fig. 5). The genes within MQTL6.3 and MQTL9.2 were associated with the nucleotide-binding adaptor (NB-ARC) shared by APAF-1, R proteins, and CED-4 domain, while those within MQTL8.5 were involved in the heavy metal-associated domain superfamily. Furthermore, the *Os09g0327600/Os09g0327800/Pi3* genes in MQTL9.2 and the *Os10g0542800/OsBSK1-2* genes in MQTL10.3 were affiliated with the plant defense pathway (Table 4).

**Table 4:** Functional annotation associated with rice blast resistance genes within the top 15 MQTLs.

Enrichment FDR	nGenes*	Total genes†	Fold enrichment	Functional annotation (domain/motif/pathway)	Genes	M-QTL
0.005	3	69	53.480	U box domain	OS10T0548300-01 OsJ_32366 OsJ_32402	M-QTL10.3
0.005	3	57	64.739	Modified RING finger domain	OS10T0548300-01 OsJ_32366 OsJ_32402	M-QTL10.3
0.012	8	1988	4.950	Coiled coil	OsJ_12119 b29O05.5 OS09T0327600-01 OS09T0327800-00 OsJ_32362 OS10T0548300-01 OsJ_32366 OsJ_32402	M-QTL10.3, M-QTL9.2
0.012	4	304	16.185	Plant defense	b29O05.5 OS09T0327600-01 OS10T0541000-01 BSK1-2	M-QTL9.2, M-QTL10.3
0.012	4	335	14.687	NB-ARC domain	b29O05.5 OS06T0286700-00 OS09T0327600-01 OS09T0327800-00	M-QTL6.3, M-QTL9.2
0.013	7	1530	5.628	Ion binding	GRF9 CCS 4CL5 CM-LOX1 SPL14 OS09T0396900-01 BSK1-2	M-QTL3.4, M-QTL8.4, M-QTL10.3, M-QTL9.3
0.019	2	32	76.877	Hydrophobic seed protein domain	OS10T0552600-01 OsJ_32400	M-QTL10.3
0.039	6	1390	5.310	ATP-binding	OS03T0364400-01 b29O05.5 4CL5 OS09T0327600-01 OsJ_30123 BSK1-2	M-QTL3.2
0.042	2	62	39.679	Heavy metal-associated domain superfamily	CCS OS08T0512200-00	M-QTL8.5
0.042	3	259	14.248	Rx, N-terminal	b29O05.5 OS09T0327600-01 OS09T0327800-00	M-QTL9.2
0.042	6	1516	4.868	Nucleotide-binding	OS03T0364400-01 b29O05.5 4CL5 OS09T0327600-01 OsJ_30123 BSK1-2	M-QTL3.2, M-QTL8.4, M-QTL9.2, M-Q TL10.3

*number of query genes involved in the function.

^†^The total number of genes in the gene network involved in functional annotation.

### Validation of MQTLs

**Figure 6:**
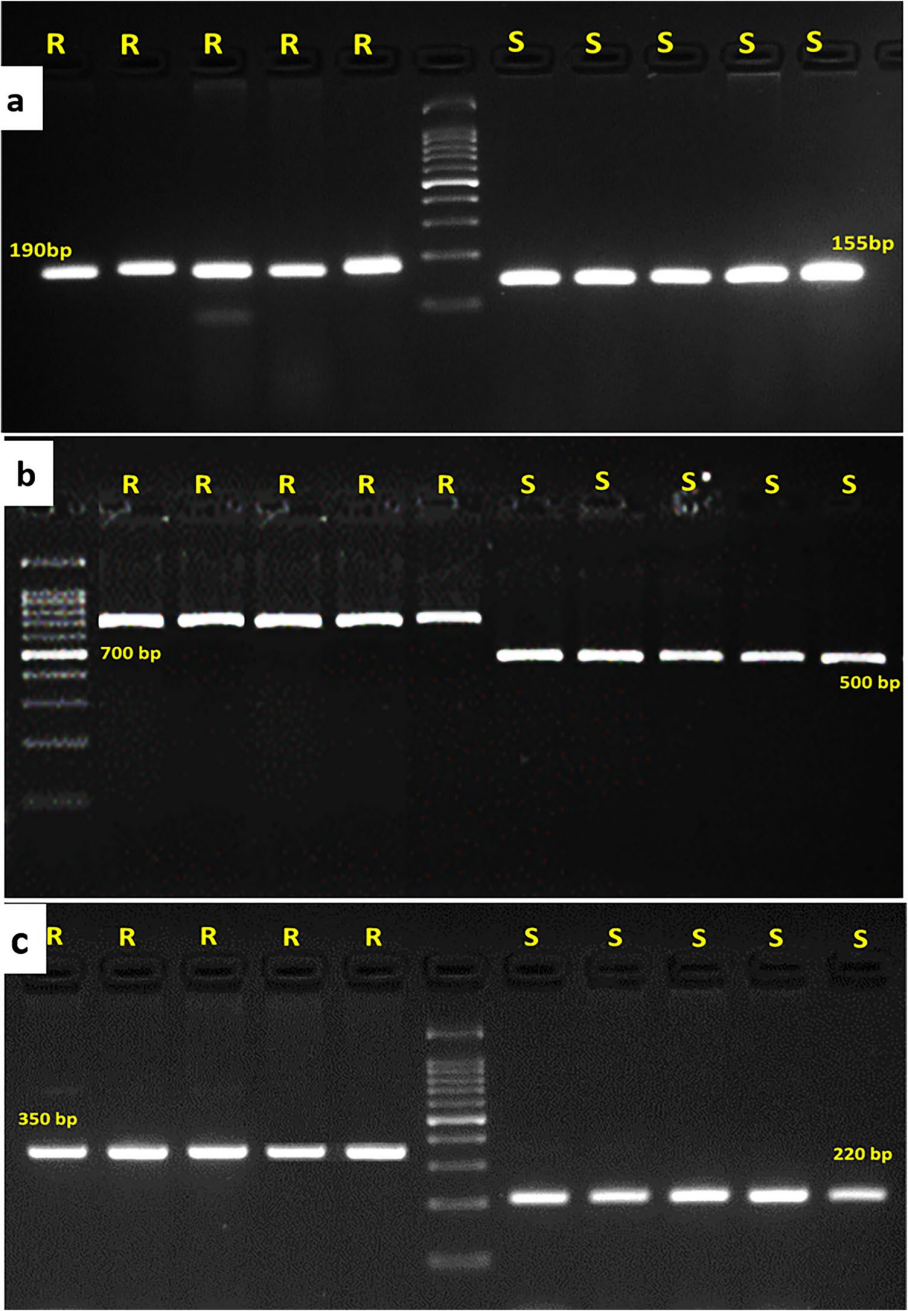
Validation of the peak markers of three significant MQTLs for their association with blast disease resistance. Polymorphism between the five-blast disease-resistant (R) and susceptible (S) contrasts for the peak markers (**a**) RM17377 (MQTL4.5), (**b**) 40N23r (MQTL9.2) and (**c**) Pikh (MQTL11.8).

Five blast-resistant rice genotypes (mean score ≤ 4.5) and five susceptible genotypes (mean score ≥ 7.5) were genotyped using peak/nearest markers associated with each of the significant MQTLs (Table S1). Three of the peak markers, viz., RM17377, 40N23r and Pikh, in MQTL4.5, MQTL9.2 and MQTL11.8, respectively, differentiated resistant and susceptible groups (Figs. 6, S5). Among them, 40N23r and Pikh were reported to be gene based markers for the *Pi5* and *Pikh/Pi54* genes, respectively^23^. Thus, validation of these peak markers confirmed the significance of the corresponding MQTLs in blast disease resistance.

**Figure 7:**
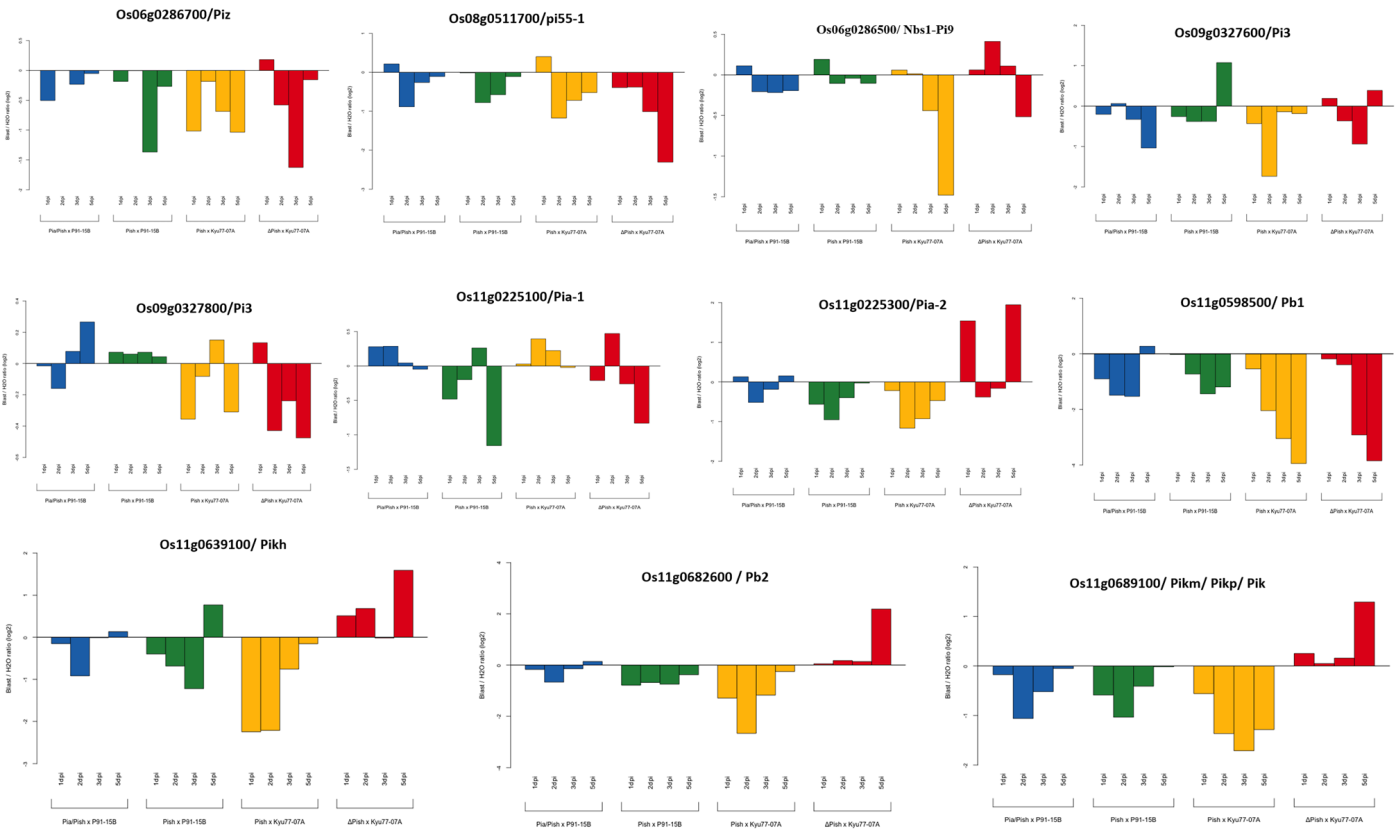
Graphical representation of the fold change in the expression of characterized blast resistance genes within significant MQTLs at 1, 2, 3- and 5-days post inoculation (dpi) of leaves with two *M. oryzae* strains from RiceXPro. *Pia/Pish* × P91-15B and *PISH* × Kyu77-07A indicate incompatible (resistant) reactions, and *Pish* × P91-15B and ∆PISH × Kyu77-07A indicate compatible (susceptible) reactions. P91-15B and Kyu77-07A are *M. oryzae* strains, while Pia, Pish and ∆PISH represent Nipponbare (NB) genotypes harboring the corresponding genes (*Pia* and *Pish*).

In silico validation of the candidate genes associated with rice blast resistance was performed by screening for microarray-based expression studies. The expression profiles of 150 blast resistance candidates identified within different MQTLs in our study were retrieved from the RXP_3001 dataset (gene expression profile in whole leaves inoculated with *M. oryzae*) available in the RiceXPRO database (https://ricexpro.dna.affrc.go.jp/)^24^. The expression patterns of candidate genes at 1, 2, 3, and 5 days post inoculation (dpi) with two *M. oryzae* strains (P91-15B and Kyu77-07A) in the differential Nipponbare entries harboring the blast resistance genes *Pia* and *Pish* were examined. Among the 199 candidate genes, the expression profiles of 73 were not available in the database. Within the expression profiles of 126 genes, 51 were upregulated and 33 were downregulated at 1, 2, 3, and 5 dpi with both compatible and incompatible reactions (Fig. S6). The gene *Os02g0584700* in MQTL2.3 showed differential expression between compatible and incompatible reactions with both strains, while the gene *Os06g0163000/OsPUB70* in MQTL6.2 discerned differential expression with the Kyu77-07A strain (Table S3; Fig. S6). However, 42 genes, including eleven characterized genes, exhibited differential expression at different dpi. The genes *Os09g0327600* and *Os06g0286700*, which were infected with P91-15B and Kyu77-07A, respectively, were downregulated at 5 dpi in incompatible reactions. The expression of *Os08g0511700* and *Os09g0327600* in both of these strains was upregulated at 1 dpi and downregulated at 5 dpi, respectively. Furthermore, the expression of the *Os09g0327800* gene was downregulated in the resistant combinations but upregulated in the susceptible combinations at 1 dpi with Kyu77-07A and at 2 dpi with the P91-15B strain (Fig. 7). A similar trend was observed for *Os11g0225300/Pia-2*, whose expression was downregulated in the resistant combinations but upregulated in the susceptible reactions at 1 dpi and 5 dpi with Kyu77-07A; the reverse was true for the P91-15B strain. *Os11g0225100/Pia-1* was upregulated in the incompatible reaction but downregulated in the compatible reaction at 1 dpi and 2 dpi with the P91-15B strain. The gene *Os11g0598500/Pb1* was downregulated in all reactions except for the resistance reaction with the P91-15B strain at 5 dpi. The genes *Os11g0639100/Pi54/Pikh, Os11g0682600/Pb2* and *Os11g0689100/Pik/Pikm/Pikp* were downregulated in the resistant reaction but upregulated in the susceptible reaction in all four treatments with the Kyu77-07A strain. However, the expression of these genes was unaltered in response to treatment with the P91-15B strain (Fig. 7).

### Orthology of genes identified in the MQTL region with other plant species

**Figure 8:**
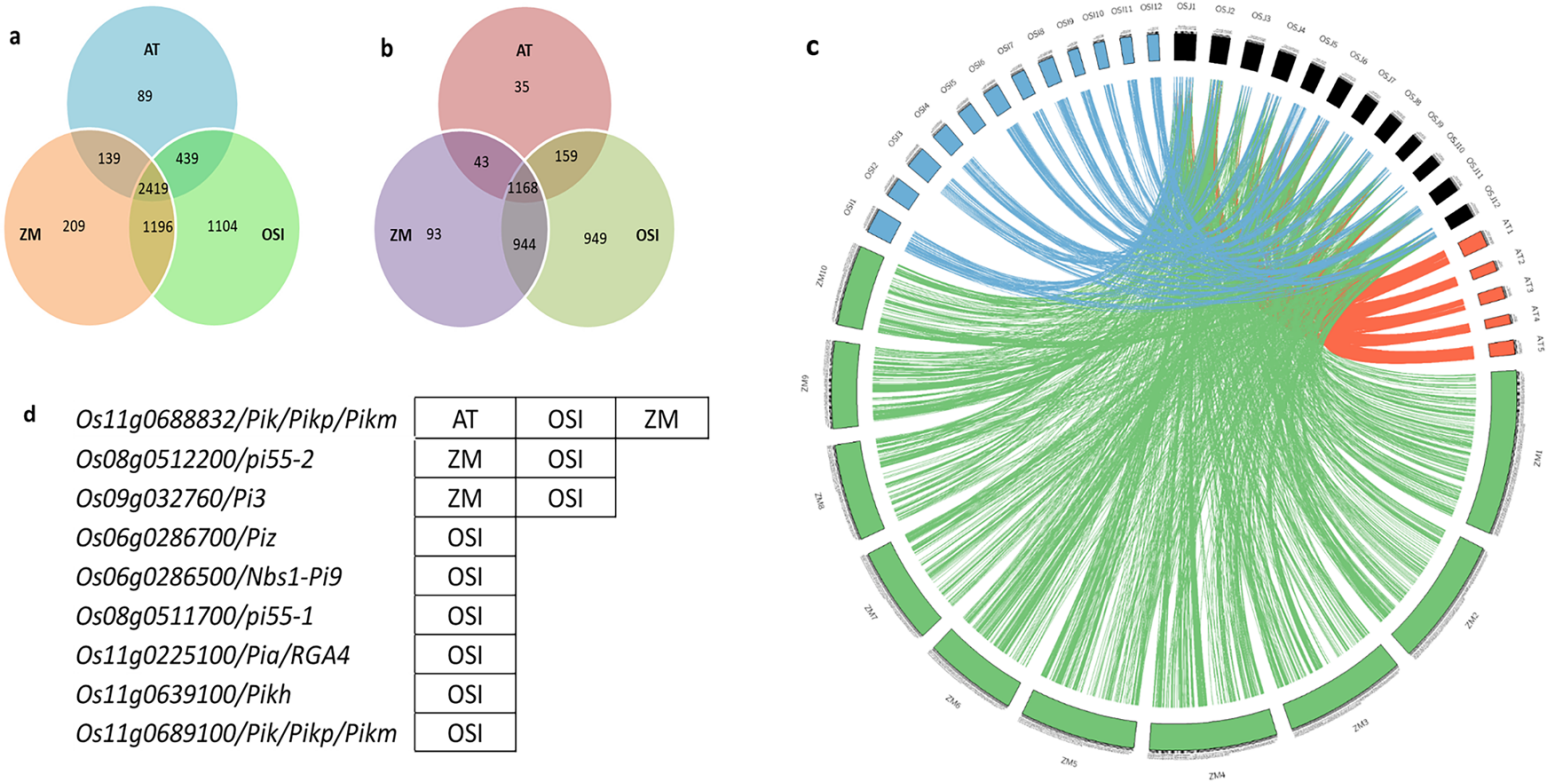
Number of *O. sativa* japonica group genes within the MQTL region showing (**a**) orthologous relationships (**b**) high orthology (= 1) with OSI- *O. sativa* indica, ZM- *Zea mays* and AT- *Arabidopsis thaliana.* (**c**) Circular diagram representing the orthology of identified genes (within the M-QTL region) with other plant species. The outer circle represents the chromosomes of OSJ (black), OSI (blue), ZM (green) and AT thaliana (red) under comparison. (**d**) Characterized genes showing orthology with related species.

The query genes (8318) within the MQTL region were identified from the data available from the IRGSP genome of the *O. sativa* japonica (OSJ) group. A total of 5595 OSJ genes exhibited differential orthologous relationships with *O. sativa* indica (OSI), *Zea mays* (ZM), and *Arabidopsis thaliana* (AT) (Fig. 8). Among these, 3391 genes showed high confidence in orthologity (= 1) with one or more of the three genomes. Furthermore, nearly half of the query genes were orthologous to all three genomes (AT, OSI, and ZM). As expected, most of the OSJ genes were orthologous to OSI (5158), followed by ZM (3963) and AT (3086) (Fig. 8a). Among the 199-rice blast resistance candidate genes in the OSJ genome, AT, ZM, and OSI had 106, 141, and 168 orthologs, respectively. The orthologity of characterized blast resistance genes found in the intervals MQTL6.3, MQTL8.5, MQTL9.2, MQTL11.1, MQTL11.7, MQTL11.8 and MQTL11.9 was analyzed using the g:Profiler online tool. Among the thirteen characterized genes, only *Os11g0688832/Pik/Pikp/Pikm* was orthologous to the AT, ZM and OSI genes. Two genes (*Os08g0512200/pi55-2* and *Os09g0327600/Pi3*) were orthologous to the Zm and OSI genes, while six genes (*Os06g0286700/Piz, Os06g0286500/Nbs1-Pi9, Os08g0511700/pi55-1, Os11g0225100/Pia/RGA4, Os11g0639100/Pi54/Pikh* and *Os11g0689100/Pik/Pikp/Pikm*) were orthologous to the OSI genes only. Furthermore, the gene *Os08g0512200/pi55-2* showed high orthology with both ZM and OSI (Fig. 8).

## Discussion

Sustained rice production to meet global food security needs has gained paramount importance with the increasing population. Rice productivity is significantly constrained by pests and diseases. These findings have led to the convergence of rice researchers’ focus on resistance breeding against these constraints. Among several diseases, blast disease is one of the most devastating diseases in rice, causing yield losses of up to 100%. More than 100 genes and 350 QTLs conferring blast resistance have been identified, 37 of which have been cloned and characterized^1,9,10^. Most of these genes are race specific and impart highly fragile vertical resistance against blast disease. Diverse rice germplasms, including cultivars, landraces and wild species, are being screened for genes contributing to broad-spectrum resistance^25^. Disease resistance conferred by one or two major genes is effective but prone to breakdown with the emergence of new strains. Hence, gene clusters that provide broad-spectrum resistance and thereby impart horizontal durable resistance should be focused on. QTLs linked to blast resistance are likely to harness horizontal resistance, which is durable^15^. However, the effectiveness of QTLs in MAS is limited by several factors, such as linkage drag due to the large QTL interval, the QTL projection model, and limited recombination events in the biparental mapping population^15^. Moreover, the instability of QTLs identified in a particular mapping population prevents their deployment in MAS across genetic backgrounds. Fine-mapping the QTLs and delimiting them to one or two genes, such as *Pb-bd1* or *Pi-69(t),* followed by their characterization and utilization in breeding programme is effective^25,26^. However, fine mapping each of the many QTLs is unrealistic because it is time- and resource-demanding^9^. In this regard, the meta-QTL analysis approach could be useful for refining QTL intervals and subsequently validating their association with trait of interest^18^.

In the present study, initial QTLs were available on all the chromosomes, depicting the quantitative genetic architecture of blast disease resistance. A total of 71 MQTLs were projected from 435 initial QTLs reported in 53 independent studies. A reduction in the number of MQTLs from the initial QTLs indicated the colocation of the QTL and shrinkage of their confidence interval. A similar pattern was reported for rice blast^27^ and rice grain weight^15^. Furthermore, approximately 3390 genes within the MQTL region exhibited high orthologity with other subspecies of rice (*O. sativa* indica group), cereal species (*Z. mays*) and *A. thaliana*. The orthology of these genes with related species verified the significance of the respective MQTLs and reiterated their stability across species^28^. The genes underlying the identified MQTLs were affiliated with ontologies such as response to stimulus, defense response, and response to fungus, which indicated their possible role in resistance against rice blast. Resistance to blast disease is conferred by resistance (*R*) genes, such as nucleotide binding site-leucine rich repeats (*NBS–LRR* or *NLR*), nucleotide binding adaptors shared by *APAF-1*, R proteins, and *CED-4 (NB-ARC*), disease resistance proteins (*DRPs*), leucine rich repeats (LRRs) and pathogenesis related proteins (PRs)^20^. The identified MQTL regions consisted of 280 disease resistance genes. Among them, 199 genes (*R* genes and their analogs) within 29 MQTL regions were candidates for blast resistance involved in PAMP-triggered immunity, MAPK signaling and phytohormone signaling. As many as thirteen genes characterized for blast resistance, such as *Piz, Nbs1-Pi9, pi55-1, pi55-2, Pi3, Pia/RGA4, Pia/RGA5, Pb1, Pi54, Pikh, Pb2 and Pik/Pikp/Pikm*^29–36^, resided within the identified MQTL regions. These MQTLs harboring one or more characterized genes could potentially contribute to blast resistance across different genetic backgrounds.

According to the gene ontology, blast resistance candidates within 29 MQTL regions were involved in different defense pathways. For instance, MQTL2.3 consists of the *Os02g0571100/OsCPS2* and *Os02g0570400/OsKS7* genes, which are affiliated with diterpenoid biosynthetic/metabolic pathways that are known to influence blast resistance in rice^37^. Similarly, genes related to the defense response pathway were found within MQTL1.5, 2.3, 4.1, 9.2, 10.3, 11.1, 11.9 and 12.6, which signifies their role in plant defense. Furthermore, thirteen of the MQTLs on chromosomes 1, 2, 4, 5, 8, 10, 11 and 12 consisted of clusters of > 5 RGAs, and MQTL6.3 was enriched with a defense-related gene cluster on chromosome 6. These MQTL regions harboring gene clusters for blast resistance indicate their possible role in broad-spectrum resistance^10,21,22^. Furthermore, gene clusters or pairs of NLRs found on chromosome 11 (MQTL11.1, 11.5, 11.8 and 11.9) have been proven to play a role in broad-spectrum resistance^20^. Introgression of MQTLs with RGA clusters works akin to pyramiding genes in conferring broad-spectrum resistance; thus, introgression is advantageous over that of a single gene^20,21,38^.

The identified highly significant (weight > 15) and high-resolution MQTLs encompassed 887 genes, including 29 rice blast-associated genes. Among the significant MQTLs, MQTL3.1 harbors the gene *Os03g0324600,* which encodes an *NB-ARC* domain-containing protein, while *Os03g0328000* encodes a DOCK family guanine nucleotide exchange factor involved in the defense response against fungus^39^. Similarly, the genes *Os03g0674700/OsGRF9* and *Os04g0578000/ACS2* in MQTL3.4 and MQTL4.5, respectively, are known to enhance resistance against blast disease^40,41^. The characterized genes *Os06g0286700/Piz* and *Os06g0286500/Nbs1-Pi9* in MQTL6.3 and *Os09g0327600/Os09g0327800/Pi3* in MQTL9.2 encode RGA, which is known to confer broad-spectrum resistance against rice blast^10,42,43^. Furthermore, the genes *Os08g0511700/pi55-1* and *Os08g0512200/pi55-2* in MQTL8.5 encode LRR and heavy metal-associated domain-containing proteins, which are candidates for *pi55(t)*^31^. Furthermore, the *Os10g0548300/OsPUB53* gene in MQTL10.3 harbors U-box and modified ring finger domains, indicating its role in the plant defense pathway^44,45^. MQTL11.8 harbors *Pi54/Pikh*, a characterized gene for broad-spectrum resistance against blast^46^. These MQTLs span ≤ 2 Mb intervals and are amenable to marker-assisted selection (MAS)^47^. The peak/nearest markers of significant MQTL4.5, MQTL9.2 and MQTL11.8 were validated for their association with blast disease resistance. Since the ten genotypes utilized for validation were chosen from a diverse set of genotypes, the results can extrapolate to any new set of rice genotypes expecting the similar results. However, one should be careful while extrapolating the findings as that these markers can be assayed only for resistance or susceptibility regulated by corresponding MQTL regions and not for complete resistance to blast disease which in this case is a quantitative trait known to be regulated by several loci. The *in-silico* validation results may be extrapolated to other genotypes based on the presence of corresponding MQTL. Apart from that, the information of MQTLs identified and validated in the present study can be utilized in genomic prediction-based trait improvement programs by considering MQTL information as additive effects while predicting using genomic information. These markers could be used as surrogates in MAS for blast resistance breeding. Furthermore, in silico expression analysis of *Os11g0639100/Pi54/Pikh,* which resides within MQTL11.8, revealed differential expression of the Kyu77-07A strain incompatible and incompatible reactions to leaf infection, which validated the significance of the MQTL. Similarly, the genes within MQTL2.3 (*Os02g0584700*), MQTL6.2 (*Os06g0163000*/*OsPUB70*) and MQTL11.9 (*Os11g0682600*/*Pb2* and *Os11g0689100*/*Pik*) also exhibited similar expression patterns. Furthermore, the validation using peak markers suggests that the markers can be directly utilized to screen germplasm or new breeding lines for identification of resistant lines. Since the ten genotypes utilized for validation were chosen from diverse set of genotypes, the results can extrapolate to any set of genotypes expecting the similar results. However, one should be careful while extrapolating that these markers can be assayed only for resistance or susceptibility regulated by corresponding MQTL regions and not for complete resistance to blast disease which a quantitative trait known to regulated by several loci. On the other hand, the results of the in-silico validation of genes in significant MQTL regions helps to understand the genes regulate resistance and susceptibility. The in-silico validation results may be extrapolated to other genotypes based on the presence of corresponding MQTL. The genes on chromosome 9 and 11 correspond to MQTL 9.2 and MQTL 11.8 were showed to differential expressions in in silico validation. These two MQTL were validated using peak markers for differentiating susceptible and resistant genotypes. Hence, results are validated to have significant scope to utilize in improvement of resistance in rice. Apart from that, the information of MQTLs identified and genes validated in the present study can be utilized in genomic prediction-based trait improvement programs by considering MQTL information as additive effects while predicting using genomic information^47^. The results presented in our study will help researchers decipher the complex network of genes and pathways underlying rice blast resistance. Furthermore, the identified MQTLs have potential utility in blast disease resistance breeding programs.

## Materials and methods

### Literature survey and data collation

An exhaustive bibliographic survey related to QTL mapping for blast resistance in rice has been performed (https://scholar.google.com/, https://www.researchgate.net/) using keywords like rice blast, QTL, mapping, rice blast resistance, blast resistant genomic regions, blast QTL information, blast resistance genes etc. to retrieve the literature. Information on the size, parentage, and type of mapping population, mapping function, LOD score, and PVE (%) was collected for a total of 737 QTLs compiled from 53 studies reported from 14 different countries across the globe (Table 1). The mapping studies included F_2_, doubled haploids (DH), recombinant inbred lines (RIL), and backcross (BC) mapping populations, with population sizes ranging from 31 to 1123. Input files, i.e., genetic map information and QTL information, were prepared as per the requirements of Biomercator V4.2^17,48^. Those studies that lacked genetic map information were excluded from the study. The map information included the details of the type of mapping population and the genetic position of the markers, while the QTL information included the QTL ID, trait, trait ontology ID, experimental year and location, LOD score, PVE (%) value with the QTL position, and confidence interval, as described in the Biomercator V4.2 user guide.

### Consensus map and QTL projection

The consensus map required for QTL projection was developed using Biomercator V4.2 by combining the genetic maps of the published original QTL studies and the reference map of rice available in the Gramene database (https://archive.gramene.org/db/markers/marker_view). Individual map files consisting of map and QTL information for each of the 737 original QTLs were integrated with the consensus map for their projection on the consensus map. Among them, 435 were projected on the consensus map for MQTL projection, while the other initial QTLs were not projected due to low LOD or PVE% and large CI^13^. For the projection of the SNP markers linked to the original QTL, the nearest SSR marker on the reference map corresponding to the position of the SNP was considered^15^. The QTL position and confidence interval of the original QTL were used where available; otherwise, the CI was calculated using the Darvasi and Soller equation^49^, $\text{CI}= \frac{530}{\text{N}*{\text{R}}^{2}}$ for the F_2_ and BC populations and their modifications, $\text{CI}= \frac{287}{\text{N}*{\text{R}}^{2}}$ and $\text{CI}= \frac{163}{\text{N}*{\text{R}}^{2}}$ for the DH and RIL populations, respectively, in which N is the size of the population and R^2^ is the phenotypic variation explained by the QTL^50^.

### Meta-QTL analysis

Meta-QTL analysis was implemented on the consensus map of each chromosome with projected QTLs considering default parameters in Biomercator V4.2. As the initial QTL number was more than nine on each chromosome, the two-step ‘Veyrieras’ method of meta-analysis was used^16^. The Akaike information criterion (AIC), corrected AIC (AICc), AIC3, Bayesian information criterion (BIC) and average weight of evidence (AWE) were used to choose the best-fit model. The model with the lowest AIC value is the best-fit model, as it indicates the least loss of information^27^. A best-fit model was chosen, and the other parameters were retained as defaults to obtain meta-QTLs. Based on the information from the best-fit model, the significant MQTLs are listed along with the complete information. The MQTL weights generated by the software were used to sort the identified MQTLs into significant MQTL.

### Validation of MQTLs

The peak or nearest marker to the position of the significant MQTL was considered for validation (Table S1). The sequences of these primers were retrieved from the Gramene Marker database (https://archive.gramene.org/markers/) and synthesized for use in marker assays. Ten genotypes with differential responses to blast disease were selected from one of our experiments at the ICAR-NRRI and evaluated over two years (unpublished; Table S6). Disease scoring was performed following a standard protocol, and entries with a mean disease score ≤ 5.5 were considered to indicate quantitative resistance^51^. For the validation of peak markers of the significant MQTLs, we considered five entries with a mean disease score ≤ 4.5 as resistant and five entries with a mean disease score ≥ 7.5 as susceptible. DNA was extracted from leaf samples of ten genotypes using the CTAB method^52^. Genomic DNA was subjected to thermal amplification using corresponding primers (peak markers), and the amplified product was visualized through gel electrophoresis and a documentation system. The markers that differentiated the resistant and susceptible entries validated the corresponding MQTLs for blast resistance.

### Mining candidate genes within the MQTL region

The physical position of the flanking markers for each MQTL on the consensus map was identified using a reference map. Using the ‘Biomart’ tool of Ensembl Plants^53^ and the Gramene database (https://archive.gramene.org/plant_ontology/index.html), reported genes within the MQTL region were downloaded using the IRGSP 1.0 *O. sativa japonica* genome as a reference. The following gene sequences were used to identify RGA genes: NBS–LRR, LRR containing protein or kinase, MAPK, pathogenesis-related, NB-ARC domain containing genes, and elicitor protein. The gene description, gene ontology, and trait ontology related to blast disease resistance were identified using the RAP-DB (https://rapdb.dna.affrc.go.jp/search/) and Oryzabase databases (https://shigen.nig.ac.jp/rice/oryzabase/download/gene). Functional annotation (gene ontology (GO) and relationships between query genes based on GO terms, gene functions, etc.) was performed using the shinyGO v0.741 (http://bioinformatics.sdstate.edu/go74/) online tool^54^. The Rice Expression Profile Database (RiceXPRO), a repository of 40 K microarray-based expression profiles, was utilized in our study because it is based on gene models in the RAP-DB. Furthermore, the RXP_3001 dataset was chosen for analysis. This dataset included the expression profiles of the genes in the whole leaf of *Oryza sativa* cv. Nipponbare inoculated with *M. oryzae* strains P91-15B and Kyu-077A in relation to water (H_2_O) treatment. In our study, the expression profiles of 199 candidate genes identified within 29 MQTLs were extracted from the RXP_3001 dataset in the RiceXPro Version 3 online tool/database (https://ricexpro.dna.affrc.go.jp/).

### Phenotyping of rice lines for blast disease response

Screening for rice blast disease response was carried out in a standard uniform blast nursery (UBN) described previously^55^. Briefly, each test entry (20–30 plants/test entry) was raised in 1 m long rows on raised nursery beds with a 10 cm row spacing. One row of HR-12 (susceptible check) was sown after every 5 test entries and also along the boundaries to ensure adequate inoculum build-up and disease spread. Entries were screened in a UBN using augmented design during Kharif 2022 and 2023. In our experiment, we used a highly virulent strain MoK19-18 (NCBI GenBank Accession No. MT757287) isolated from a popular mega rice cultivar BPT-5204 following the spore drop technique^56^. The mycelium and conidia were brushed into distilled water and filtered through muslin cloth. The spore concentration was adjusted approximately to 1 × 10^5^/mL^−1^. The spore suspension containing Tween-20 (0.2%) was sprayed uniformly over all the entries after 25 days post-sowing (2–3 leaf stage) during the late evening hours, and plants were covered with a plastic tarpaulin overnight to create humid condition (> 90%). Further, water was sprayed 4–6 times a day for seven days to maintain high humidity and facilitate the disease development. Parallelly, blast disease pressure in the screening plots was augmented as described previously^57^. Briefly, blast disease-infected leaf samples were collected from naturally infected diseased plants under field conditions and were incubated overnight in a polythene cover to promote sporulation. Later, *M. oryzae* spores were harvested, and the spore suspension was sprayed uniformly on all entries. This process was repeated continuously for 3–4 days in an interval of 3 days. This approach specifically helpful for slow blast phenotyping, where the disease occurrence is slow^57^. Disease scoring was done after the susceptible check reached the highest score (9), approximately 7 days post inoculation, and scoring was repeated six times in 5-day intervals to avoid the escape of slow-blasting genotypes. A 0–9 scale devised by IRRI, Philippines^58^, was used to record the disease reaction.

### Orthology of genes in *O. sativa* indica, Z. *mays* and *A. thaliana*

The *O. sativa* indica, maize, and Arabidopsis orthologs of genes within the MQTL region were identified using the ‘Biomart’ tool of the *Ensembl* Plants database^53^. The genes within MQTLs that were orthologous to the genes in the *O. sativa* indica, maize, and Arabidopsis genomes were visualized in a Circos plot using R software. The orthology of genes related to rice blast resistance was obtained through the g:Profiler online tool^59^.

### Supplementary Information

Supplementary Information.

## Data Availability

Data is provided within the manuscript and its supplementary information files.

## References

[1] Devanna BN (2022). Understanding the dynamics of blast resistance in rice-*Magnaporthe oryzae* interactions. J. Fungi.

[2] Marcel S, Sawers R, Oakeley E, Angliker H, Paszkowski U (2010). Tissue-adapted invasion strategies of the rice blast fungus *Magnaporthe oryzae*. Plant Cell.

[3] Asibi AE, Chai Q, Coulter JA (2019). Rice blast: A disease with implications for global food security. Agronomy.

[4] Tan J, Zhao H, Li J, Gong Y, Li X (2023). The devastating rice blast airborne pathogen *Magnaporthe oryzae*—A review on genes studied with mutant analysis. Pathogens.

[5] Murunde R, Ringo G, Robinson-Boyer L, Xu X (2022). Effective biocontrol of rice blast through dipping transplants and foliar applications. Agronomy.

[6] Zhang R-S (2022). Iturins produced by *Bacillus velezensis* Jt84 play a key role in the biocontrol of rice blast disease. Biol. Control.

[7] Korinsak S (2022). Identification of broad-spectrum resistance QTLs against rice blast fungus and their application in different rice genetic backgrounds. J. Genet..

[8] Kou Y, Wang S (2010). Broad-spectrum and durability: Understanding of quantitative disease resistance. Curr. Opin. Plant Biol..

[9] Devi SJSR (2020). Identification and characterization of a large effect QTL from *Oryza glumaepatula* revealed Pi68(t) as putative candidate gene for rice blast resistance. Rice.

[10] Jiang H (2020). Identification of blast resistance QTLs based on two advanced backcross populations in rice. Rice.

[11] Chaipanya C (2017). Dissection of broad-spectrum resistance of the Thai rice variety Jao Hom Nin conferred by two resistance genes against rice blast. Rice.

[12] Aglawe SB (2017). Identification of novel QTLs conferring field resistance for rice leaf and neck blast from a unique landrace of India. Gene Rep..

[13] Aloryi KD (2022). A meta-quantitative trait loci analysis identified consensus genomic regions and candidate genes associated with grain yield in rice. Front. Plant Sci..

[14] Liao CY, Wu P, Hu B, Yi KK (2001). Effects of genetic background and environment on QTLs and epistasis for rice (*Oryza sativa* L.) panicle number. Theor. Appl. Genet..

[15] Anilkumar C (2022). Understanding complex genetic architecture of rice grain weight through QTL-meta analysis and candidate gene identification. Sci. Rep..

[16] Veyrieras J-B, Goffinet B, Charcosset A (2007). MetaQTL: A package of new computational methods for the meta-analysis of QTL mapping experiments. BMC Bioinform..

[17] Arcade A (2004). BioMercator: integrating genetic maps and QTL toward discovery of candidate genes. Bioinformatics.

[18] Goffinet B, Gerber S (2000). Quantitative trait loci: A meta-analysis. Genetics.

[19] Zhang X, Shabala S, Koutoulis A, Shabala L, Zhou M (2017). Meta-analysis of major QTL for abiotic stress tolerance in barley and implications for barley breeding. Planta.

[20] Wang L (2019). Large-scale identification and functional analysis of *NLR* genes in blast resistance in the Tetep rice genome sequence. Proc. Natl. Acad. Sci. U.S.A..

[21] Xu X (2014). Rice blast resistance gene Pikahei-1(t), a member of a resistance gene cluster on chromosome 4, encodes a nucleotide-binding site and leucine-rich repeat protein. Mol Breed..

[22] Lin F (2007). The blast resistance gene Pi37 encodes a nucleotide binding site–leucine-rich repeat protein and is a member of a resistance gene cluster on rice chromosome 1. Genetics.

[23] Susan A (2019). Molecular identification of blast resistance genes in rice landraces from northeastern India. Plant Pathol..

[24] Sato Y (2013). RiceXPro version 3.0: Expanding the informatics resource for rice transcriptome. Nucleic Acids Res..

[25] Dong L (2020). Identification and fine mapping of Pi69(t), a new gene conferring broad-spectrum resistance against *Magnaporthe oryzae* from *Oryza glaberrima* Steud. Front. Plant Sci..

[26] Fang N (2019). Fine mapping of a panicle blast resistance gene Pb-bd1 in Japonica landrace Bodao and its application in rice breeding. Rice.

[27] Kumar IS, Nadarajah K (2020). A meta-analysis of quantitative trait loci associated with multiple disease resistance in rice (*Oryza sativa* L.). Plants.

[28] Khahani B, Tavakol E, Shariati V, Rossini L (2021). Meta-QTL and ortho-MQTL analyses identified genomic regions controlling rice yield, yield-related traits and root architecture under water deficit conditions. Sci. Rep..

[29] Li W (2009). The *Magnaporthe oryzae* avirulence gene *AvrPiz-t* encodes a predicted secreted protein that triggers the immunity in rice mediated by the blast resistance gene *Piz-t*. MPMI.

[30] Qu S (2006). The broad-spectrum blast resistance gene Pi9 encodes a nucleotide-binding site–leucine-rich repeat protein and is a member of a multigene family in rice. Genetics.

[31] He X (2012). Identification of the novel recessive gene pi55(t) conferring resistance to Magnaporthe oryzae. Sci. China Life Sci..

[32] Nguyet NTM (2020). Diversity and distribution of rice blast (*Pyricularia oryzae* Cavara) races in Vietnam. Plant Dis..

[33] Okuyama Y (2011). A multifaceted genomics approach allows the isolation of the rice *Pia* -blast resistance gene consisting of two adjacent NBS–LRR protein genes. Plant J..

[34] Azizi P (2016). Over-expression of the Pikh gene with a CaMV 35S promoter leads to improved blast disease (*Magnaporthe oryzae*) tolerance in rice. Front. Plant Sci..

[35] Inoue H (2013). Blast resistance of CC-NB-LRR protein Pb1 is mediated by WRKY45 through protein–protein interaction. Proc. Natl. Acad. Sci. U.S.A..

[36] Ashikawa I (2008). Two adjacent nucleotide-binding site–leucine-rich repeat class genes are required to confer Pikm-specific rice blast resistance. Genetics.

[37] Zhan C (2020). Selection of a subspecies-specific diterpene gene cluster implicated in rice disease resistance. Nat. Plants.

[38] Ballini E (2007). Modern elite rice varieties of the ‘Green Revolution’ have retained a large introgression from wild rice around the *Pi33* rice blast resistance locus. New Phytol..

[39] Boland A, Côté J, Barford D (2023). Structural biology of DOCK-family guanine nucleotide exchange factors. FEBS Lett..

[40] Chandran V (2019). miR396-OsGRF s module balances growth and rice blast disease-resistance. Front. Plant Sci..

[41] Helliwell EE, Wang Q, Yang Y (2013). Transgenic rice with inducible ethylene production exhibits broad-spectrum disease resistance to the fungal pathogens *M**agnaporthe oryzae* and *R**hizoctonia solani*. Plant Biotechnol. J..

[42] Wu Y (2019). Comprehensive evaluation of resistance effects of pyramiding lines with different broad-spectrum resistance genes against Magnaporthe oryzae in rice (*Oryza sativa* L.). Rice.

[43] Ballini E (2008). A genome-wide meta-analysis of rice blast resistance genes and quantitative trait loci provides new insights into partial and complete resistance. MPMI.

[44] Li W (2011). Rice RING protein OsBBI1 with E3 ligase activity confers broad-spectrum resistance against *Magnaporthe oryzae* by modifying the cell wall defense. Cell Res..

[45] Zeng L-R (2004). Spotted leaf11, a negative regulator of plant cell death and defense, encodes a U-box/armadillo repeat protein endowed with E3 ubiquitin ligase activity. Plant Cell.

[46] De la Concepcion JC (2021). The allelic rice immune receptor Pikh confers extended resistance to strains of the blast fungus through a single polymorphism in the effector binding interface. PLoS Pathog..

[47] Collard BCY, Mackill DJ (2008). Marker-assisted selection: an approach for precision plant breeding in the twenty-first century. Phil. Trans. R. Soc. B.

[48] Sosnowski O, Charcosset A, Joets J (2012). BioMercator V3: An upgrade of genetic map compilation and quantitative trait loci meta-analysis algorithms. Bioinformatics.

[49] Darvasi A, Soller M (1997). No title found. Behav. Genet..

[50] Gupta M (2023). Meta-QTL analysis for mining of candidate genes and constitutive gene network development for fungal disease resistance in maize (*Zea mays* L.). Crop J..

[51] Madhusudhan P (2022). Screening of rice genotypes for resistance against blast and bacterial leaf blight. Plant Dis. Res..

[52] Doyle JJ, Doyle JL (1987). A rapid DNA isolation procedure for small quantities of fresh leaf tissue. Phytochem. Bull..

[53] Kinsella RJ (2011). Ensembl BioMarts: A hub for data retrieval across taxonomic space. Database.

[54] Ge SX, Jung D, Yao R (2020). ShinyGO: A graphical gene-set enrichment tool for animals and plants. Bioinformatics.

[55] Kumar V (2014). Large scale germplasm screening for identification of novel rice blast resistance sources. Front. Plant Sci..

[56] Amoghavarsha C (2022). A simplified spore-drop technique for rapid isolation of rice blast pathogen *Magnaporthe oryzae* from the infected rice leaf. Oryza.

[57] Pramesh D (2023). Moderate disease resistance in rice cultivars enhances the bio-efficacy of fungicides against blast disease. Indian Phytopathol..

[58] Anonymous. International Network for Genetic Evaluation of Rice. *Standard evaluation system for rice*. IRRI, International Rice Research Institute (1996).

[59] Raudvere U (2019). g: Profiler: a web server for functional enrichment analysis and conversions of gene lists (2019 update). Nucleic Acids Res..

[60] Ahn S-N (2000). No title found. Euphytica.

[61] Ashkani S (2011). Analysis of simple sequence repeat markers linked with blast disease resistance genes in a segregating population of rice (*Oryza sativa*). Genet. Mol. Res..

[62] Ashkani S (2012). SSRs for marker-assisted selection for blast resistance in rice (*Oryza sativa* L.). Plant Mol. Biol. Rep..

[63] Ashkani S, Rafii MY, Rahim HA, Latif MA (2013). Genetic dissection of rice blast resistance by QTL mapping approach using an F3 population. Mol. Biol. Rep..

[64] Bagali PG, Hittalmani S, Shashidhar SY, Shashidhar HE, Tharreau D, Lebrun MH, Talbot NJ, Notteghem JL (2000). Identification of DNA markers linked to partial resistance for blast disease in rice across four locations. Advances in Rice Blast Research.

[65] Biradar H, Bhargavi MV, Sasalwad R, Parama R, Hittalmani S (2007). Identification of QTL associated with silicon and zinc content in rice (*Oryza sativa* L.) and their role in blast disease resistance. Indian J. Genet. Plant Breed..

[66] Chen H (2003). Comparative analyses of genomic locations and race specificities of loci for quantitative resistance to *Pyricularia grisea* in rice and barley. Proc. Natl. Acad. Sci. U.S.A..

[67] Cho Y-C (2008). QTLs identification and confirmation of field resistance to leaf blast in temperate japonica rice (*Oryza sativa* L.). J. Crop Sci. Biotechnol..

[68] Fang N (2016). QTL mapping of panicle blast resistance in japonica landrace Heikezijing and its application in rice breeding. Mol Breed..

[69] Fukuoka S, Okuno K (2001). QTL analysis and mapping of pi21, a recessive gene for field resistance to rice blast in Japanese upland rice. Theor. Appl. Genet..

[70] Lopez-Gerena, J. Mapping QTL controlling durable resis tance to rice blast in the variety Oryzica Llanos 5. (Ph. D. Thesis. Kansas State University, USA, 2006).

[71] Guo L (2016). Dissection of QTL alleles for blast resistance based on linkage and linkage disequilibrium mapping in japonica rice seedlings. Australas. Plant Pathol..

[72] He W (2017). Fine mapping of a new race-specific blast resistance gene, Pi-hk2, in Japonica Heikezijing from Taihu Region of China. Phytopathology.

[73] Hittalmani S, Srinivasachary, Bagali P, Shashidhar HE, Khush GS, Brar DS, Hardy B (2008). Identifying major genes and QTLs for field resistance to neck blast in rice. Advances in Rice Genetics.

[74] Hu K-M, Qiu D-Y, Shen X-L, Li X-H, Wang S-P (2008). Isolation and manipulation of quantitative trait loci for disease resistance in rice using a candidate gene approach. Mol. Plant.

[75] Huan J (2014). Identification of quantitative trait loci conferring blast resistance in Bodao, a japonica rice landrace. Genet. Mol. Res..

[76] Ishihara T (2014). Quantitative trait locus analysis of resistance to panicle blast in the rice cultivar Miyazakimochi. Rice.

[77] Jia Y, Liu G (2011). Mapping quantitative trait loci for resistance to rice blast. Phytopathology.

[78] Xing J (2015). Confirming and identifying new loci for rice blast disease resistance using *Magnaporthe oryzae* field isolates in the US. Crop Sci..

[79] Kongprakhon P (2010). Four QTL in rice associated with broad spectrum resistance to blast isolates from rice and barley. J. Phytopathol..

[80] Li Y, Wu C, Xing Y, Chen H, He Y (2008). Dynamic QTL analysis for rice blast resistance under natural infection conditions. Aust. J. Crop Sci..

[81] Lo K-L (2022). Two genomic regions of a sodium azide induced rice mutant confer broad-spectrum and durable resistance to blast disease. Rice.

[82] Mandal L, Verma S, Kotasthane A, Verulkar S (2017). Identification of quantitative trait loci for leaf blast resistance of rice (*Oryza sativa* L.). BJI.

[83] Miyamoto M, Yano M, Hirasawa H (2001). Mapping of quantitative trait loci conferring blast field resistance in the Japanese upland rice variety Kahei. Breed. Sci..

[84] Mizobuchi R (2014). Mapping of a QTL for field resistance to blast (*Pyricularia oryzae* Cavara) in Ingngoppor-tinawon, a rice (*Oryza sativa* L.) landrace from the Philippines. JARQ.

[85] Nagaoka I (2017). Quantitative trait loci analysis of blast resistance in *Oryza sativa* L. ‘Hokuriku 193’. Breed. Sci..

[86] Noenplab A (2005). QTL Mapping for Leaf and Neck Blast Resistance in Khao Dawk Mali105 and Jao Hom Nin Recombinant Inbred Lines.

[87] Lestari P (2011). Mapping quantitative trait loci conferring blast resistance in upland indica rice (*Oryza sativa* L.). J. Crop Sci. Biotechnol..

[88] Rahim HA (2012). Identification of quantitative trait loci for blast resistance in BC2F3 and BC2F5 advanced backcross families of rice. Genet. Mol. Res..

[89] Rahman L, Khanam S, Roh J-H, Koh H-J (2011). Mapping of QTLs involved in resistence to rice blast (*Magnaporthe grisea*) using *Oryza minuta* introgression lines. Czech J. Genet. Plant Breed..

[90] Sabouri H, Sabouri A, Jafarzadeh MR, Mollashahi M (2011). Detection of QTLs controlling field blast resistance in rice (*Oryza sative* L.). Plant Omics.

[91] Sallaud C (2003). Identification of five new blast resistance genes in the highly blast-resistant rice variety IR64 using a QTL mapping strategy. Theor. Appl. Genet..

[92] Sato H (2006). Mapping QTLs for field resistance to rice blast in the Japanese upland rice variety Norin 12. Breed. Sci..

[93] Shi X (2010). Identification of the quantitative trait loci in japonica rice landrace Heikezijing responsible for broad-spectrum resistance to rice blast. Phytopathology.

[94] Sirithunya P (2002). Quantitative trait loci associated with leaf and neck blast resistance in recombinant inbred line population of rice (*Oryza sativa*). DNA Res..

[95] Sobrizal S (2010). Identification of a major quantitative trait locus conferring rice blast resistance using recombinant inbred lines. Indones. J. Agric. Sci..

[96] Sreewongchai T (2010). Development of elite indica rice lines with wide spectrum of resistance to Thai blast isolates by pyramiding multiple resistance QTLs. Plant Breed..

[97] Tabien R (2002). Mapping QTLs for field resistance to the rice blast pathogen and evaluating their individual and combined utility in improved varieties. Theor. Appl. Genet..

[98] Talukder ZI, McDonald AJS, Price AH (2005). Loci controlling partial resistance to rice blast do not show marked QTL×environment interaction when plant nitrogen status alters disease severity. New Phytol..

[99] Urso S (2016). Genetic analysis of durable resistance to *Magnaporthe oryzae* in the rice accession Gigante Vercelli identified two blast resistance loci. Mol. Genet. Genomics.

[100] Wang Y (2012). Molecular mapping of the blast resistance genes Pi2-1 and Pi51(t) in the durably resistant rice ‘Tianjingyeshengdao’. Phytopathology.

[101] Wongsaprom C (2010). Two introgressed quantitative trait loci confer a broad-spectrum resistance to blast disease in the genetic background of the cultivar RD6 a Thai glutinous jasmine rice. Field Crops Res..

[102] Xiao J, Li J, Yuan L, Tanksley SD (1995). Dominance is the major genetic basis of heterosis in rice as revealed by qtl analysis using molecular markers. Genetics.

[103] Xu J (2004). Analysis of rice blast resistance genes by QTL mapping. Chin. Sci. Bull..

[104] Yang H (2013). Molecular mapping of four blast resistance genes using recombinant inbred lines of 93–11 and nipponbare. J. Plant Biol..

[105] Zenbayashi K, Ashizawa T, Tani T, Koizumi S (2002). Mapping of the QTL (quantitative trait locus) conferring partial resistance to leaf blast in rice cultivar Chubu 32. Theor. Appl. Genet..

