# Supplementary material for: Refinement of rice blast disease resistance QTLs and gene networks through meta-QTL analysis
Source: Sci Rep. 2024 Jul 16;14:16458. doi: 10.1038/s41598-024-64142-0 (PMC11252161; doi:10.1038/s41598-024-64142-0)
Supplement: Supplementary file 1 — Supplementary Information. [file 41598_2024_64142_MOESM1_ESM.docx]

**Table S1** Chromosome position, interval, number of QTL and genes in the Meta-QTL regions associated with rice blast disease resistance

| **QTL** | **Chr** | **Position**  **(cM)** | **Peak marker** | **Weight** | **Distance** | **CI (95%)** | **UCI**  **(95%)** | **No. of genes/ interval** | **No. of original QTL** | **Left flanking marker** | **Right flanking marker** | **Start (bp)** | **End (bp)** | **Interval (Mb)** |
| --- | --- | --- | --- | --- | --- | --- | --- | --- | --- | --- | --- | --- | --- | --- |
| M-QTL1.1 | 1 | 0.51 | RM10007 | 0.05 | 2.87 | 0.52 | 0.52 | 26 | 3 | RM10004 | RM495 | 81418 | 216000 | 0.13 |
| M-QTL1.2 | 1 | 22.7 | RM10338 | 0.07 | 3.16 | 4.98 | 4.98 | 161 | 4 | RM10296 | RM10379 | 5063913 | 6379069 | 1.32 |
| M-QTL1.3 | 1 | 52.93 | RM10820 | 0.1 | 2.15 | 7.62 | 7.62 | 147 | 6 | RM493 | RM10864 | 12264091 | 14236113 | 1.97 |
| M-QTL1.4 | 1 | 72.37 | RM11015 | 0.06 | 3.04 | 6.17 | 6.17 | 130 | 3 | RM10989 | RM11046 | 17288918 | 18920107 | 1.63 |
| M-QTL1.5 | 1 | 110.94 | RM7566 | 0.14 | 1.13 | 7.67 | 10.55 | 275 | 8 | RM11447 | RM11553 | 26756315 | 28739851 | 1.98 |
| M-QTL1.6 | 1 | 127.66 | RM3709 | 0.23 | 1.3 | 6.43 | 12.27 | 8 | 15 | RM7643 | RM11731 | 31132390 | 32749013 | 1.62 |
| M-QTL1.7 | 1 | 144.42 | RM11860 | 0.29 | 1.94 | 4.72 | 11.31 | 191 | 16 | RM11838 | RM315 | 35484517 | 36728458 | 1.24 |
| M-QTL1.8 | 1 | 164.51 | RM5536 | 0.06 | - | 3.19 | 10.24 | 143 | 4 | RM12151 | RM12183 | 40742759 | 41562458 | 0.82 |
| M-QTL2.1 | 2 | 34.29 | RM12852 | 0.25 | 1.85 | 7.06 | 8.39 | 153 | 8 | RM12805 | RM12918 | 7689430 | 9459499 | 1.77 |
| M-QTL2.2 | 2 | 61.65 | RM13157 | 0.07 | 2.11 | 17.9 | 18.28 | 268 | 3 | RM13093 | RM13249 | 13177584 | 17654823 | 4.48 |
| M-QTL2.3 | 2 | 93.26 | RZ318 | 0.08 | 1.3 | 12.94 | 14.2 | 416 | 2 | RM13457 | RM13623 | 21644595 | 24963721 | 3.32 |
| M-QTL2.4 | 2 | 109.08 | RM13746 | 0.11 | 1.28 | 3.89 | 3.92 | 129 | 2 | RM13723 | RM13771 | 26735357 | 27762708 | 1.03 |
| M-QTL2.5 | 2 | 125.46 | RM13963 | 0.42 | 1.23 | 7.55 | 7.59 | 289 | 17 | RM13927 | RM425 | 30402786 | 32322336 | 1.92 |
| M-QTL2.6 | 2 | 139.43 | RM14158 | 0.07 | - | 0.15 | 0.16 | 11 | 2 | RM14156 | RM14164 | 34821219 | 34890307 | 0.07 |
| M-QTL3.1 | 3 | 47.32 | RM14876 | 0.19 | 0.82 | 1.46 | 5.6 | 47 | 10 | RM14860 | RM14886 | 11624423 | 12016970 | 0.39 |
| M-QTL3.2 | 3 | 56.92 | RM15022 | 0.18 | 2.43 | 0.74 | 0.74 | 27 | 8 | RM15011 | RM6676 | 14135453 | 14327686 | 0.19 |
| M-QTL3.3 | 3 | 81.98 | RM15325 | 0.09 | 2.43 | 1.2 | 1.2 | 27 | 4 | RM6392 | RM15328 | 20258449 | 20648095 | 0.39 |
| M-QTL3.4 | 3 | 108.76 | RM6053 | 0.4 | 2.47 | 4.17 | 6.35 | 124 | 19 | RM3049 | RM15740 | 26613246 | 27730287 | 1.12 |
| M-QTL3.5 | 3 | 132.53 | RM16065 | 0.14 | - | 1 | 1.02 | 49 | 5 | RM16057 | RM16074 | 32996812 | 33275676 | 0.28 |
| M-QTL4.1 | 4 | 22.71 | RM16473 | 0.07 | 1.34 | 13.2 | 13.21 | 219 | 2 | RM16414 | RM16516 | 4043672 | 7386044 | 3.34 |
| M-QTL4.2 | 4 | 39.82 | RM16574 | 0.08 | 1.81 | 1.8 | 1.8 | 12 | 2 | RM16568 | RM16576 | 9727610 | 10368520 | 0.64 |
| M-QTL4.3 | 4 | 68.26 | RM16761 | 0.23 | 2.2 | 5.36 | 5.4 | 75 | 8 | RM16734 | RM16777 | 16370162 | 17785011 | 1.41 |
| M-QTL4.4 | 4 | 102.76 | RM17231 | 0.12 | 1.06 | 7.2 | 7.48 | 295 | 3 | RM17178 | RM17282 | 24829833 | 26685400 | 1.86 |
| M-QTL4.5 | 4 | 115.89 | RM17376 | 0.38 | 2.27 | 1.67 | 1.91 | 53 | 11 | RM17375 | RM17381 | 28787059 | 29217144 | 0.43 |
| M-QTL4.6 | 4 | 136.1 | RM3466 | 0.12 | - | 2.39 | 3.14 | 104 | 3 | RM17599 | RM17632 | 33767624 | 34382879 | 0.62 |
| M-QTL5.1 | 5 | 12.94 | RM17935 | 0.15 | 2.34 | 9.22 | 9.28 | 282 | 3 | RM17860 | RM17979 | 2077323 | 4430461 | 2.35 |
| M-QTL5.2 | 5 | 40.59 | RM18223 | 0.37 | 4.23 | 5.09 | 12.84 | 26 | 10 | RM18208 | RM18250 | 9522150 | 10837378 | 1.32 |
| M-QTL5.3 | 5 | 95.28 | RM18899 | 0.33 | 0.89 | 5.82 | 11.67 | 201 | 9 | RM18858 | RM18932 | 23093974 | 24559747 | 1.47 |
| M-QTL5.4 | 5 | 104.26 | RM19017 | 0.15 | - | 1.32 | 1.35 | 62 | 2 | RM538 | RM19025 | 25888255 | 26257396 | 0.37 |
| M-QTL6.1 | 6 | 4.93 | RM19301 | 0.06 | 2.93 | 7.64 | 8.2 | 306 | 2 | RM19242 | RM19366 | 396243 | 2316173 | 1.92 |
| M-QTL6.2 | 6 | 16.45 | RM19485 | 0.03 | 3.54 | 9.52 | 10.48 | 363 | 1 | RM19419 | RM253 | 2995026 | 5437340 | 2.44 |
| M-QTL6.3 | 6 | 38.58 | RM19801 | 0.16 | 2.94 | 4.53 | 4.53 | 68 | 5 | RM6162 | RM19820 | 9210145 | 10416038 | 1.21 |
| M-QTL6.4 | 6 | 62.73 | RM20058 | 0.19 | 2.13 | 2.97 | 3.71 | 46 | 7 | RM20018 | RM20065 | 15385699 | 16213923 | 0.83 |
| M-QTL6.5 | 6 | 83.33 | RM20241 | 0.17 | 1.45 | 8.87 | 8.87 | 204 | 6 | RM20181 | RM20285 | 19838168 | 22074088 | 2.24 |
| M-QTL6.6 | 6 | 99 | CD0544 | 0.13 | 1.08 | 7.05 | 7.05 | 183 | 3 | RM20382 | RM20475 | 24011349 | 25763397 | 1.75 |
| M-QTL6.7 | 6 | 110.28 | RM20593 | 0.27 | - | 0.04 | 0.04 | 6 | 11 | RM20592 | RM20594 | 27664120 | 27704549 | 0.04 |
| M-QTL7.1 | 7 | 19.46 | RM21130 | 0.05 | 2.08 | 1.7 | 1.7 | 54 | 1 | RM21106 | RM21143 | 4702756 | 5134711 | 0.43 |
| M-QTL7.2 | 7 | 28.62 | RM21255 | 0.15 | 2.51 | 0.11 | 0.11 | 15 | 3 | RM1253 | RM21259 | 7001046 | 7223574 | 0.22 |
| M-QTL7.3 | 7 | 52.7 | RM21435 | 0.2 | 1.21 | 7.3 | 7.3 | 89 | 5 | RM21419 | RM21452 | 12087002 | 14204785 | 2.12 |
| M-QTL7.4 | 7 | 67.27 | RM21558 | 0.21 | 2.27 | 7.41 | 7.44 | 114 | 4 | RM5875 | RM21606 | 15945284 | 17827291 | 1.88 |
| M-QTL7.5 | 7 | 94.67 | RM21887 | 0.21 | 1.45 | 7.46 | 7.49 | 278 | 5 | RM21844 | RM21932 | 22744493 | 24654001 | 1.91 |
| M-QTL7.6 | 7 | 111.6 | RM22100 | 0.18 | - | 6.92 | 7.93 | 262 | 4 | RM22065 | RM22142 | 27028300 | 28836061 | 1.81 |
| M-QTL8.1 | 8 | 28.74 | RM22627 | 0.14 | 2.32 | 6.81 | 22.96 | 94 | 4 | RM22595 | RM22665 | 6310374 | 8130860 | 1.82 |
| M-QTL8.2 | 8 | 54.9 | RM22866 | 0.23 | 1.53 | 5.79 | 5.81 | 56 | 7 | RM22849 | RM22887 | 13026809 | 14481243 | 1.45 |
| M-QTL8.3 | 8 | 72.01 | RM23008 | 0.1 | 1.73 | 10.24 | 9.98 | 186 | 2 | RM22968 | RM23062 | 16703161 | 19316338 | 2.61 |
| M-QTL8.4 | 8 | 87.74 | RM23224 | 0.35 | 1.88 | 0.6 | 5.98 | 18 | 10 | RM23217 | RM23226 | 21870671 | 22059276 | 0.19 |
| M-QTL8.5 | 8 | 102.28 | RM23442 | 0.19 | - | 3.08 | 9.06 | 114 | 5 | RM23412 | RM23462 | 25212656 | 25991608 | 0.78 |
| M-QTL9.1 | 9 | 25.24 | RM23880 | 0.19 | 1.36 | 10.92 | 12.39 | 141 | 6 | RM23820 | RM23942 | 5036985 | 7775910 | 2.74 |
| M-QTL9.2 | 9 | 38.49 | RM24030 | 0.16 | 1.79 | 1 | 1 | 26 | 3 | RM24019 | RM24031 | 9593599 | 9853288 | 0.26 |
| M-QTL9.3 | 9 | 54.16 | RM24236 | 0.33 | 3.21 | 3.59 | 4.03 | 62 | 8 | RM24220 | RM24260 | 13196284 | 14097479 | 0.90 |
| M-QTL9.4 | 9 | 83.38 | RM24721 | 0.25 | 0.85 | 1.96 | 1.96 | 74 | 7 | RM1553 | RM24745 | 20702581 | 21187014 | 0.48 |
| M-QTL9.5 | 9 | 89.87 | RM24844 | 0.06 | - | 0.31 | 0.34 | 11 | 1 | RM24838 | RM24846 | 22525258 | 22609290 | 0.08 |
| M-QTL10.1 | 10 | 16.42 | RM25058 | 0.16 | 2.06 | 6.69 | 27.75 | 115 | 3 | RM25001 | RM25090 | 3233585 | 4983041 | 1.75 |
| M-QTL10.2 | 10 | 41.63 | RM25262 | 0.62 | 3.48 | 10.83 | 17.11 | 196 | 11 | RM25212 | RM25312 | 9083802 | 11803333 | 2.72 |
| M-QTL10.3 | 10 | 85.01 | RM25833 | 0.22 | - | 3.67 | 21.03 | 143 | 4 | RM25806 | RM25862 | 20819173 | 21741428 | 0.92 |
| M-QTL11.1 | 11 | 19.29 | RM26220 | 0.11 | 1.89 | 1.24 | 3.34 | 189 | 7 | RM26220 | RM3625 | 4869611 | 6591206 | 1.72 |
| M-QTL11.2 | 11 | 31.7 | RM26352 | 0.1 | 0.57 | 1.14 | 3.97 | 34 | 9 | RM26352 | RM26367 | 7840908 | 8156135 | 0.32 |
| M-QTL11.3 | 11 | 36.08 | RM7283 | 0.04 | 2.22 | 2.57 | 2.58 | 52 | 2 | RM26393 | RM26433 | 8757709 | 9515519 | 0.76 |
| M-QTL11.4 | 11 | 54.62 | RM26602 | 0.06 | 1.55 | 10.29 | 10.22 | 117 | 4 | RM26556 | RM26653 | 12389469 | 15076115 | 2.69 |
| M-QTL11.5 | 11 | 68.31 | RM26768 | 0.11 | 0.75 | 5.06 | 6.2 | 79 | 3 | RM26724 | RM26797 | 16471342 | 17781259 | 1.31 |
| M-QTL11.6 | 11 | 74.56 | RM26839 | 0.19 | 1.75 | 2.61 | 2.83 | 42 | 18 | RM26821 | RM26860 | 18354355 | 19037896 | 0.68 |
| M-QTL11.7 | 11 | 87.64 | RM27016 | 0.06 | 1.43 | 2.96 | 2.98 | 97 | 3 | RM27010 | RM27036 | 21905323 | 22869558 | 0.96 |
| M-QTL11.8 | 11 | 96.78 | RM4112 | 0.2 | 1.63 | 1.95 | 1.96 | 104 | 15 | RM27128 | RM27211 | 24036953 | 25286896 | 1.25 |
| M-QTL11.9 | 11 | 106.7 | RM27285 | 0.13 | - | 1.09 | 1.1 | 113 | 10 | RM27275 | RM27352 | 26600144 | 28002104 | 1.40 |
| M-QTL12.1 | 12 | 14.16 | RM27607 | 0.1 | 0.91 | 5 | 4.97 | 165 | 5 | RM27548 | RM27655 | 2966021 | 4232841 | 1.27 |
| M-QTL12.2 | 12 | 21.67 | RM27723 | 0.06 | 3.03 | 6.16 | 6.32 | 107 | 2 | RM27686 | RM27750 | 4692761 | 6261104 | 1.57 |
| M-QTL12.3 | 12 | 55.68 | RM28045 | 0.33 | 1.03 | 7.54 | 7.76 | 85 | 17 | RM28003 | RM28069 | 13032073 | 14937946 | 1.91 |
| M-QTL12.4 | 12 | 68.12 | RM28149 | 0.12 | 1.26 | 1.98 | 2.01 | 10 | 3 | RM28135 | RM28154 | 16846449 | 17342025 | 0.50 |
| M-QTL12.5 | 12 | 81.6 | Bph10 | 0.14 | 0.86 | 6.78 | 8.11 | 103 | 8 | RM28285 | RM28370 | 19604577 | 21301135 | 1.70 |
| M-QTL12.6 | 12 | 89.76 | RM28428 | 0.11 | 0.98 | 5.44 | 6.27 | 126 | 3 | RM3326 | RM28483 | 21809349 | 23174920 | 1.37 |
| M-QTL12.7 | 12 | 98.6 | RM28598 | 0.15 | - | 3.3 | 5.5 | 76 | 6 | RM28574 | RM28637 | 24292314 | 25133847 | 0.84 |

**Table S2** Characterised resistance (R) gene analogues (RGA) candidates for blast resistance within the identified M-QTL

| **M-QTL** | **Gene stable ID** | **Chr** | **Gene description** | **Gene name** |
| --- | --- | --- | --- | --- |
| M-QTL1.2 | Os01g0200600 | 1 | Similar to Avr9/Cf-9 rapidly elicited protein 111A | OsERF#039 |
| M-QTL1.5 | Os01g0693400 | 1 | Pathogenesis-related transcriptional factor and ERF, DNA-binding domain containing protein | AP2/EREBP#127 |
| M-QTL1.5 | Os01g0694100 | 1 | Leucine-rich repeat domain containing protein | LRR-RLK |
| M-QTL1.5 | Os01g0669100 | 1 | S-domain receptor-like kinase, OsPR1b-interacting factor, Resistance protein, Grain yield, Disease resistance | OsLSK1 |
| M-QTL1.5 | Os01g0665200 | 1 | Similar to Blast and wounding induced mitogen-activated protein kinase | OsMPK20-4 |
| M-QTL1.5 | Os01g0678500 | 1 | Voltage-gated Ca2+ channel protein, Elicitor-induced defense reponses, Hypersensitive cell death, Activation of MAPK cascade | TPC1 |
| M-QTL1.7 | Os01g0841700 | 1 | Similar to Isoform ERG1b of Elicitor-responsive protein 1 | Rpp17 |
| M-QTL2.1 | Os02g0236100 | 2 | Leucine-rich repeat receptor-like kinase, Specification of root outer cell layer | Docs1 |
| M-QTL2.1 | Os02g0262800 | 2 | Similar to NBS-LRR protein (Fragment) | rNBS41 |
| M-QTL2.1 | Os02g0261100 | 2 | Ubiquitin-conjugating enzyme E2, Elicitor-induced defense response | UBC5B |
| M-QTL2.3 | Os02g0593600 | 2 | Leucine-rich repeat, typical subtype containing protein | OsIRL2 |
| M-QTL2.5 | Os02g0759700 | 2 | Leucine-rich repeat 2 containing protein | Os_F0767 |
| M-QTL4.4 | Os04g0529100 | 4 | Pathogenesis-related transcriptional factor and ERF domain containing protein | **OsERF#045** |
| M-QTL4.4 | Os04g0505700 | 4 | Leucine-rich repeat, cysteine-containing subtype containing protein | OsFbox227 |
| M-QTL5.3 | Os05g0486100 | 5 | Leucine-rich repeat receptor-like kinase, Mn<sup>2+</sup>/Mg<sup>2+</sup>-dependent serine/threonine (Ser/Thr) kinase, Ca<sup>2+</sup>-independent Ser/Thr kinase, Negative regulation of polar auxin transport, Root development | OsRPK1 |
| M-QTL5.3 | Os05g0492200 | 5 | NB-ARC domain containing protein | **YR1** |
| M-QTL5.3 | Os05g0492600 | 5 | Similar to NBS-LRR type resistance protein (Fragment) | **YR48** |
| M-QTL6.2 | Os06g0181700 | 6 | Pathogenesis-related transcriptional factor and ERF domain containing protein | **OsWR4** |
| M-QTL6.3 | Os06g0286500 | 6 | Similar to NBS-LRR disease resistance protein homologue | **Nbs1-Pi9** |
| M-QTL6.5 | Os06g0551400 | 6 | Ras-related small GTP-binding protein, Regulation of vesicular trafficking from trans-Golgi network to plasma membrane or vacuole, Jasmonic acid (JA)-mediated defense signaling | OsRab11 |
| M-QTL6.6 | Os06g0605900 | 6 | Leucine-rich repeat, cysteine-containing subtype containing protein | OsFbox316 |
| M-QTL7.2 | Os07g0227600 | 7 | Pathogenesis-related transcriptional factor/ERF, DNA-binding domain containing protein | **OsERF#057** |
| M-QTL8.1 | Os08g0224000 | 8 | NB-ARC domain containing protein | **OsOSC12** |
| M-QTL9.1 | Os09g0286600 | 9 | Pathogenesis-related transcriptional factor and ERF domain containing protein | **Sub1C** |
| M-QTL9.4 | Os09g0533600 | 9 | Similar to Avr9/Cf-9 induced kinase 1 | OsRLCK278 |
| M-QTL10.1 | Os10g0154000 | 10 | Membrane-trafficking protein, Intracellular SNARE protein, Resistance to rice blast disease | OsVAMP714 |
| M-QTL10.3 | Os10g0548700 | 10 | Receptor-like cytoplasmic kinase, Various stress responses, Regulation of resistance to bacterial leaf streak (BLS) | RLCK306 |
| MQTL11.1 | Os11g0224900 | 11 | Similar to NBS-LRR protein (Fragment). (Os11t0224900-00) | Os11gRGA3, RGA3 |
| MQTL11.1 | Os11g0225100 | 11 | Nucleotide binding site-leucine rich repeats (NBS-LRRs) protein, Resistance to the blast fungus, (Nippponbare: susceptible to the blast fungus carrying the AVR-Pia) (Os11t0225100-01) | **RGA4/ Pia** |
| MQTL11.1 | Os11g0225300 | 11 | Nucleotide binding site-leucine rich repeats (NBS-LRRs) protein, Resistance protein, Resistance to the blast fungus, (Nippponbare: susceptible to the blast fungus carrying the AVR-Pia) (Os11t0225300-01);Hypothetical conserved gene. (Os11t0225300-02) | **RGA5/Pia** |
| MQTL11.1 | Os11g0213500 | 11 | Pentatricopeptide repeat protein, Mitochondrial nad7 transcript editing, Seed development (Os11t0213500-01) | SMK1 |
| MQTL11.3 | Os11g0264200 | 11 | Cyclin-like F-box domain containing protein. (Os11t0264200-01) | OsFbox598, Os_F0672, OsFBX480, FBX480 |
| MQTL11.7 | Os11g0598500 | 11 | NB-ARC domain containing protein. (Os11t0598500-00);Atypical CC-NBS-LRR protein (St No.1), Atypical R protein-like protein, Pb1-like protein, Panicle blast-resistance (Nipponbare: blast-susceptible) (Os11t0598500-01) | **PB1** |
| MQTL11.7 | Os11g0590700 | 11 | NB-ARC domain containing protein. (Os11t0590700-01) | RPP13-like protein 3 |
| M-QTL11.7 | Os11g0588600 | 11 | NB-ARC domain containing protein | **Xa39** |
| MQTL11.8 | Os11g0639100 | 11 | NBS-LRR protein, Blast resistance, Broad-spectrum resistance against *Magnaporthe oryzae* (Nipponbare: susceptible) (Os11t0639100-01) | **PIKH** |
| MQTL11.8 | Os11g0633500 | 11 | Similar to NBS-LRR disease resistance protein homologue (Fragment). (Os11t0633500-01) | RPP8 |
| MQTL11.9 | Os11g0673900 | 11 | Similar to NB-ARC domain containing protein. (Os11t0673900-00); Disease resistance protein-RP13 | DRP-RP13 |
| MQTL11.9 | Os11g0689100 | 11 | Coiled-coil NBS-LRR protein, Blast resistance (Os11t0689100-01); Similar to NBS-LRR class disease resistance protein. (Os11t0689100-02) | **PIKM, PIK** |
| MQTL11.9 | Os11g0688832 | 11 | Coiled-coil NBS-LRR protein, Blast resistance, Resistance to bacterial blight (Os11t0688832-01) | **PIKM, PIK** |
| MQTL11.9 | Os11g0683700 | 11 | Pectin lyase fold domain containing protein. (Os11t0683700-01) | PME33 |
| MQTL11.9 | Os11g0683800 | 11 | Pectin lyase fold/virulence factor domain containing protein. (Os11t0683800-01);Pectin lyase fold/virulence factor domain containing protein. (Os11t0683800-02) | PME34 |
| MQTL11.9 | Os11g0677400 | 11 | Pectinesterase inhibitor domain containing protein. (Os11t0677400-01) | PMEI43 |
| MQTL11.9 | Os11g0684100 | 11 | WRKY transcription factor 125, Response to *Xoo* infection (Os11t0684100-01);NB-ARC domain containing protein. (Os11t0684100-02) | WRKY125 |
| MQTL11.9 | Os11g0686250 | 11 | WRKY transcription factor 63, Negative regulation of chilling tolerance (Os11t0686250-01);WRKY transcription factor 63 (Os11t0686250-02) | WRKY63 |
| MQTL11.9 | Os11g0673600 | 11 | Similar to NB-ARC domain containing protein, expressed. (Os11t0673600-00) | YR2, YR6, YR9, YR10, YR21, YR23 |
| M-QTL12.5 | Os12g0526600 | 12 | Leucine-rich repeat, cysteine-containing subtype domain containing protein | OsFbox661 |
| M-QTL12.6 | Os12g0555500 | 12 | Pathogen resistance protein PBZ1, 17 kDa RNase, Disease resistance | **OsPR10a** |

**Table S3** Genes related to resistance (R) gene analogues (RGA) within the identified M-QTL

| **M-QTL** | **Gene stable ID** | **Chr** | **Gene description** |
| --- | --- | --- | --- |
| M-QTL1.3 | Os01g0335700 | 1 | NB-ARC domain containing protein |
| M-QTL1.3 | Os01g0351500 | 1 | Plant disease resistance response protein domain containing protein |
| M-QTL1.4 | Os01g0518651 | 1 | Leucine-rich repeat domain containing protein |
| M-QTL1.4 | Os01g0520600 | 1 | NB-ARC domain containing protein |
| M-QTL1.4 | Os01g0515300 | 1 | Similar to Leucine Rich Repeat family protein |
| M-QTL1.8 | Os01g0937300 | 1 | NB-ARC domain containing protein |
| M-QTL1.8 | Os01g0937400 | 1 | NB-ARC domain containing protein |
| M-QTL2.1 | Os02g0261800 | 2 | NB-ARC domain containing protein |
| M-QTL2.2 | Os02g0475500 | 2 | NB-ARC domain containing protein |
| M-QTL2.3 | Os02g0597300 | 2 | Leucine-rich repeat domain containing protein |
| M-QTL2.3 | Os02g0610000 | 2 | Leucine-rich repeat domain containing protein |
| M-QTL2.3 | Os02g0614966 | 2 | Leucine-rich repeat domain containing protein |
| M-QTL2.3 | Os02g0615400 | 2 | Leucine-rich repeat domain containing protein |
| M-QTL2.3 | Os02g0609900 | 2 | Leucine-rich repeat, N-terminal domain containing protein |
| M-QTL2.3 | Os02g0612200 | 2 | NB-ARC domain containing protein |
| M-QTL2.5 | Os02g0739400 | 2 | Similar to Avr9/Cf-9 rapidly elicited protein 231 precursor |
| M-QTL3.1 | Os03g0324600 | 3 | NB-ARC domain containing protein |
| M-QTL4.1 | Os04g0205200 | 4 | NB-ARC domain containing protein |
| M-QTL4.1 | Os04g0169500 | 4 | Similar to Avr9/Cf-9 rapidly elicited protein 137 |
| M-QTL4.1 | Os04g0202700 | 4 | Similar to Avr9/Cf-9 rapidly elicited protein 256 (Fragment) |
| M-QTL4.3 | Os04g0349700 | 4 | Leucine-rich repeat, typical subtype containing protein |
| M-QTL4.4 | Os04g0503500 | 4 | Leucine-rich repeat, cysteine-containing subtype containing protein |
| M-QTL4.4 | Os04g0512900 | 4 | NB-ARC domain containing protein |
| M-QTL4.4 | Os04g0514600 | 4 | Similar to NBS-LRR type disease resistance protein O2 (Fragment) |
| M-QTL4.6 | Os04g0672600 | 4 | Leucine-rich repeat, N-terminal domain containing protein |
| M-QTL5.1 | Os05g0170300 | 5 | Leucine-rich repeat domain containing protein |
| M-QTL5.1 | Os05g0170700 | 5 | Leucine-rich repeat domain containing protein |
| M-QTL5.1 | Os05g0143550 | 5 | Similar to Blast and wounding induced mitogen-activated protein kinase |
| M-QTL5.1 | Os05g0143600 | 5 | Similar to Jasmonate-induced protein |
| M-QTL5.2 | Os05g0257100 | 5 | Leucine-rich repeat, plant specific containing protein |
| M-QTL5.3 | Os05g0479700 | 5 | NB-ARC domain containing protein |
| M-QTL5.3 | Os05g0491400 | 5 | Similar to LRR protein |
| M-QTL5.4 | Os05g0524600 | 5 | Leucine-rich repeat domain containing protein |
| M-QTL5.4 | Os05g0522600 | 5 | Leucine-rich repeat, plant specific containing protein |
| M-QTL6.1 | Os06g0140300 | 6 | Leucine-rich repeat, N-terminal domain containing protein |
| M-QTL6.1 | Os06g0140200 | 6 | Leucine-rich repeat, plant specific containing protein |
| M-QTL6.1 | Os06g0142650 | 6 | Similar to Avr9/Cf-9 rapidly elicited protein 11 (Fragment) |
| M-QTL6.1 | Os06g0125000 | 6 | Similar to NBS-LRR disease resistance protein homologue |
| M-QTL6.2 | Os06g0186300 | 6 | Leucine-rich repeat domain containing protein |
| M-QTL6.2 | Os06g0179800 | 6 | Leucine-rich repeat, N-terminal domain containing protein |
| M-QTL6.2 | Os06g0167500 | 6 | Leucine-rich repeat, plant specific containing protein |
| M-QTL6.3 | Os06g0276300 | 6 | NB-ARC domain containing protein |
| M-QTL6.3 | Os06g0287000 | 6 | Similar to NBS-LRR type R protein, Nbs4-Pi |
| M-QTL6.4 | Os06g0478600 | 6 | Leucine-rich repeat, N-terminal domain containing protein |
| M-QTL6.5 | Os06g0557400 | 6 | Leucine-rich repeat domain containing protein |
| M-QTL6.6 | Os06g0627500 | 6 | Leucine-rich repeat, plant specific containing protein |
| M-QTL6.6 | Os06g0619000 | 6 | NB-ARC domain containing protein |
| M-QTL8.3 | Os08g0387700 | 8 | NB-ARC domain containing protein |
| M-QTL8.3 | Os08g0388300 | 8 | NB-ARC domain containing protein |
| M-QTL8.3 | Os08g0396700 | 8 | NB-ARC domain containing protein |
| M-QTL8.3 | Os08g0375400 | 8 | Plant disease resistance response protein family protein |
| M-QTL8.3 | Os08g0386700 | 8 | Similar to Avr9 elicitor response protein-like |
| M-QTL8.3 | Os08g0376300 | 8 | Similar to Leucine-rich receptor-like protein kinase |
| M-QTL10.1 | Os10g0161400 | 10 | NB-ARC domain containing protein |
| M-QTL10.1 | Os10g0163040 | 10 | Similar to Blast resistance protein |
| M-QTL10.1 | Os10g0162832 | 10 | Similar to LRR14 |
| M-QTL10.1 | Os10g0162846 | 10 | Similar to LRR14 |
| M-QTL10.2 | Os10g0336300 | 10 | Leucine-rich repeat domain containing protein |
| M-QTL10.2 | Os10g0370100 | 10 | NB-ARC domain containing protein |
| M-QTL10.2 | Os10g0333700 | 10 | Plant disease resistance response protein domain containing protein |
| MQTL11.1 | Os11g0208900 | 11 | Leucine rich repeat containing protein kinase. (Os11t0208900-01) |
| MQTL11.1 | Os11g0213700 | 11 | Leucine-rich repeat, typical subtype containing protein. (Os11t0213700-00) |
| MQTL11.1 | Os11g0207400 | 11 | NB-ARC domain containing protein. (Os11t0207400-01) |
| MQTL11.1 | Os11g0222900 | 11 | NB-ARC domain containing protein. (Os11t0222900-01);NB-ARC domain containing protein. (Os11t0222900-02) |
| MQTL11.1 | Os11g0212000 | 11 | Similar to Leucine Rich Repeat family protein, expressed. (Os11t0212000-00) |
| MQTL11.1 | Os11g0211600 | 11 | Similar to Leucine Rich Repeat family protein. (Os11t0211600-00) |
| MQTL11.1 | Os11g0211650 | 11 | Similar to Leucine Rich Repeat family protein. (Os11t0211650-00) |
| MQTL11.1 | Os11g0223201 | 11 | Similar to NB-ARC domain containing protein, expressed. (Os11t0223201-01) |
| MQTL11.1 | Os11g0211300 | 11 | Similar to NBS-LRR disease resistance protein homologue (Fragment). (Os11t0211300-01) |
| MQTL11.1 | Os11g0213800 | 11 | Similar to NBS-LRR disease resistance protein homologue (Fragment). (Os11t0213800-01) |
| M-QTL11.2 | Os11g0249000 | 11 | NB-ARC domain containing protein |
| MQTL11.3 | Os11g0265900 | 11 | Conserved hypothetical protein. (Os11t0265900-01);NB-ARC domain containing protein. (Os11t0265900-02) |
| MQTL11.3 | Os11g0270500 | 11 | NB-ARC domain containing protein. (Os11t0270500-01) |
| M-QTL11.3 | Os11g0263000 | 11 | Similar to Leucine Rich Repeat family protein, expressed |
| M-QTL11.5 | Os11g0480000 | 11 | NB-ARC domain containing protein |
| M-QTL11.5 | Os11g0481150 | 11 | NB-ARC domain containing protein |
| M-QTL11.5 | Os11g0482200 | 11 | Pathogenic type III effector avirulence factor Avr cleavage site domain containing protein |
| M-QTL11.5 | Os11g0479400 | 11 | Similar to Leucine Rich Repeat family protein |
| M-QTL11.5 | Os11g0493700 | 11 | Similar to NBS-LRR type resistance protein (Fragment) |
| M-QTL11.5 | Os11g0492900 | 11 | Similar to NBS-LRR type resistance protein (Fragment) |
| M-QTL11.5 | Os11g0494100 | 11 | Similar to NBS-LRR type resistance protein (Fragment) |
| MQTL11.7 | Os11g0583200 | 11 | Hypothetical conserved gene. (Os11t0583200-01) |
| MQTL11.7 | Os11g0598300 | 11 | NB-ARC domain containing protein. (Os11t0598300-00) |
| MQTL11.7 | Os11g0587100 | 11 | Pentatricopeptide repeat domain containing protein. (Os11t0587100-00) |
| MQTL11.7 | Os11g0597400 | 11 | Similar to Leucine Rich Repeat family protein, expressed. (Os11t0597400-00) |
| MQTL11.7 | Os11g0588400 | 11 | Similar to NB-ARC domain containing protein, expressed. (Os11t0588400-01); (Os11t0588400-02) |
| MQTL11.7 | Os11g0592500 | 11 | Similar to NB-ARC domain containing protein, expressed. (Os11t0592500-01); Conserved hypothetical protein. (Os11t0592500-02) |
| MQTL11.7 | Os11g0589800 | 11 | Similar to NB-ARC domain containing protein. (Os11t0589800-00) |
| M-QTL11.7 | Os11g0579100 | 11 | NB-ARC domain containing protein |
| M-QTL11.7 | Os11g0579200 | 11 | Similar to Leucine Rich Repeat family protein, expressed |
| M-QTL11.7 | Os11g0579400 | 11 | Similar to Leucine Rich Repeat family protein, expressed |
| MQTL11.8 | Os11g0629600 | 11 | NB-ARC domain containing protein. (Os11t0629600-01);Similar to Leucine Rich Repeat family protein, expressed. (Os11t0629600-02) |
| MQTL11.8 | Os11g0628000 | 11 | Protein kinase, catalytic domain domain containing protein. (Os11t0628000-00) |
| MQTL11.8 | Os11g0629550 | 11 | Similar to Disease resistance protein RPM1 homolog. (Os11t0629550-00) |
| MQTL11.8 | Os11g0620500 | 11 | Tyrosine protein kinase domain containing protein. (Os11t0620500-01) |
| MQTL11.9 | Os11g0686500 | 11 | NB-ARC domain containing protein. (Os11t0686500-01) |
| MQTL11.9 | Os11g0687800 | 11 | NB-ARC domain containing protein. (Os11t0687800-01);NB-ARC domain containing protein. (Os11t0687800-02) |
| MQTL11.9 | Os11g0673000 | 11 | Pentatricopeptide repeat domain containing protein. (Os11t0673000-00) |
| MQTL11.9 | Os11g0680200 | 11 | Pentatricopeptide repeat domain containing protein. (Os11t0680200-00) |
| MQTL11.9 | Os11g0674400 | 11 | Similar to NB-ARC domain containing protein, expressed. (Os11t0674400-00) |
| MQTL11.9 | Os11g0676100 | 11 | Similar to NB-ARC domain containing protein, expressed. (Os11t0676100-00) |
| MQTL11.9 | Os11g0677000 | 11 | Similar to NB-ARC domain containing protein, expressed. (Os11t0677000-00) |
| MQTL11.9 | Os11g0678400 | 11 | Similar to NB-ARC domain containing protein, expressed. (Os11t0678400-00) |
| MQTL11.9 | Os11g0684700 | 11 | Similar to NB-ARC domain containing protein, expressed. (Os11t0684700-01) |
| MQTL11.9 | Os11g0686900 | 11 | Similar to NB-ARC domain containing protein, expressed. (Os11t0686900-00) |
| MQTL11.9 | Os11g0676800 | 11 | Similar to NB-ARC domain containing protein. (Os11t0676800-00) |
| MQTL11.9 | Os11g0676200 | 11 | Similar to NBS-LRR-like protein NBA2 (Fragment). (Os11t0676200-00) |
| M-QTL12.1 | Os12g0174700 | 12 | Similar to disease resistance response protein 206 |
| M-QTL12.2 | Os12g0211500 | 12 | Leucine-rich repeat, N-terminal domain containing protein |
| M-QTL12.2 | Os12g0199100 | 12 | NB-ARC domain containing protein |
| M-QTL12.2 | Os12g0204600 | 12 | NB-ARC domain containing protein |
| M-QTL12.2 | Os12g0205500 | 12 | NB-ARC domain containing protein |
| M-QTL12.2 | Os12g0198700 | 12 | Similar to Jasmonate-induced protein |
| M-QTL12.2 | Os12g0199000 | 12 | Similar to Jasmonate-induced protein |
| M-QTL12.2 | Os12g0215950 | 12 | Similar to Leucine Rich Repeat family protein, expressed |
| M-QTL12.2 | Os12g0217400 | 12 | Similar to Leucine Rich Repeat family protein, expressed |
| M-QTL12.3 | Os12g0438300 | 12 | NB-ARC domain containing protein |
| M-QTL12.3 | Os12g0421000 | 12 | Similar to Barley stem rust resistance protein |
| M-QTL12.5 | Os12g0516300 | 12 | NB-ARC domain containing protein |
| M-QTL12.6 | Os12g0552900 | 12 | NB-ARC domain containing protein |
| M-QTL12.6 | Os12g0553200 | 12 | NB-ARC domain containing protein |
| M-QTL12.6 | Os12g0564800 | 12 | NB-ARC domain containing protein |
| M-QTL12.6 | Os12g0559300 | 12 | Similar to Leucine Rich Repeat family protein |
| M-QTL12.6 | Os12g0552700 | 12 | Similar to Leucine Rich Repeat family protein, expressed |
| M-QTL12.7 | Os12g0586000 | 12 | Similar to Disease resistance protein ADR1 (Activated disease resistance protein 1) |

**Table S4** M-QTL region encompassing genes associated with rice blast resistance in terms of trait ontology, gene description and known candidate genes

| **M-QTL** | **Gene name** | **Gene ID** | **Gene start (bp)** | **Gene end (bp)** | **Blast resistance basis** |
| --- | --- | --- | --- | --- | --- |
| M-QTL1.5 | AP2/EREBP#127 | Os01g0693400 | 28619585 | 28621117 | Characterised RGA |
| M-QTL1.5 | C10728, OsMPK20-4 | Os01g0665200 | 26885484 | 26886719 | Characterised RGA/ Trait ontology |
| M-QTL1.1 | CSB | Os01g0102800 | 152853 | 156449 | Trait ontology |
| M-QTL1.2 | DPF | Os01g0196300 | 5201862 | 5203996 | Trait ontology |
| M-QTL1.3 | IPI1 | Os01g0350900 | 14012515 | 14019465 | Trait ontology |
| M-QTL1.5 | LRR-RLK | Os01g0694100 | 28669652 | 28673566 | Characterised RGA |
| M-QTL1.4 | miR159a | Os01g0510100 | 17681439 | 17683119 | Trait ontology |
| M-QTL1.2 | NH1 | Os01g0194300 | 5060605 | 5065209 | Gene description |
| M-QTL1.3 | Os01g0326300 | Os01g0343200 | 12517993 | 12519238 | Trait ontology |
| M-QTL1.3 | Os01g0343200 | Os01g0326000 | 13560832 | 13566871 | Gene description |
| M-QTL1.7 | Os01g0845100 | Os01g0845100 | 36296586 | 36298621 | Trait ontology |
| M-QTL1.7 | OsBAG4 | Os01g0831200 | 35582280 | 35585575 | Trait ontology |
| M-QTL1.2 | OsERF#039 | Os01g0200600 | 5454772 | 5455667 | Characterised RGA |
| M-QTL1.3 | OsJRL | Os01g0348900 | 13903285 | 13904626 | Trait ontology |
| M-QTL1.5 | OsLSK1 | Os01g0669100 | 27417284 | 27420084 | Characterised RGA |
| M-QTL1.4 | OsMKK6 | Os01g0517500 | 17904834 | 17910037 | Trait ontology |
| M-QTL1.7 | OsMPG1 | Os01g0847200 | 36395486 | 36398031 | Trait ontology |
| M-QTL1.5 | OsMPK20-4 | Os01g0660200 | 27171290 | 27178237 | Gene description |
| M-QTL1.4 | OsMYBS1 | Os01g0524500 | 18755303 | 18756817 | Trait ontology |
| M-QTL1.7 | OsNPR4 | Os01g0837000 | 35885095 | 35888562 | Trait ontology |
| M-QTL1.4 | OsNRAMP6 | Os01g0503400 | 17454023 | 17464973 | Gene description |
| M-QTL1.4 | OsPGL3 | Os01g0507000 | 18352836 | 18354506 | Gene description |
| M-QTL1.5 | OsRFP | Os01g0667700 | 27303264 | 27312300 | Trait ontology |
| M-QTL1.8 | OsRLCK55 | Os01g0936100 | 41095911 | 41100367 | Trait ontology |
| M-QTL1.3 | prx12 | Os01g0326300 | 12499923 | 12504209 | Trait ontology |
| M-QTL1.5 | RACK1 | Os01g0686800 | 28330800 | 28333317 | Trait ontology |
| M-QTL1.7 | Rpp17 | Os01g0841700 | 36135911 | 36137281 | Characterised RGA |
| M-QTL1.5 | TPC1 | Os01g0678500 | 27906660 | 27920949 | Characterised RGA |
| M-QTL10.1 | Os10g0163040 | Os10g0163040 | 4262515 | 4266433 | Gene description |
| M-QTL10.2 | Os10g0351700 | Os10g0349900 | 10727319 | 10730929 | Gene description |
| M-QTL10.2 | Os10g0361000 | Os10g0361000 | 11110251 | 11110999 | Trait ontology |
| M-QTL10.2 | Os10g0370500 | Os10g0370500 | 11666794 | 11673481 | Trait ontology |
| M-QTL10.3 | Os10g0537800 | Os10g0537800 | 20956884 | 20958287 | Trait ontology |
| M-QTL10.3 | OsBSK1-2 | Os10g0542800 | 21199763 | 21204782 | Trait ontology |
| M-QTL10.3 | OsBSR-K1 | Os10g0548200 | 21445880 | 21453580 | Trait ontology |
| M-QTL10.2 | OsHyPRP12 | Os10g0351700 | 10587298 | 10587959 | Trait ontology |
| M-QTL10.3 | OsHyPRP18 | Os10g0552600 | 21690332 | 21691088 | Trait ontology |
| M-QTL10.3 | OsHyPRP19 | Os10g0552700 | 21693876 | 21694502 | Trait ontology |
| M-QTL10.3 | OsMLO4 | Os10g0541000 | 21109457 | 21114148 | Trait ontology |
| M-QTL10.3 | OsPUB53 | Os10g0548300 | 21454034 | 21459741 | Trait ontology |
| M-QTL10.3 | OsPUB67 | Os10g0552400 | 21679094 | 21681430 | Trait ontology |
| M-QTL10.1 | OsVAMP714 | Os10g0154000 | 3358062 | 3360552 | Characterised RGA |
| M-QTL10.3 | prx128 | Os10g0536700 | 20900132 | 20902329 | Trait ontology |
| M-QTL10.3 | RLCK306 | Os10g0548700 | 21467611 | 21472768 | Characterised RGA/ Trait ontology |
| M-QTL11.9 | BGLU36 | Os11g0683500 | 27571044 | 27574081 | Trait ontology |
| M-QTL11.9 | DRP-RP13 | Os11g0673600 | 27223472 | 27224956 | Characterised RGA |
| M-QTL11.3 | MPK15 | Os11g0271100 | 9471589 | 9479354 | Trait ontology |
| M-QTL11.1 | Os11gRGA3 | Os11g0213500 | 5895364 | 5897167 | Characterised RGA |
| M-QTL11.1 | Os11gRGA5, RGA5, Pia | Os11g0225300 | 5223773 | 5225109 | Characterised blast resistance genes/RGA |
| M-QTL11.3 | OsFbox598 | Os11g0264200 | 8965406 | 8970029 | Characterised RGA |
| M-QTL11.1 | OsRLCK315 | Os11g0194900 | 4734980 | 4738431 | Trait ontology |
| M-QTL11.7 | Pb1, Xa39 | Os11g0588600 | 22328967 | 22333982 | Characterised RGA |
| M-QTL11.7 | Pb1, Xa39 | Os11g0598500 | 22862447 | 22866319 | Characterised blast resistance genes/RGA |
| M-QTL11.9 | Pb2 | Os11g0682600 | 27605099 | 27609143 | Characterised blast resistance genes |
| M-QTL11.8 | Pi54, Pi-kh, RPP8 | Os11g0639100 | 25263336 | 25264276 | Characterised blast resistance genes/RGA |
| M-QTL11.8 | PIKH | Os11g0633500 | 24911501 | 24917044 | Characterised RGA |
| M-QTL11.9 | PIKM, PIK | Os11g0673900 | 27234759 | 27237260 | Characterised RGA |
| M-QTL11.9 | PIKM, PIK | Os11g0677400 | 27366801 | 27368303 | Characterised RGA |
| M-QTL11.9 | Pikp-1, Pikm5-NP, Pik5-NP, xa47 | Os11g0688832 | 27984697 | 27989128 | Characterised blast resistance genes/RGA |
| M-QTL11.9 | Pikp-2, Pikm6-NP, Pik6-NP | Os11g0689100 | 27978368 | 27983597 | Characterised blast resistance genes/RGA |
| M-QTL11.9 | PME33 | Os11g0683700 | 27656251 | 27658409 | Characterised RGA |
| M-QTL11.9 | PME34 | Os11g0683800 | 27660470 | 27662654 | Characterised RGA |
| M-QTL11.9 | PMEI43 | Os11g0684100 | 27683640 | 27695070 | Characterised RGA |
| M-QTL11.1 | PXLG2 | Os11g0206700 | 5378426 | 5383879 | Trait ontology |
| M-QTL11.1 | RGA4 | Os11g0224900 | 6532813 | 6535926 | Characterised RGA |
| M-QTL11.1 | RGA5, Pia | Os11g0225100 | 6541924 | 6546026 | Characterised blast resistance genes/RGA |
| M-QTL11.1 | RLCK319 | Os11g0225000 | 6540629 | 6541618 | Trait ontology |
| M-QTL11.9 | RLCK352 | Os11g0681400 | 27648703 | 27655229 | Trait ontology |
| M-QTL11.7 | RPP13-like protein 3 | Os11g0590700 | 22427774 | 22435407 | Characterised RGA |
| M-QTL11.1 | SODX,SMK1 | Os11g0203300 | 6554522 | 6561687 | Characterised RGA/ Gene description |
| M-QTL11.8 | SPL-D,OsGRDP1 | Os11g0621300 | 24218780 | 24224440 | Trait ontology |
| M-QTL11.9 | WRKY125 | Os11g0686250 | 27784700 | 27785295 | Characterised RGA |
| M-QTL11.9 | WRKY63, OsvWA37 | Os11g0688832 | 27978368 | 27983597 | Characterised RGA |
| M-QTL11.9 | YR2, YR6, YR9, YR10, YR21, YR23, OsvWA36 | Os11g0689100 | 27984697 | 27989128 | Characterised RGA |
| M-QTL12.7 | Os12g0593000 | Os12g0593000 | 24872484 | 24877348 | Trait ontology |
| M-QTL12.1 | OsCBSCBSPB4 | Os12g0169400 | 3531395 | 3536426 | Trait ontology |
| M-QTL12.5 | OsFbox661 | Os12g0526600 | 20749132 | 20752135 | Characterised RGA |
| M-QTL12.5 | OsPAL03 | Os12g0520200 | 20292893 | 20295400 | Trait ontology |
| M-QTL12.6 | OsPR10a | Os12g0555500 | 22600049 | 22601182 | Characterised RGA/ Trait ontology |
| M-QTL12.1 | OsPUB37 | Os12g0161100 | 3068752 | 3070072 | Trait ontology |
| M-QTL12.6 | RPR10b | Os12g0555200 | 22591681 | 22593505 | Trait ontology |
| M-QTL12.6 | RSOsPR10 | Os12g0555000 | 22580880 | 22581936 | Trait ontology |
| M-QTL2.1 | Docs1 | Os02g0236100 | 7711097 | 7715739 | Characterised RGA |
| M-QTL2.5 | Os_F0767 | Os02g0759700 | 31986128 | 31989420 | Characterised RGA |
| M-QTL2.3 | Os02g0584700 | Os02g0584700 | 22550855 | 22551584 | Trait ontology |
| M-QTL2.3 | Os02g0584800 | Os02g0584800 | 22553380 | 22554242 | Trait ontology |
| M-QTL2.3 | Os02g0621700 | Os02g0621700 | 24741811 | 24747949 | Trait ontology |
| M-QTL2.5 | Os02g0743100 | Os02g0745600 | 31141034 | 31142384 | Trait ontology |
| M-QTL2.5 | Os02g0745600 | Os02g0759400 | 31290670 | 31299077 | Trait ontology |
| M-QTL2.5 | Os02g0764500 | Os02g0743100 | 32199542 | 32201608 | Gene description |
| M-QTL2.3 | OsCPS2 | Os02g0571100 | 21841092 | 21847545 | Trait ontology |
| M-QTL2.3 | Oscyp71Z6 | Os02g0570500 | 21788170 | 21791222 | Trait ontology |
| M-QTL2.2 | OsGLP2-1 | Os02g0491600 | 17165188 | 17165997 | Trait ontology |
| M-QTL2.4 | OsGpx3 | Os02g0664000 | 26929770 | 26932403 | Trait ontology |
| M-QTL2.3 | OsIRL2 | Os02g0593600 | 22978830 | 22981452 | Characterised RGA |
| M-QTL2.3 | OsKS7 | Os02g0570400 | 21765011 | 21773418 | Trait ontology |
| M-QTL2.3 | OsMYB30 | Os02g0624300 | 24878777 | 24879932 | Trait ontology |
| M-QTL2.4 | OsPUB60 | Os02g0665400 | 27023005 | 27028418 | Trait ontology |
| M-QTL2.2 | OsPUB8 | Os02g0488600 | 16981502 | 16985338 | Trait ontology |
| M-QTL2.5 | OsRING-1 | Os02g0764500 | 31967805 | 31969537 | Trait ontology |
| M-QTL2.2 | OsSNAP32 | Os02g0437200 | 13949431 | 13951620 | Gene description |
| M-QTL2.1 | OsStr12 | Os02g0257300 | 8873101 | 8875996 | Trait ontology |
| M-QTL2.1 | prx27 | Os02g0236600 | 7751287 | 7753674 | Trait ontology |
| M-QTL2.1 | rNBS41 | Os02g0262800 | 9257998 | 9262614 | Characterised RGA |
| M-QTL2.1 | UBC5B | Os02g0261100 | 9116165 | 9119456 | Characterised RGA |
| M-QTL3.1 | Os03g0328000 | Os03g0328000 | 12006546 | 12007780 | Trait ontology |
| M-QTL3.2 | OsBBS1 | Os03g0364400 | 14206981 | 14209160 | Trait ontology |
| M-QTL3.4 | OsBIHD1 | Os03g0680800 | 27057033 | 27063684 | Trait ontology |
| M-QTL3.5 | OsGDH1 | Os03g0794500 | 33038059 | 33041471 | Trait ontology |
| M-QTL3.4 | OsGRF9 | Os03g0674700 | 26679255 | 26682044 | Trait ontology |
| M-QTL4.5 | ACS2 | Os04g0578000 | 29131883 | 29134682 | Trait ontology |
| M-QTL4.6 | ARF8 | Os04g0671900 | 34278477 | 34285845 | Trait ontology |
| M-QTL4.4 | DCL4 | Os04g0509300 | 25474092 | 25489203 | Trait ontology |
| M-QTL4.1 | Os04g0169500 | Os04g0169500 | 4769960 | 4770679 | Trait ontology |
| M-QTL4.1 | Os04g0177300 | Os04g0177300 | 5254429 | 5260631 | Trait ontology |
| M-QTL4.1 | Os04g0206700 | Os04g0206700 | 7163748 | 7166358 | Trait ontology |
| M-QTL4.3 | Os04g0352066 | Os04g0352066 | 16801977 | 16802287 | Gene description |
| M-QTL4.6 | OsCIN5 | Os04g0664900 | 33943991 | 33946513 | Trait ontology |
| M-QTL4.1 | OsCPS4 | Os04g0178300 | 5318060 | 5326425 | Trait ontology |
| M-QTL4.4 | OsERF#045 | Os04g0529100 | 26438670 | 26440540 | Characterised RGA |
| M-QTL4.4 | OsFbox227 | Os04g0505700 | 25244494 | 25245978 | Characterised RGA |
| M-QTL4.1 | OsKS4 | Os04g0179700 | 5428347 | 5434023 | Trait ontology |
| M-QTL4.1 | OsMAS | Os04g0179200 | 5385722 | 5387215 | Trait ontology |
| M-QTL4.4 | OsMyb4 | Os04g0517100 | 25843038 | 25844912 | Trait ontology |
| M-QTL4.4 | OsPAL02 | Os04g0518100 | 25906878 | 25910059 | Trait ontology |
| M-QTL4.4 | OsPAL07 | Os04g0518400 | 25927077 | 25929716 | Trait ontology |
| M-QTL4.4 | OsRLCK153 | Os04g0503600 | 25140210 | 25145151 | Trait ontology |
| M-QTL4.4 | OsS40-13 | Os04g0520700 | 26074703 | 26075861 | Trait ontology |
| M-QTL4.5 | SOD | Os04g0573200 | 28874420 | 28882313 | Gene description |
| M-QTL5.1 | APIP6 | Os05g0160300 | 3189795 | 3195554 | Trait ontology |
| M-QTL5.3 | ARF | Os05g0489600 | 24057380 | 24060607 | Trait ontology |
| M-QTL5.1 | Os05g0143550 | Os05g0135800 | 2516123 | 2520599 | Gene description |
| M-QTL5.1 | Os05g0150300 | Os05g0154600 | 2874325 | 2884462 | Trait ontology |
| M-QTL5.1 | Os05g0164900 | Os05g0164900 | 3813306 | 3819768 | Trait ontology |
| M-QTL5.3 | Os05g0498700 | Os05g0498700 | 24552461 | 24555115 | Trait ontology |
| M-QTL5.4 | OsHXK5 | Os05g0522500 | 26017332 | 26022265 | Trait ontology |
| M-QTL5.1 | OsLTPd8 | Os05g0143550 | 3539620 | 3540966 | Gene description |
| M-QTL5.3 | OsMC4 | Os05g0496400 | 24383180 | 24384097 | Trait ontology |
| M-QTL5.3 | OsMC5 | Os05g0496500 | 24384902 | 24387337 | Trait ontology |
| M-QTL5.3 | OsRPK1 | Os05g0486100 | 23899480 | 23905448 | Characterised RGA |
| M-QTL5.1 | RLCK178 | Os05g0150300 | 2096145 | 2100423 | Trait ontology |
| M-QTL5.3 | YR1 | Os05g0492200 | 24184504 | 24189203 | Characterised RGA |
| M-QTL5.3 | YR48 | Os05g0492600 | 24197060 | 24200694 | Characterised RGA |
| M-QTL6.6 | BZIP50 | Os06g0622700 | 25041945 | 25043935 | Trait ontology |
| M-QTL6.2 | CHR702 | Os06g0193400 | 4167296 | 4174105 | Trait ontology |
| M-QTL6.5 | KO4 | Os06g0569500 | 22020872 | 22028146 | Trait ontology |
| M-QTL6.1 | LMR | Os06g0130000 | 1588473 | 1602310 | Trait ontology |
| M-QTL6.3 | Nbs1-Pi9 | Os06g0286500 | 10375846 | 10380216 | Characterised blast resistance genes/RGA |
| M-QTL6.1 | Os06g0111400 | Os06g0111400 | 634262 | 640450 | Trait ontology |
| M-QTL6.1 | Os06g0112400 | Os06g0112400 | 699114 | 702164 | Trait ontology |
| M-QTL6.1 | Os06g0128800 | Os06g0128800 | 1526538 | 1527491 | Trait ontology |
| M-QTL6.1 | Os06g0129100 | Os06g0129100 | 1539031 | 1542210 | Trait ontology |
| M-QTL6.2 | Os06g0172800 | Os06g0183800 | 3675795 | 3679085 | Trait ontology |
| M-QTL6.1 | OsACBP2 | Os06g0115300 | 860908 | 862566 | Trait ontology |
| M-QTL6.1 | OsDjA9 | Os06g0116800 | 927339 | 932583 | Trait ontology |
| M-QTL6.6 | OsFbox316 | Os06g0605900 | 24033805 | 24036789 | Characterised RGA |
| M-QTL6.6 | OsGAMYBL1 | Os06g0605600 | 24005166 | 24012308 | Trait ontology |
| M-QTL6.1 | OsGLIP1 | Os06g0129600 | 1565577 | 1573819 | Trait ontology |
| M-QTL6.2 | OsGRF2 | Os06g0204800 | 5300136 | 5301473 | Trait ontology |
| M-QTL6.1 | OsGRF5 | Os06g0116200 | 893174 | 896356 | Trait ontology |
| M-QTL6.6 | Osi-SAP8 | Os06g0612800 | 24491993 | 24493907 | Trait ontology |
| M-QTL6.2 | OsPTF1 | Os06g0163000 | 4709744 | 4715552 | Gene description |
| M-QTL6.1 | OsPUB51 | Os06g0140800 | 2140055 | 2146616 | Trait ontology |
| M-QTL6.2 | OsPUB70 | Os06g0166900 | 3175806 | 3181638 | Trait ontology |
| M-QTL6.5 | OsRab11 | Os06g0551400 | 20896135 | 20901653 | Characterised RGA |
| M-QTL6.5 | OsRab11 | Os06g0554100 | 21060826 | 21061895 | Trait ontology |
| M-QTL6.2 | OsRLCK199 | Os06g0168800 | 3360524 | 3363523 | Trait ontology |
| M-QTL6.2 | OsRLCK200 | Os06g0172800 | 3460493 | 3465373 | Trait ontology |
| M-QTL6.5 | OsRLCK206 | Os06g0541600 | 20330315 | 20338404 | Trait ontology |
| M-QTL6.2 | OsWR4 | Os06g0181700 | 4040784 | 4041763 | Characterised RGA |
| M-QTL6.3 | Piz | Os06g0286700 | 10387793 | 10390465 | Characterised blast resistance genes |
| M-QTL6.5 | prx86 | Os06g0547400 | 20705378 | 20706934 | Trait ontology |
| M-QTL7.6 | CHR705 | Os07g0668900 | 27825945 | 27831979 | Trait ontology |
| M-QTL7.6 | Cu/Zn-SOD | Os07g0660200 | 28075600 | 28077583 | Gene description |
| M-QTL7.4 | Os07g0481400 | Os07g0481400 | 17529809 | 17534987 | Trait ontology |
| M-QTL7.6 | Os07g0663900 | Os07g0658300 | 28013811 | 28014713 | Gene description |
| M-QTL7.6 | Os07g0680500 | Os07g0680500 | 28832616 | 28834725 | Trait ontology |
| M-QTL7.1 | OsALDH6B2 | Os07g0188800 | 4734626 | 4741454 | Trait ontology |
| M-QTL7.2 | OsERF#057 | Os07g0227600 | 7139927 | 7140775 | Characterised RGA |
| M-QTL7.4 | OsGRF11 | Os07g0467500 | 16622696 | 16625277 | Trait ontology |
| M-QTL7.6 | OsRLCK240 | Os07g0677200 | 28250047 | 28253655 | Trait ontology |
| M-QTL7.6 | POX22.3 | Os07g0663900 | 28673754 | 28675351 | Gene description |
| M-QTL7.6 | Spin6.2 | Os07g0665200 | 27713321 | 27722659 | Trait ontology |
| M-QTL8.4 | 4CL5 | Os08g0448000 | 21873076 | 21875497 | Trait ontology |
| M-QTL8.1 | GLP8-12 | Os08g0231400 | 7994404 | 7995717 | Trait ontology |
| M-QTL8.5 | Lox2:Os:1 | Os08g0511700 | 25216460 | 25224078 | Trait ontology |
| M-QTL8.5 | OsLOX8 | Os08g0509600 | 25241037 | 25250038 | Trait ontology |
| M-QTL8.1 | OsOSC12 | Os08g0224000 | 7545583 | 7551989 | Characterised RGA |
| M-QTL8.3 | OsWRKY69 | Os08g0386200 | 18220044 | 18221513 | Trait ontology |
| M-QTL8.5 | pi55-1 | Os08g0511700 | 25377754 | 25380592 | Characterised blast resistance genes |
| M-QTL8.5 | pi55-2 | Os08g0512200 | 25399831 | 25400422 | Characterised blast resistance genes |
| M-QTL8.5 | WFP | Os08g0512200 | 25274541 | 25278696 | Trait ontology |
| M-QTL9.1 | CabE | Os09g0296800 | 7185218 | 7188139 | Gene description |
| M-QTL9.2 | Os09g0327575 | Os09g0327800 | 9667216 | 9670256 | Gene description |
| M-QTL9.4 | OsRLCK278 | Os09g0533600 | 20962267 | 20967452 | Characterised RGA/Trait ontology |
| M-QTL9.3 | OsVIT2 | Os09g0396900 | 13831868 | 13833654 | Trait ontology |
| M-QTL9.2 | Pi3 | Os09g0327600 | 9672226 | 9674673 | Characterised blast resistance genes |
| M-QTL9.2 | Pi3 | Os09g0327800 | 9681913 | 9684808 | Characterised blast resistance genes |
| M-QTL9.1 | Sub1C | Os09g0286600 | 6387891 | 6389789 | Characterised RGA |

**Table S5** Pathways associated with rice blast resistance genes

| **Enrichment FDR** | **nGenes*** | **Total Genes**† | **Fold Enrichment** | **Functional annotation (domain/motif/pathway)** | **Genes*** |
| --- | --- | --- | --- | --- | --- |
| 0.0003 | 3 | 8 | 69.511 | Adenylate cyclase-modulating G protein-coupled receptor signaling pathway |  |
| 0.0007 | 3 | 11 | 50.553 | Cinnamic acid biosynthetic process | OsPAL02 OsPAL07 OsPAL03 |
| 0.0007 | 3 | 11 | 50.553 | Cinnamic acid metabolic process | OsPAL02 OsPAL07 OsPAL03 |
| 0.0002 | 5 | 41 | 22.605 | Diterpenoid biosynthetic process | OsKS7 OsCPS2 OsCPS4 OsKS4 KO4 |
| 0.0003 | 5 | 51 | 18.173 | Diterpenoid metabolic process | OsKS7 OsCPS2 OsCPS4 OsKS4 KO4 |
| 0.0002 | 8 | 170 | 8.723 | Reactive oxygen species metabolic process | prx12 prx27 SOD prx86 Cu/Zn-SOD POX22.3 prx128 |
| 0.0000 | 28 | 688 | 7.544 | Defense response | NH1 OsMKK6 TPC1 RACK1 OsKS7 OsCPS2 OsCPS4 OsKS4 DCL4 OsLTPd8 YR1 Nbs1-Pi9 OsOSC12 Pi3 OsMLO4 OsBSK1-2 Os11gRGA3 Os11gRGA4 Os11gRGA5 Xa39 OsWRKY125 Pik RPR10b OsPR10a |
| 0.0005 | 8 | 203 | 7.305 | Cellular oxidant detoxification | prx12 prx27 OsGpx3 SOD prx86 POX22.3 prx128 |
| 0.0007 | 8 | 215 | 6.897 | Cellular response to toxic substance | prx12 prx27 OsGpx3 SOD prx86 POX22.3 prx128 |
| 0.0007 | 8 | 215 | 6.897 | Cellular detoxification | prx12 prx27 OsGpx3 SOD prx86 POX22.3 prx128 |
| 0.0007 | 8 | 219 | 6.771 | Detoxification | prx12 prx27 OsGpx3 SOD prx86 POX22.3 prx128 |
| 0.0002 | 10 | 281 | 6.597 | Response to oxidative stress | prx12 prx27 OsGpx3 SOD prx86 OsALDH6B2 POX22.3 prx128 |
| 0.0000 | 18 | 637 | 5.238 | Protein ubiquitination | IPI1 OsRFP UBC5B OsPUB8 Os_F0767 OsFbox227 APIP6 OsPUB51 OsPUB70 OsFbox316 OsPUB53 RLCK306 OsPUB67 OsFbox598 OsPUB37 OsFbox661 |
| 0.0000 | 18 | 664 | 5.025 | Protein modification by small protein conjugation | IPI1 OsRFP UBC5B OsPUB8 Os_F0767 OsFbox227 APIP6 OsPUB51 OsPUB70 OsFbox316 OsPUB53 RLCK306 OsPUB67 OsFbox598 OsPUB37 OsFbox661 |
| 0.0000 | 45 | 1796 | 4.644 | Response to stress | NH1 prx12 OsMKK6 TPC1 RACK1 OsBAG4 prx27 OsKS7 OsCPS2 OsGpx3 OsGDH1 OsCPS4 OsKS4 DCL4 OsMyb4 SOD OsLTPd8 YR1 OsDjA9 Nbs1-Pi9 prx86 BZIP50 OsALDH6B2 POX22.3 OsOSC12 Pi3 OsVAMP714 prx128 OsMLO4 OsBSK1-2 Os11gRGA3 Os11gRGA4 Os11gRGA5 Xa39 OsWRKY125 Pik RPR10b OsPR10a |
| 0.0000 | 18 | 774 | 4.311 | Protein modification by small protein conjugation or removal | IPI1 OsRFP UBC5B OsPUB8 Os_F0767 OsFbox227 APIP6 OsPUB51 OsPUB70 OsFbox316 OsPUB53 RLCK306 OsPUB67 OsFbox598 OsPUB37 OsFbox661 |
| 0.0007 | 15 | 770 | 3.611 | Cellular response to chemical stimulus | NH1 prx12 prx27 OsGpx3 SOD ARF8 prx86 BZIP50 POX22.3 prx128 OsBSK1-2 RSOsPR10 RPR10b OsPR10a |
| 0.0000 | 60 | 3145 | 3.536 | Response to stimulus | NH1 prx12 OsMKK6 OsMYBS1 OsMPK20-4 TPC1 RACK1 OsBAG4 prx27 OsKS7 OsCPS2 OsGpx3 OsGDH1 OsCPS4 OsKS4 DCL4 OsMyb4 SOD ARF8 OsLTPd8 YR1 OsHXK5 OsDjA9 Nbs1-Pi9 OsRLCK206 prx86 OsRab11 BZIP50 OsALDH6B2 Spin6.2 POX22.3 OsOSC12 CabE Pi3 OsVAMP714 prx128 OsMLO4 OsBSK1-2 Os11gRGA3 Os11gRGA4 Os11gRGA5 OsMPK16 Xa39 OsWRKY125 Pik RSOsPR10 RPR10b OsPR10a |
| 0.0004 | 19 | 1100 | 3.202 | Signal transduction | NH1 OsMKK6 OsMPK20-4 TPC1 RACK1 ARF8 OsRLCK206 OsRab11 BZIP50 Spin6.2 OsBSK1-2 OsMPK16 RSOsPR10 RPR10b OsPR10a |
| 0.0004 | 19 | 1111 | 3.170 | Signaling | NH1 OsMKK6 OsMPK20-4 TPC1 RACK1 ARF8 OsRLCK206 OsRab11 BZIP50 Spin6.2 OsBSK1-2 OsMPK16 RSOsPR10 RPR10b OsPR10a |
| 0.0003 | 21 | 1271 | 3.063 | Cell communication | NH1 OsMKK6 OsMPK20-4 OsLSK1 TPC1 RACK1 OsGDH1 ARF8 OsRLCK206 OsRab11 BZIP50 Spin6.2 OsBSK1-2 OsMPK16 RSOsPR10 RPR10b OsPR10a |
| 0.0003 | 23 | 1473 | 2.894 | Protein phosphorylation | OsMKK6 OsMPK20-4 OsLSK1 RACK1 OsRLCK55 Docs1 OsBBS1 OsRLCK153 RLCK178 OsRPK1 OsPUB51 OsPUB70 OsRLCK199 OsRLCK200 OsRLCK240 OsRLCK278 OsBSK1-2 OsPUB53 RLCK306 OsRLCK315 OsRLCK319 OsMPK16 OsRLCK352 |
| 0.0001 | 28 | 1898 | 2.735 | Cellular response to stimulus | NH1 prx12 OsMKK6 OsMPK20-4 TPC1 RACK1 prx27 OsGpx3 OsGDH1 SOD ARF8 OsRLCK206 prx86 OsRab11 BZIP50 Spin6.2 POX22.3 prx128 OsBSK1-2 OsMPK16 RSOsPR10 RPR10b OsPR10a |
| 0.0000 | 43 | 3064 | 2.601 | Cellular protein modification process | IPI1 OsMKK6 OsMPK20-4 OsRFP OsLSK1 RACK1 OsRLCK55 Docs1 UBC5B OsPUB8 Os_F0767 OsBBS1 OsRLCK153 OsFbox227 RLCK178 APIP6 OsRPK1 ARF OsPUB51 OsPUB70 OsRLCK199 OsRLCK200 OsFbox316 OsRLCK240 CabE OsRLCK278 OsBSK1-2 OsPUB53 RLCK306 OsPUB67 OsRLCK315 OsRLCK319 OsFbox598 OsMPK16 OsRLCK352 OsPUB37 OsFbox661 RSOsPR10 RPR10b OsPR10a |
| 0.0000 | 43 | 3064 | 2.601 | Protein modification process | IPI1 OsMKK6 OsMPK20-4 OsRFP OsLSK1 RACK1 OsRLCK55 Docs1 UBC5B OsPUB8 Os_F0767 OsBBS1 OsRLCK153 OsFbox227 RLCK178 APIP6 OsRPK1 ARF OsPUB51 OsPUB70 OsRLCK199 OsRLCK200 OsFbox316 OsRLCK240 CabE OsRLCK278 OsBSK1-2 OsPUB53 RLCK306 OsPUB67 OsRLCK315 OsRLCK319 OsFbox598 OsMPK16 OsRLCK352 OsPUB37 OsFbox661 RSOsPR10 RPR10b OsPR10a |
| 0.0007 | 24 | 1754 | 2.536 | Phosphorylation | OsMKK6 OsMPK20-4 OsLSK1 RACK1 OsRLCK55 Docs1 OsBBS1 OsRLCK153 RLCK178 OsRPK1 OsHXK5 OsPUB51 OsPUB70 OsRLCK199 OsRLCK200 OsRLCK240 OsRLCK278 OsBSK1-2 OsPUB53 RLCK306 OsRLCK315 OsRLCK319 OsMPK16 OsRLCK352 |
| 0.0000 | 44 | 3484 | 2.341 | Macromolecule modification | IPI1 OsMKK6 OsMPK20-4 OsRFP OsLSK1 RACK1 OsRLCK55 Docs1 UBC5B OsPUB8 Os_F0767 OsBBS1 OsRLCK153 OsFbox227 RLCK178 APIP6 OsRPK1 ARF OsPUB51 OsPUB70 OsRLCK199 OsRLCK200 OsFbox316 OsRLCK240 CabE OsRLCK278 OsBSK1-2 OsPUB53 RLCK306 OsPUB67 OsRLCK315 SMK1 OsRLCK319 OsFbox598 OsMPK16 OsRLCK352 OsPUB37 OsFbox661 RSOsPR10 RPR10b OsPR10a |
| 0.0004 | 43 | 4043 | 1.971 | Regulation of biological process | NH1 DPF OsERF#039 OsMKK6 OsMYBS1 OsMPK20-4 TPC1 RACK1 AP2/EREBP#127 OsBAG4 Docs1 OsGpx3 OsGRF9 OsBIHD1 DCL4 OsMyb4 OsERF#045 ARF8 OsGRF5 OsWR4 OsPTF1 OsGRF2 OsRLCK206 OsRab11 BZIP50 OsERF#057 OsGRF11 Spin6.2 OsWRKY69 Sub1C OsBSK1-2 OsBSR-K1 OsMPK16 OsWRKY63 RSOsPR10 RPR10b OsPR10a |
| 0.0002 | 48 | 4575 | 1.945 | Biological regulation | NH1 DPF OsERF#039 OsNRAMP6 OsMKK6 OsMYBS1 OsMPK20-4 TPC1 RACK1 AP2/EREBP#127 OsBAG4 Docs1 OsGpx3 OsGRF9 OsBIHD1 DCL4 OsMyb4 OsERF#045 SOD ARF8 OsHXK5 OsGRF5 OsWR4 OsPTF1 OsGRF2 OsRLCK206 OsRab11 BZIP50 OsERF#057 OsGRF11 Spin6.2 OsWRKY69 Sub1C OsVIT2 OsBSK1-2 OsBSR-K1 OsMPK16 OsWRKY63 RSOsPR10 RPR10b OsPR10a |
| 0.0007 | 43 | 4170 | 1.911 | Cellular protein metabolic process | IPI1 OsMKK6 OsMPK20-4 OsRFP OsLSK1 RACK1 OsRLCK55 Docs1 UBC5B OsPUB8 Os_F0767 OsBBS1 OsRLCK153 OsFbox227 RLCK178 APIP6 OsRPK1 ARF OsPUB51 OsPUB70 OsRLCK199 OsRLCK200 OsFbox316 OsRLCK240 CabE OsRLCK278 OsBSK1-2 OsPUB53 RLCK306 OsPUB67 OsRLCK315 OsRLCK319 OsFbox598 OsMPK16 OsRLCK352 OsPUB37 OsFbox661 RSOsPR10 RPR10b OsPR10a |

**Table S6** Information of ten rice genotypes used for blast disease resistance response

| **Genotypes** | **Species** | **Subspecies** | **Genetic background** | **Phenotype** | **Parentage** |
| --- | --- | --- | --- | --- | --- |
| NR-2 | *Oryza sativa* | Indica | AA | R | Sneha/RR 149-1129 |
| NR-3 | *Oryza sativa* | Indica | AA | R | MTU 15/ Waikyaku |
| NR-7 | *Oryza sativa* | Indica | AA | R | IR 78875/IR 78877 |
| NR-9 | *Oryza sativa* | Indica | AA | R | IR 72022-46-2-3-2/IRRI 105 |
| NR-12 | *Oryza sativa* | Indica | AA | R | IRRI 76569-259-1-2-1/CT 6510-24-1-2. |
| NR-4 | *Oryza sativa* | Indica | AA | S | Gamma ray mutant from China 45 |
| NR-5 | *Oryza sativa* | Indica | AA | S | ARC 6650/CR 94-721 |
| NR-18 | *Oryza sativa* | Indica | AA | S | NDR 9370018/KDML 105/PSBRC 60 |
| NR-19 | *Oryza sativa* | Indica | AA | S | Udaya/IET 16611 |
| NR-46 | *Oryza sativa* | Indica | AA | S | Pankaj/Podumoni |


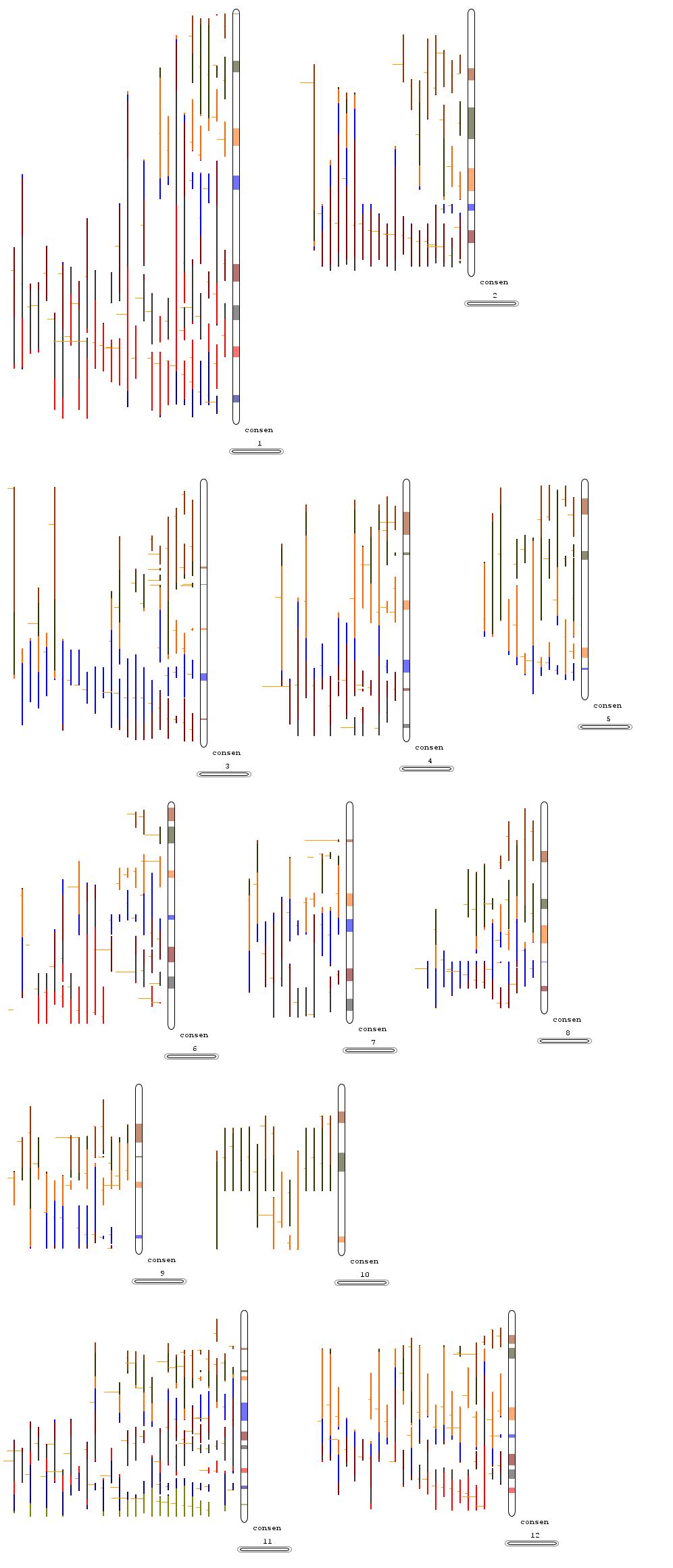


**Fig. S1** Distribution of M-QTL identified for rice blast resistance across the chromosomes


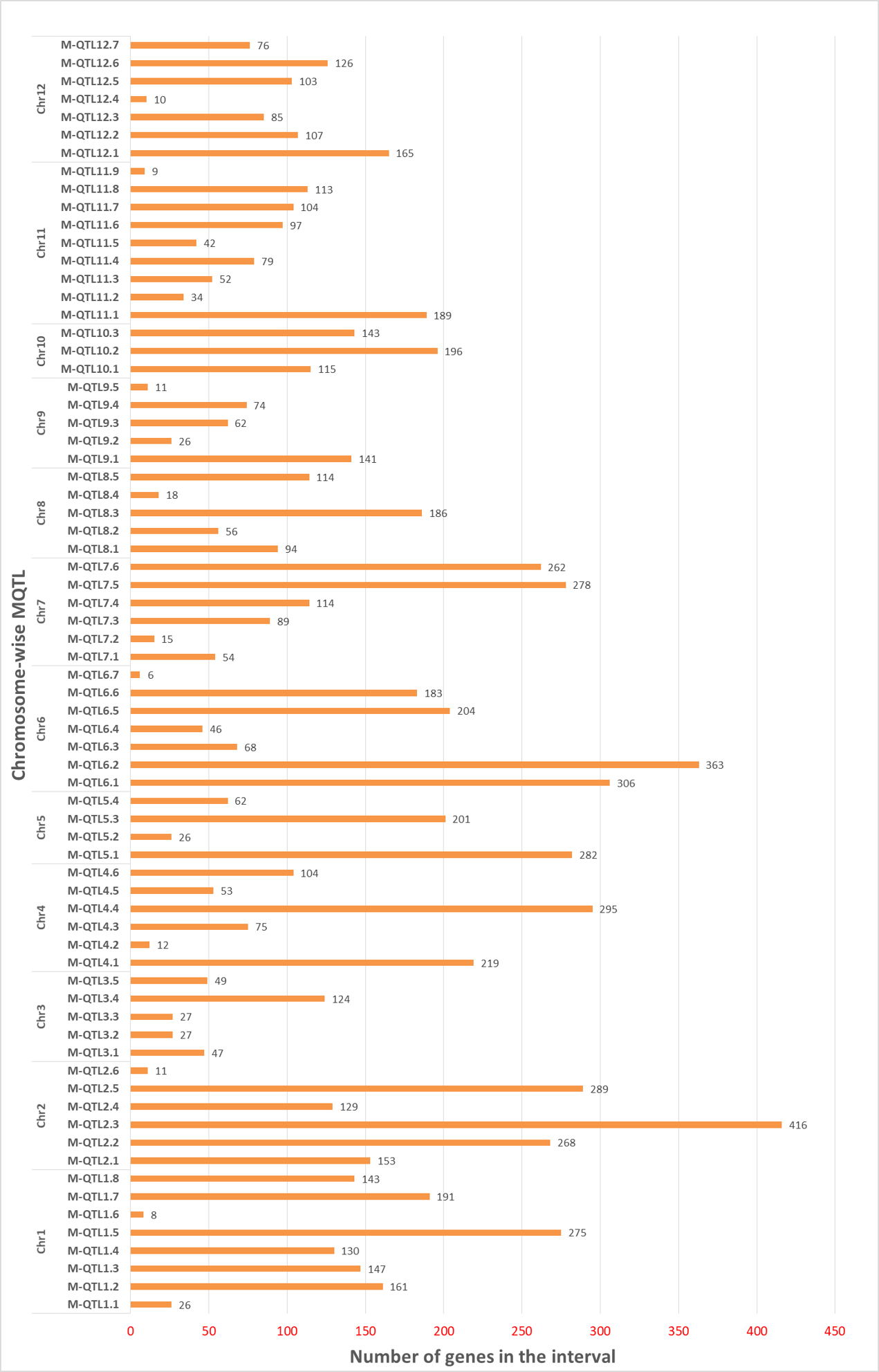


**Fig. S2** Number of genes in the Meta-QTL region across different chromosomes


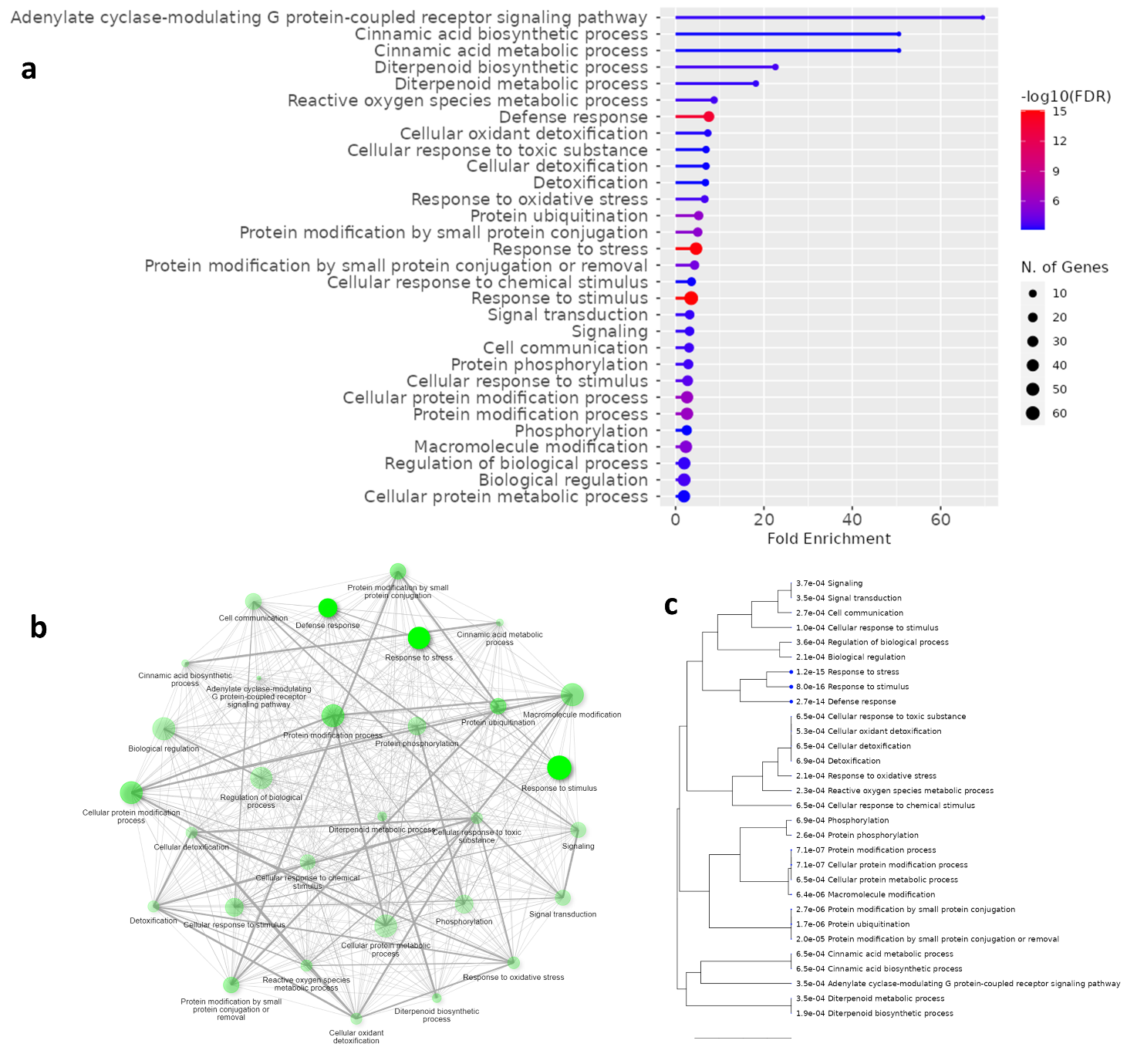


**Fig. S3** Functional annotation of 199 genes related to rice blast resistance. (a) Representation of the number of genes involved in different functional annotation (domain/motif/pathway) with fold enrichment (b) Network of the functions in which these genes are involved (c) Hierarchical clustering based on the number of shared genes


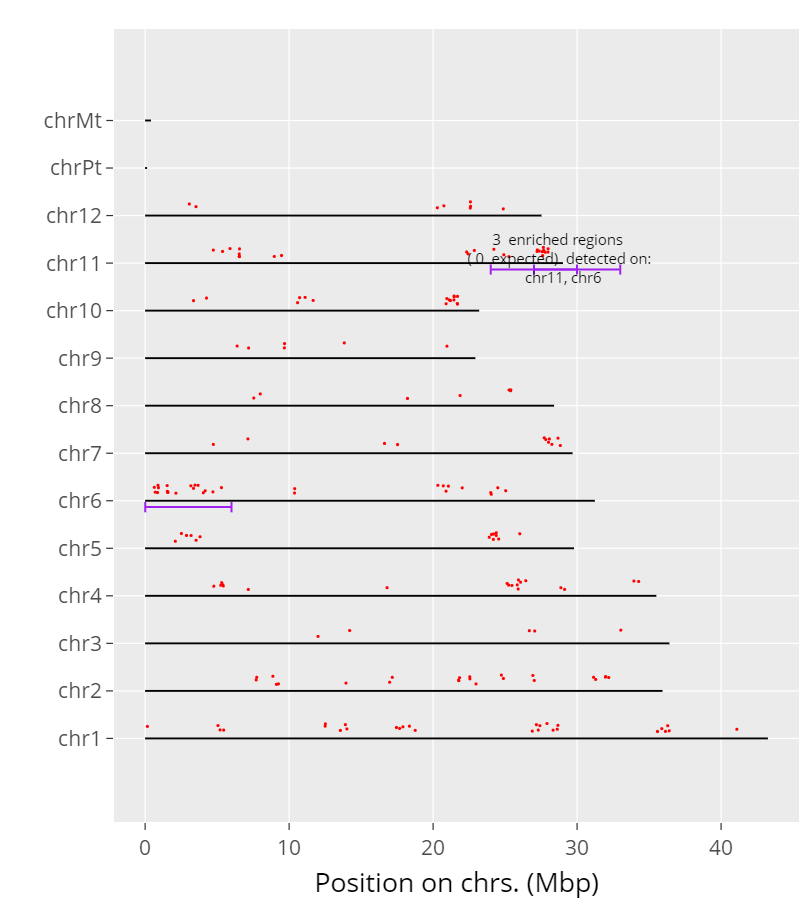


**Fig. S4** Genome-wide representation of fold enrichment of characterised blast resistance genes and characterised RGA.


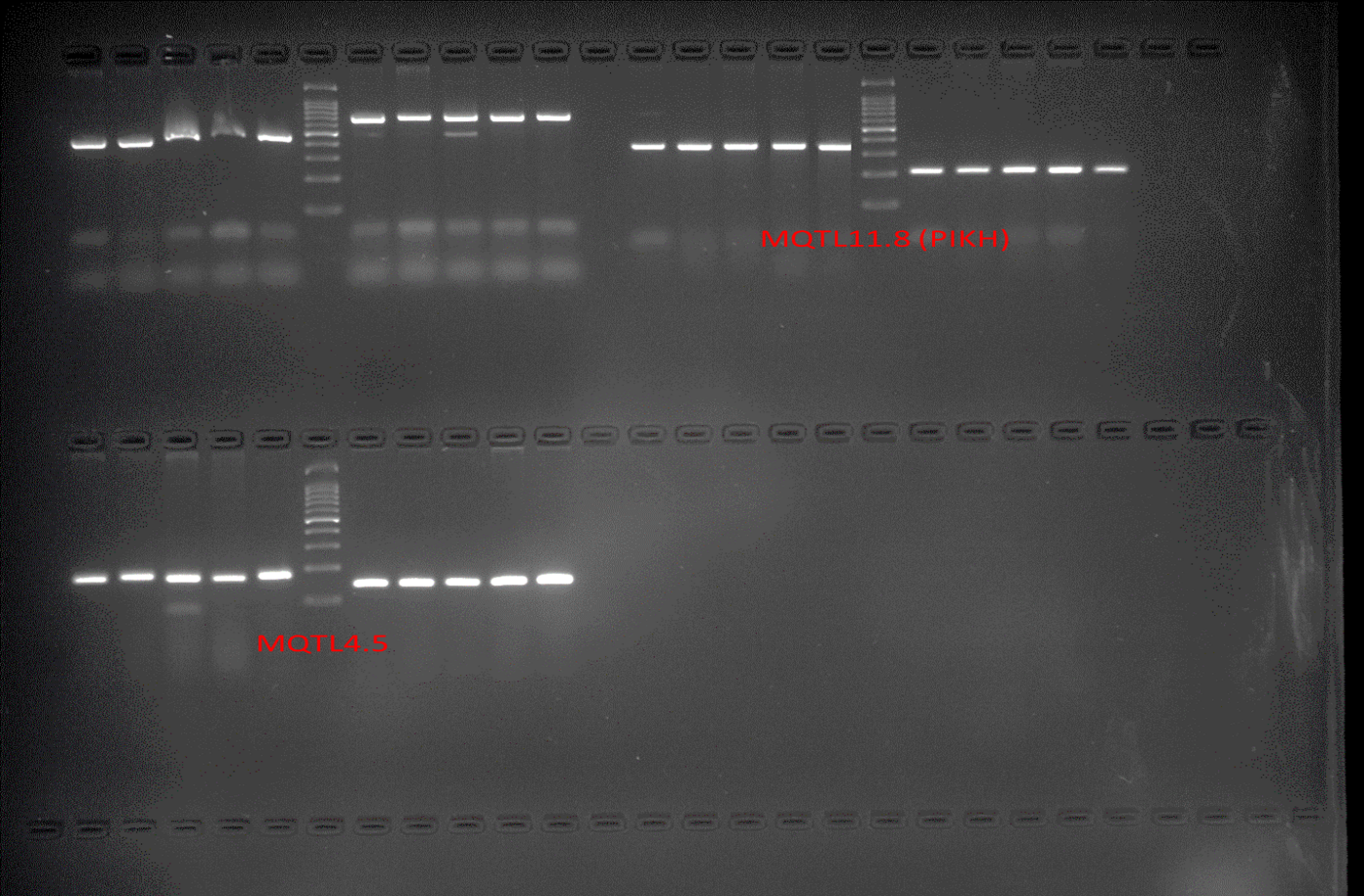


**
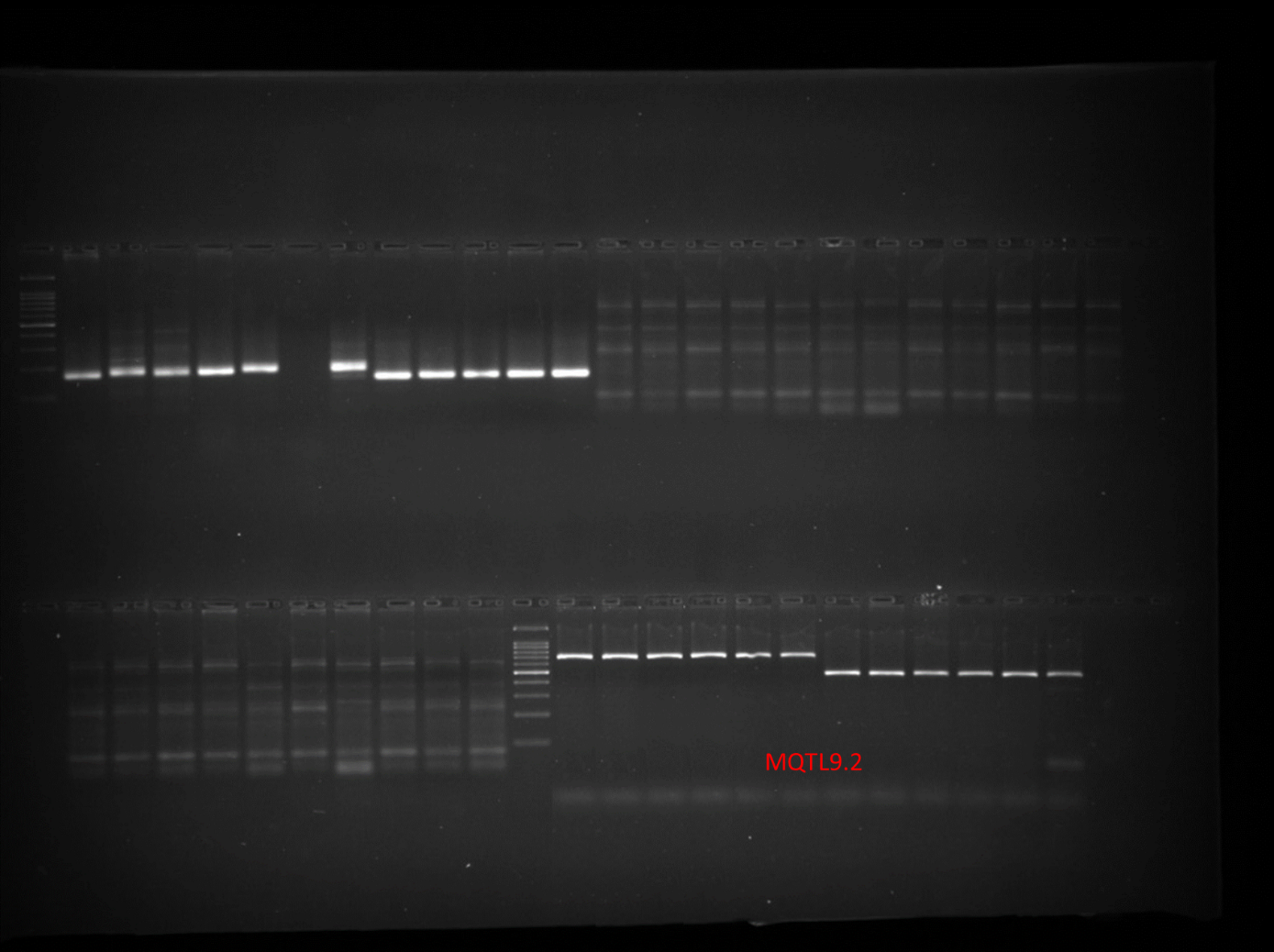
**

**Fig. S5:** Polymorphism between the five blast disease resistant (R) and susceptible (S) contrasts for the peak markers *PIKH* (MQTL11.8), RM17377 (MQTL4.5) and 40N23r (MQTL9.2).


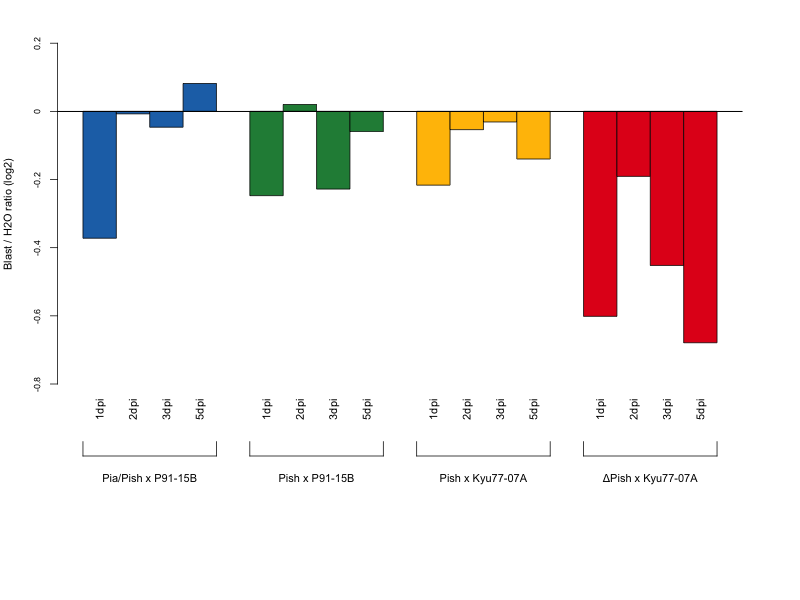
**Fig. S6(I)**: Graphical representation of fold-change in expression of Os01g0102800 gene at 1, 2, 3 and 5- days post-inoculation (dpi) with two *M. oryzae* strains from RiceXPro. Treatments, Pia/Pish × P91-15B and PISH × Kyu77-07A depict incompatible (Resistant) reaction and Pish × P91-15B and ∆PISH × Kyu77-07A depict compatible (Susceptible) reaction. P91-15B and Kyu77-07A are *M. oryzae* strains while Pia, Pish and ∆PISH represent Nipponbare (NB) genotypes carrying respective genes (*Pia* and *Pish*).


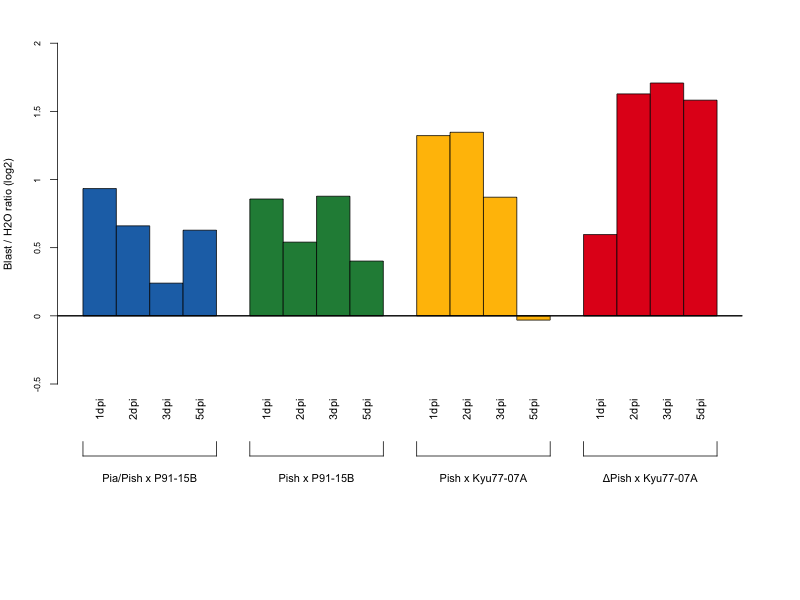
**Fig. S6(II)**: Graphical representation of fold-change in expression of Os01g0194300 gene at 1, 2, 3 and 5- days post-inoculation (dpi) with two *M. oryzae* strains from RiceXPro. Treatments, Pia/Pish × P91-15B and PISH × Kyu77-07A depict incompatible (Resistant) reaction and Pish × P91-15B and ∆PISH × Kyu77-07A depict compatible (Susceptible) reaction. P91-15B and Kyu77-07A are *M. oryzae* strains while Pia, Pish and ∆PISH represent Nipponbare (NB) genotypes carrying respective genes (*Pia* and *Pish*).


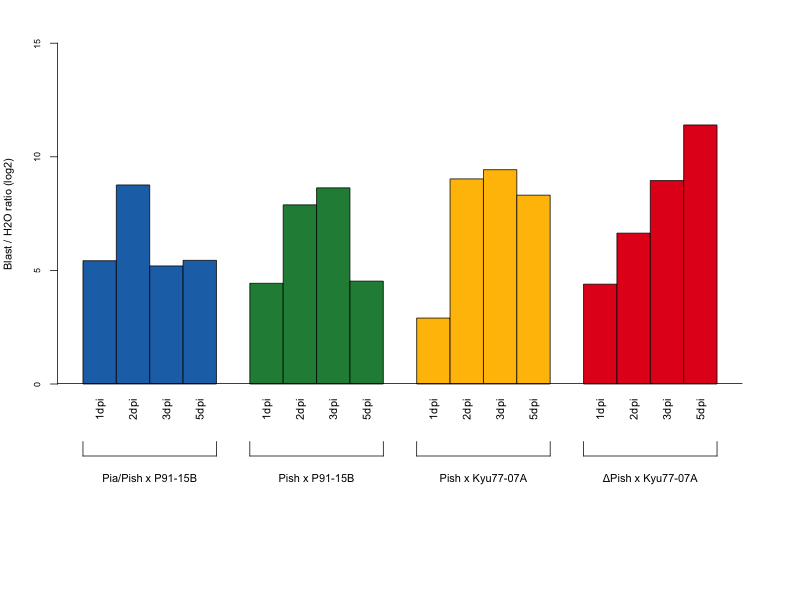
 **Fig. S6(III)**: Graphical representation of fold-change in expression of Os01g0196300 gene at 1, 2, 3 and 5- days post-inoculation (dpi) with two *M. oryzae* strains from RiceXPro. Treatments, Pia/Pish × P91-15B and PISH × Kyu77-07A depict incompatible (Resistant) reaction and Pish × P91-15B and ∆PISH × Kyu77-07A depict compatible (Susceptible) reaction. P91-15B and Kyu77-07A are *M. oryzae* strains while Pia, Pish and ∆PISH represent Nipponbare (NB) genotypes carrying respective genes (*Pia* and *Pish*).


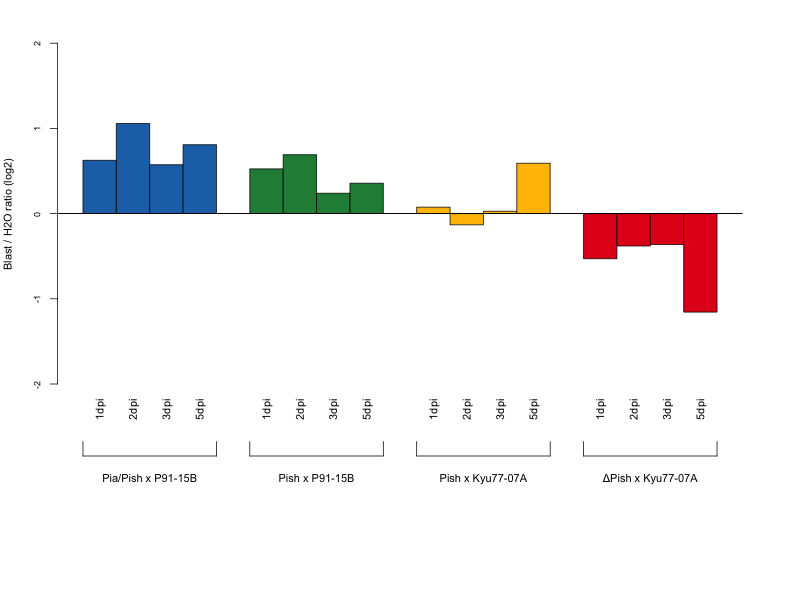


**Fig. S6(IV)**: Graphical representation of fold-change in expression of Os01g0326000 gene at 1, 2, 3 and 5- days post-inoculation (dpi) with two *M. oryzae* strains from RiceXPro. Treatments, Pia/Pish × P91-15B and PISH × Kyu77-07A depict incompatible (Resistant) reaction and Pish × P91-15B and ∆PISH × Kyu77-07A depict compatible (Susceptible) reaction. P91-15B and Kyu77-07A are *M. oryzae* strains while Pia, Pish and ∆PISH represent Nipponbare (NB) genotypes carrying respective genes (*Pia* and *Pish*).


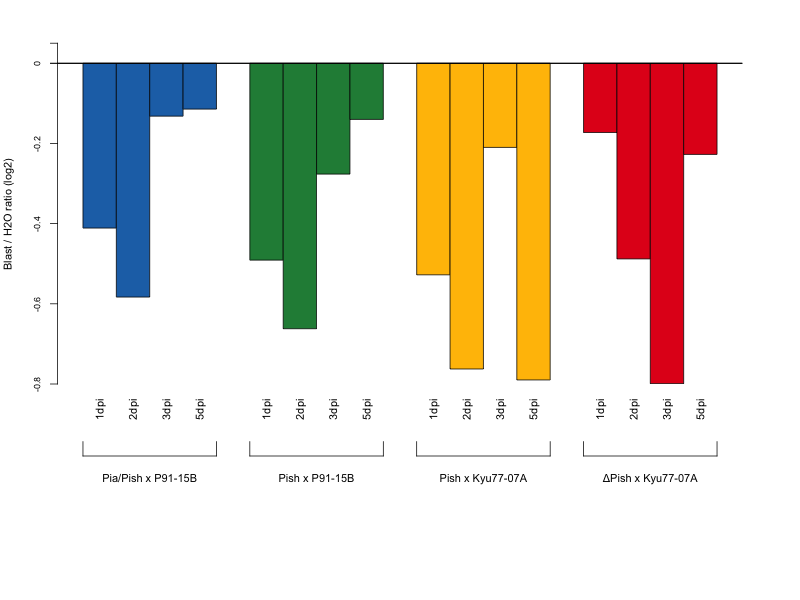


**Fig. S6(V)**: Graphical representation of fold-change in expression of Os01g0343200 gene at 1, 2, 3 and 5- days post-inoculation (dpi) with two *M. oryzae* strains from RiceXPro. Treatments, Pia/Pish × P91-15B and PISH × Kyu77-07A depict incompatible (Resistant) reaction and Pish × P91-15B and ∆PISH × Kyu77-07A depict compatible (Susceptible) reaction. P91-15B and Kyu77-07A are *M. oryzae* strains while Pia, Pish and ∆PISH represent Nipponbare (NB) genotypes carrying respective genes (*Pia* and *Pish*).


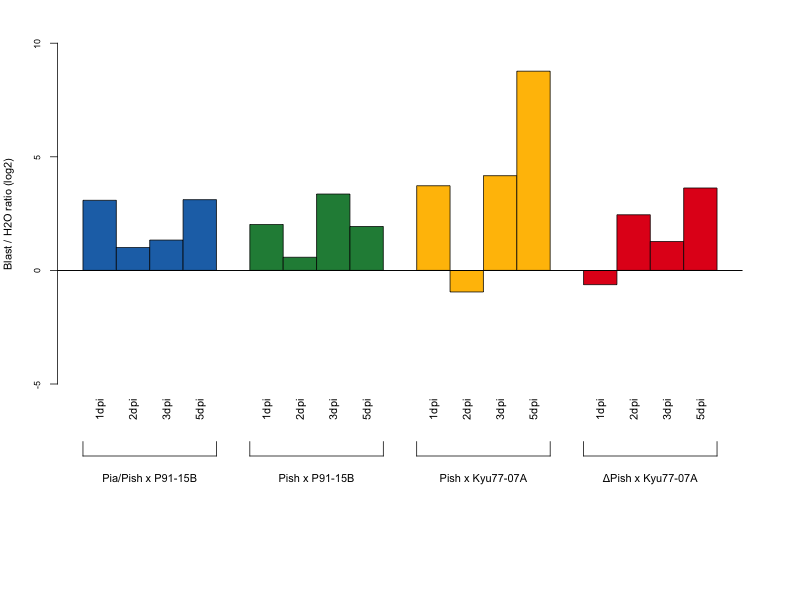
 **Fig. S6(VI)**: Graphical representation of fold-change in expression of Os01g0348900 gene at 1, 2, 3 and 5- days post-inoculation (dpi) with two *M. oryzae* strains from RiceXPro. Treatments, Pia/Pish × P91-15B and PISH × Kyu77-07A depict incompatible (Resistant) reaction and Pish × P91-15B and ∆PISH × Kyu77-07A depict compatible (Susceptible) reaction. P91-15B and Kyu77-07A are *M. oryzae* strains while Pia, Pish and ∆PISH represent Nipponbare (NB) genotypes carrying respective genes (*Pia* and *Pish*).


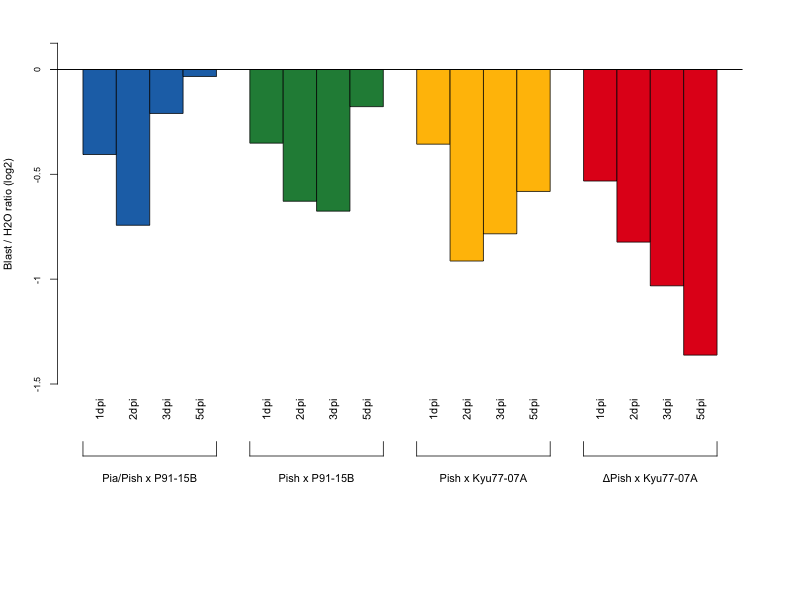
 **Fig. S6(VII)**: Graphical representation of fold-change in expression of Os01g0350900 gene at 1, 2, 3 and 5- days post-inoculation (dpi) with two *M. oryzae* strains from RiceXPro. Treatments, Pia/Pish × P91-15B and PISH × Kyu77-07A depict incompatible (Resistant) reaction and Pish × P91-15B and ∆PISH × Kyu77-07A depict compatible (Susceptible) reaction. P91-15B and Kyu77-07A are *M. oryzae* strains while Pia, Pish and ∆PISH represent Nipponbare (NB) genotypes carrying respective genes (*Pia* and *Pish*).

Os01g0350900


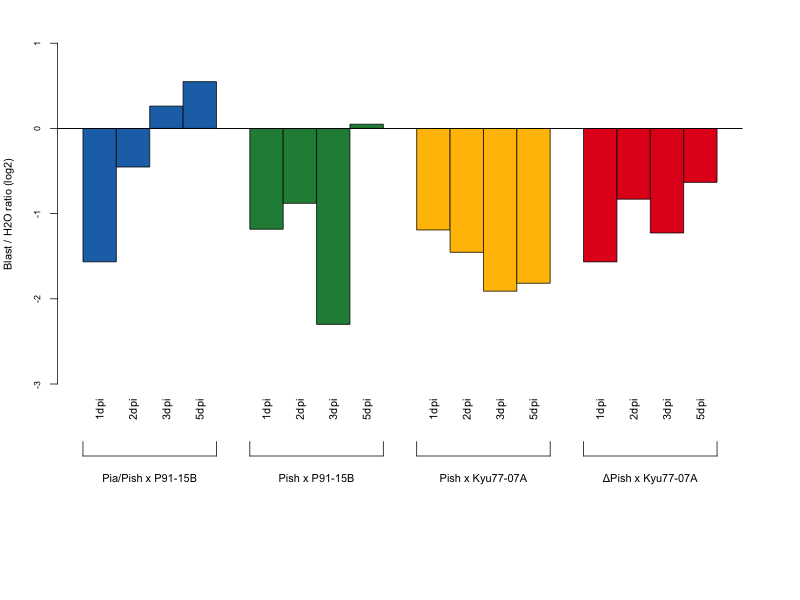
 **Fig. S6(VIII)**: Graphical representation of fold-change in expression of Os01g0503400 gene at 1, 2, 3 and 5- days post-inoculation (dpi) with two *M. oryzae* strains from RiceXPro. Treatments, Pia/Pish × P91-15B and PISH × Kyu77-07A depict incompatible (Resistant) reaction and Pish × P91-15B and ∆PISH × Kyu77-07A depict compatible (Susceptible) reaction. P91-15B and Kyu77-07A are *M. oryzae* strains while Pia, Pish and ∆PISH represent Nipponbare (NB) genotypes carrying respective genes (*Pia* and *Pish*).

Os01g0503400


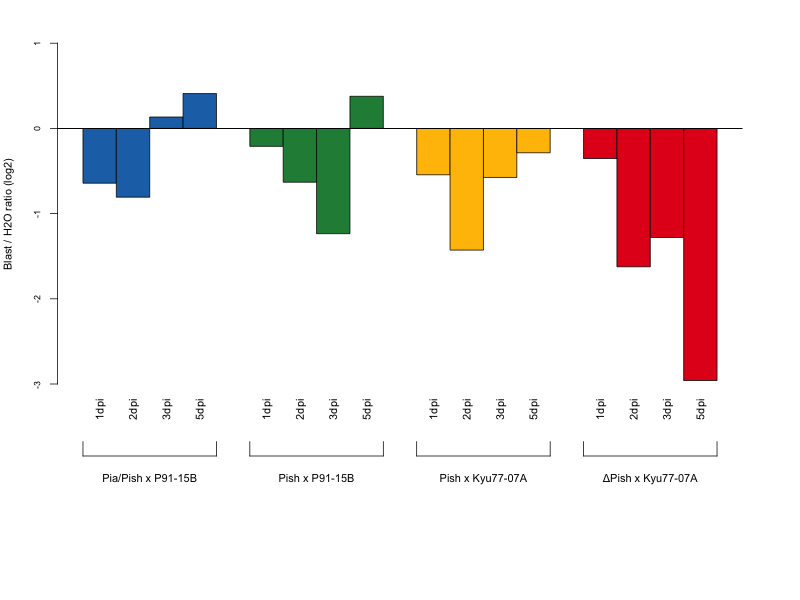
 **Fig. S6(IX)**: Graphical representation of fold-change in expression of Os01g0507000 gene at 1, 2, 3 and 5- days post-inoculation (dpi) with two *M. oryzae* strains from RiceXPro. Treatments, Pia/Pish × P91-15B and PISH × Kyu77-07A depict incompatible (Resistant) reaction and Pish × P91-15B and ∆PISH × Kyu77-07A depict compatible (Susceptible) reaction. P91-15B and Kyu77-07A are *M. oryzae* strains while Pia, Pish and ∆PISH represent Nipponbare (NB) genotypes carrying respective genes (*Pia* and *Pish*).

Os01g0507000


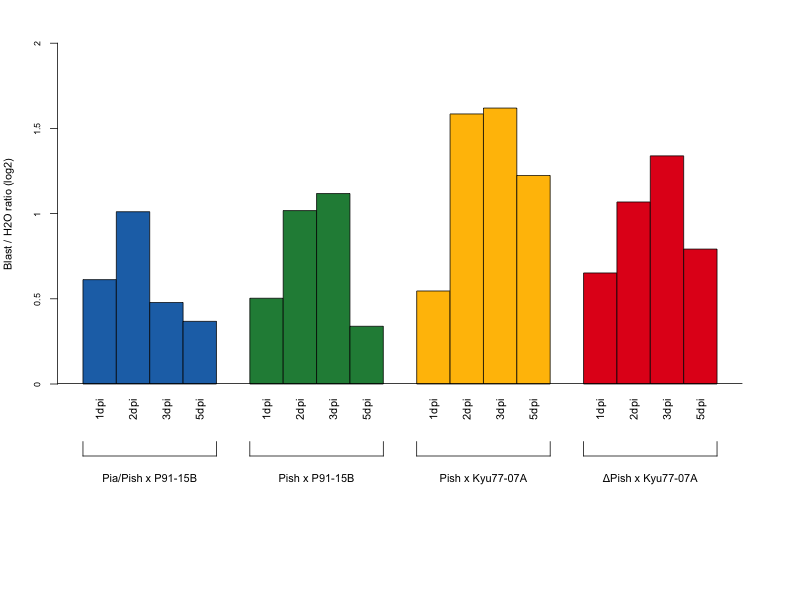
 **Fig. S6(X)**: Graphical representation of fold-change in expression of Os01g0510100

gene at 1, 2, 3 and 5- days post-inoculation (dpi) with two *M. oryzae* strains from RiceXPro. Treatments, Pia/Pish × P91-15B and PISH × Kyu77-07A depict incompatible (Resistant) reaction and Pish × P91-15B and ∆PISH × Kyu77-07A depict compatible (Susceptible) reaction. P91-15B and Kyu77-07A are *M. oryzae* strains while Pia, Pish and ∆PISH represent Nipponbare (NB) genotypes carrying respective genes (*Pia* and *Pish*).

Os01g0510100


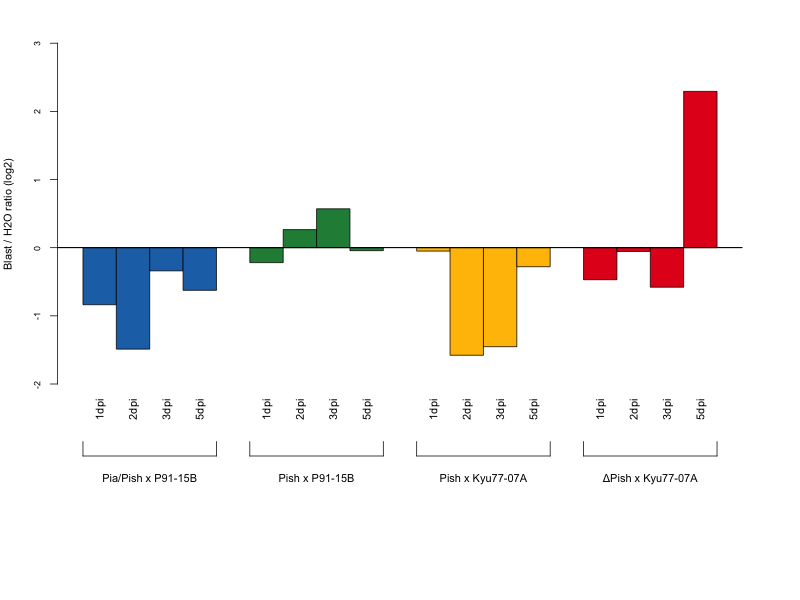
 **Fig. S6(XI)**: Graphical representation of fold-change in expression of Os01g0517500

gene at 1, 2, 3 and 5- days post-inoculation (dpi) with two *M. oryzae* strains from RiceXPro. Treatments, Pia/Pish × P91-15B and PISH × Kyu77-07A depict incompatible (Resistant) reaction and Pish × P91-15B and ∆PISH × Kyu77-07A depict compatible (Susceptible) reaction. P91-15B and Kyu77-07A are *M. oryzae* strains while Pia, Pish and ∆PISH represent Nipponbare (NB) genotypes carrying respective genes (*Pia* and *Pish*).

Os01g0517500


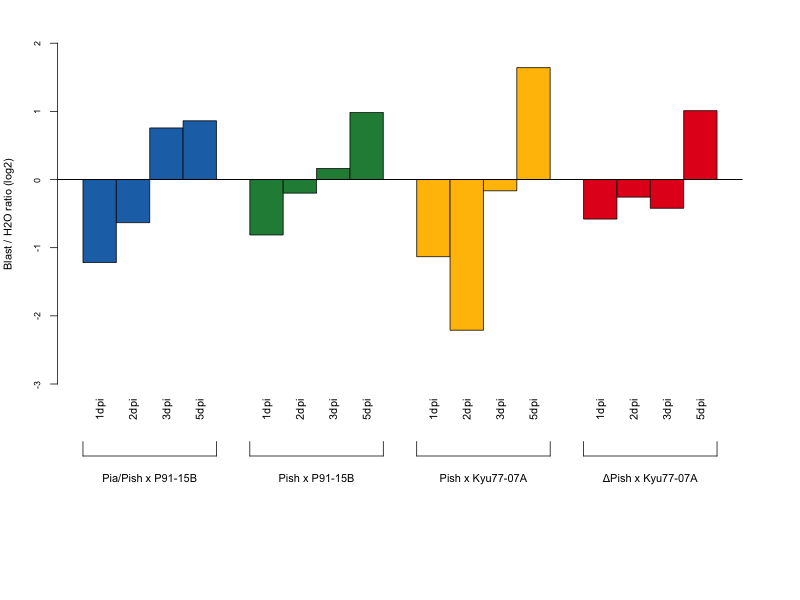
 **Fig. S6(XII)**: Graphical representation of fold-change in expression of Os01g0524500 gene at 1, 2, 3 and 5- days post-inoculation (dpi) with two *M. oryzae* strains from RiceXPro. Treatments, Pia/Pish × P91-15B and PISH × Kyu77-07A depict incompatible (Resistant) reaction and Pish × P91-15B and ∆PISH × Kyu77-07A depict compatible (Susceptible) reaction. P91-15B and Kyu77-07A are *M. oryzae* strains while Pia, Pish and ∆PISH represent Nipponbare (NB) genotypes carrying respective genes (*Pia* and *Pish*).

Os01g0524500


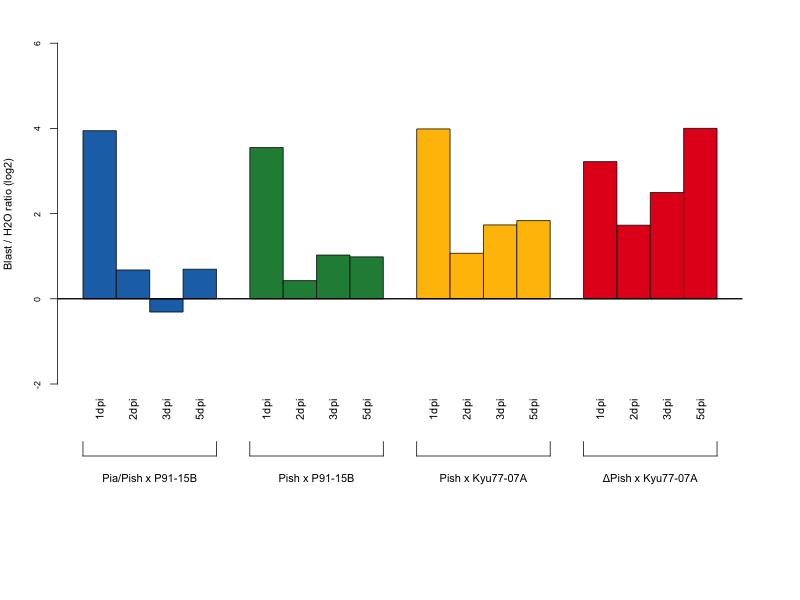
 **Fig. S6(XIII)**: Graphical representation of fold-change in expression of Os01g0660200 gene at 1, 2, 3 and 5- days post-inoculation (dpi) with two *M. oryzae* strains from RiceXPro. Treatments, Pia/Pish × P91-15B and PISH × Kyu77-07A depict incompatible (Resistant) reaction and Pish × P91-15B and ∆PISH × Kyu77-07A depict compatible (Susceptible) reaction. P91-15B and Kyu77-07A are *M. oryzae* strains while Pia, Pish and ∆PISH represent Nipponbare (NB) genotypes carrying respective genes (*Pia* and *Pish*).

Os01g0660200


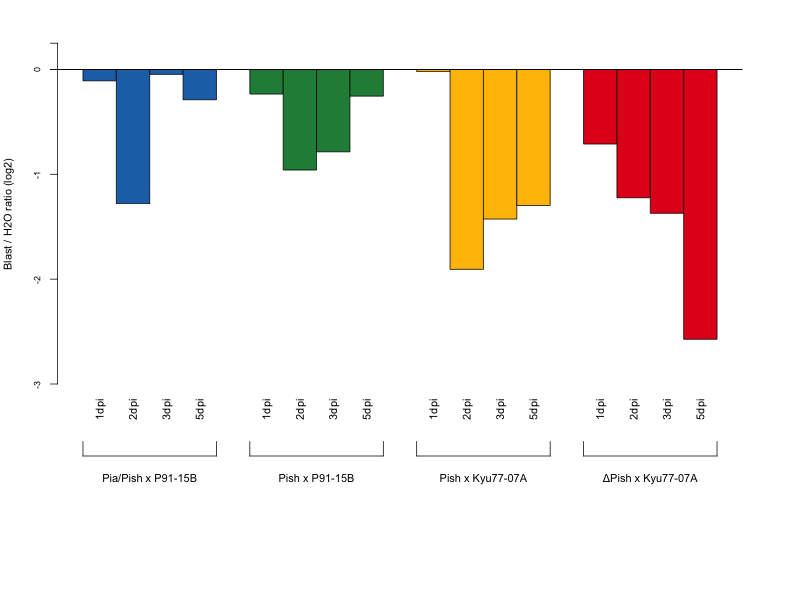
 **Fig. S6(XIV)**: Graphical representation of fold-change in expression of Os01g0665200 gene at 1, 2, 3 and 5- days post-inoculation (dpi) with two *M. oryzae* strains from RiceXPro. Treatments, Pia/Pish × P91-15B and PISH × Kyu77-07A depict incompatible (Resistant) reaction and Pish × P91-15B and ∆PISH × Kyu77-07A depict compatible (Susceptible) reaction. P91-15B and Kyu77-07A are *M. oryzae* strains while Pia, Pish and ∆PISH represent Nipponbare (NB) genotypes carrying respective genes (*Pia* and *Pish*).

Os01g0665200


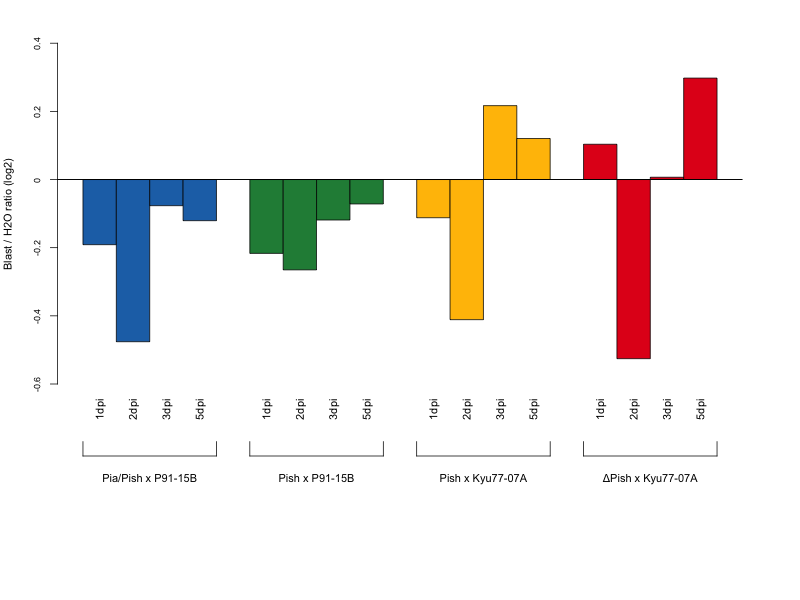
**Fig. S6(XV)**: Graphical representation of fold-change in expression of Os01g0667700 gene at 1, 2, 3 and 5- days post-inoculation (dpi) with two *M. oryzae* strains from RiceXPro. Treatments, Pia/Pish × P91-15B and PISH × Kyu77-07A depict incompatible (Resistant) reaction and Pish × P91-15B and ∆PISH × Kyu77-07A depict compatible (Susceptible) reaction. P91-15B and Kyu77-07A are *M. oryzae* strains while Pia, Pish and ∆PISH represent Nipponbare (NB) genotypes carrying respective genes (*Pia* and *Pish*).

Os01g0667700


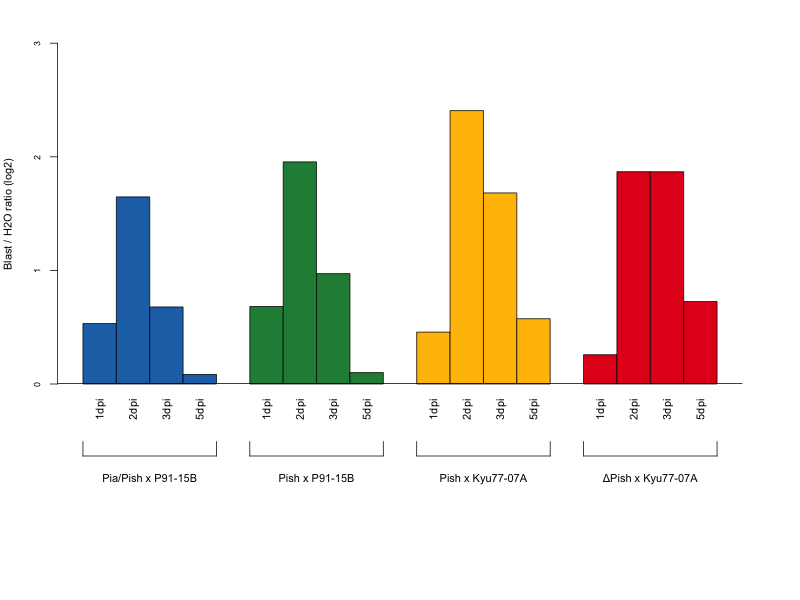
**Fig. S6(XVI)**: Graphical representation of fold-change in expression of Os01g0686800 gene at 1, 2, 3 and 5- days post-inoculation (dpi) with two *M. oryzae* strains from RiceXPro. Treatments, Pia/Pish × P91-15B and PISH × Kyu77-07A depict incompatible (Resistant) reaction and Pish × P91-15B and ∆PISH × Kyu77-07A depict compatible (Susceptible) reaction. P91-15B and Kyu77-07A are *M. oryzae* strains while Pia, Pish and ∆PISH represent Nipponbare (NB) genotypes carrying respective genes (*Pia* and *Pish*).

Os01g0686800


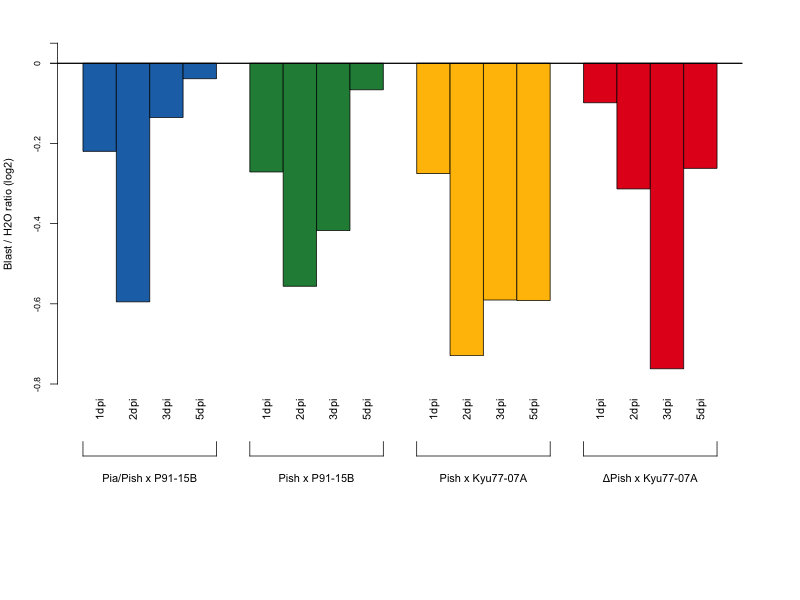
 **Fig. S6(XVII)**: Graphical representation of fold-change in expression of Os01g0831200 gene at 1, 2, 3 and 5- days post-inoculation (dpi) with two *M. oryzae* strains from RiceXPro. Treatments, Pia/Pish × P91-15B and PISH × Kyu77-07A depict incompatible (Resistant) reaction and Pish × P91-15B and ∆PISH × Kyu77-07A depict compatible (Susceptible) reaction. P91-15B and Kyu77-07A are *M. oryzae* strains while Pia, Pish and ∆PISH represent Nipponbare (NB) genotypes carrying respective genes (*Pia* and *Pish*).

Os01g0831200


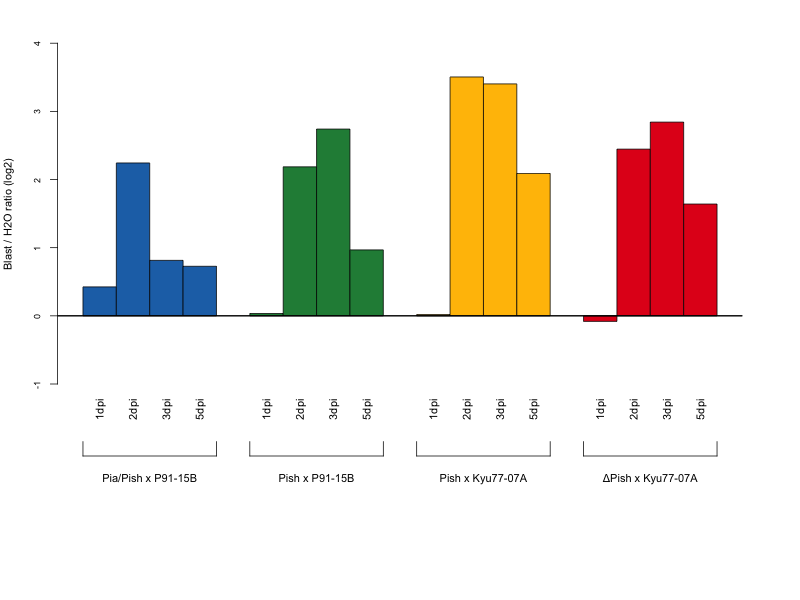
 **Fig. S6(XVIII)**: Graphical representation of fold-change in expression of Os01g0837000 gene at 1, 2, 3 and 5- days post-inoculation (dpi) with two *M. oryzae* strains from RiceXPro. Treatments, Pia/Pish × P91-15B and PISH × Kyu77-07A depict incompatible (Resistant) reaction and Pish × P91-15B and ∆PISH × Kyu77-07A depict compatible (Susceptible) reaction. P91-15B and Kyu77-07A are *M. oryzae* strains while Pia, Pish and ∆PISH represent Nipponbare (NB) genotypes carrying respective genes (*Pia* and *Pish*).

Os01g0837000


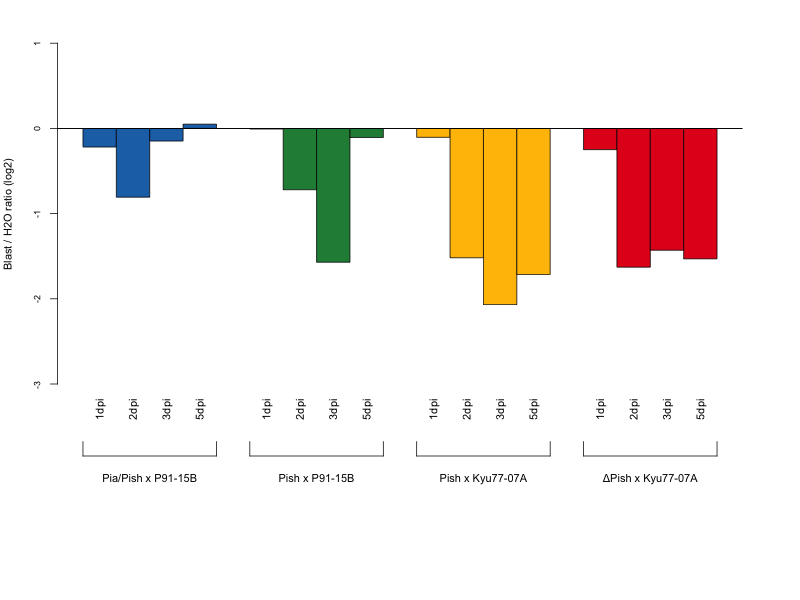
 **Fig. S6(XIX)**: Graphical representation of fold-change in expression of Os01g0847200 gene at 1, 2, 3 and 5- days post-inoculation (dpi) with two *M. oryzae* strains from RiceXPro. Treatments, Pia/Pish × P91-15B and PISH × Kyu77-07A depict incompatible (Resistant) reaction and Pish × P91-15B and ∆PISH × Kyu77-07A depict compatible (Susceptible) reaction. P91-15B and Kyu77-07A are *M. oryzae* strains while Pia, Pish and ∆PISH represent Nipponbare (NB) genotypes carrying respective genes (*Pia* and *Pish*).

Os01g0847200


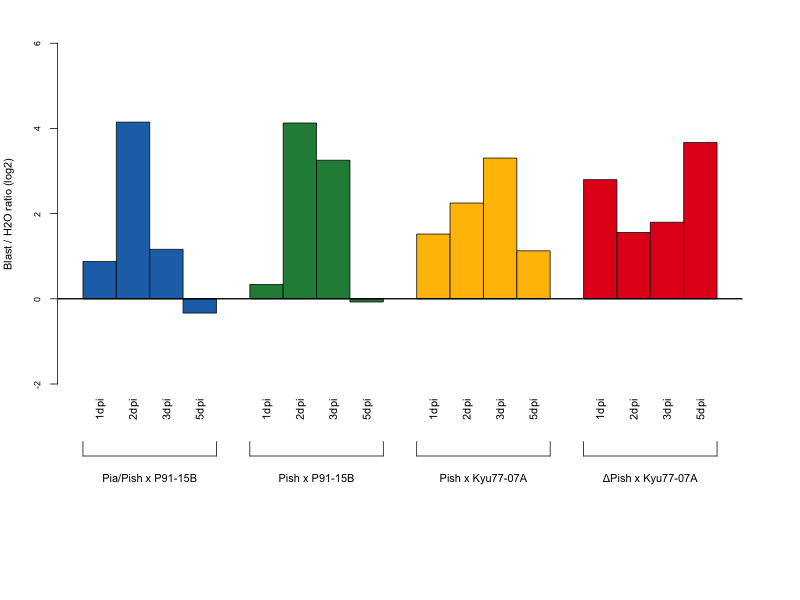
 **Fig. S6(XX)**: Graphical representation of fold-change in expression of Os01g0934800 gene at 1, 2, 3 and 5- days post-inoculation (dpi) with two *M. oryzae* strains from RiceXPro. Treatments, Pia/Pish × P91-15B and PISH × Kyu77-07A depict incompatible (Resistant) reaction and Pish × P91-15B and ∆PISH × Kyu77-07A depict compatible (Susceptible) reaction. P91-15B and Kyu77-07A are *M. oryzae* strains while Pia, Pish and ∆PISH represent Nipponbare (NB) genotypes carrying respective genes (*Pia* and *Pish*).

Os01g0934800


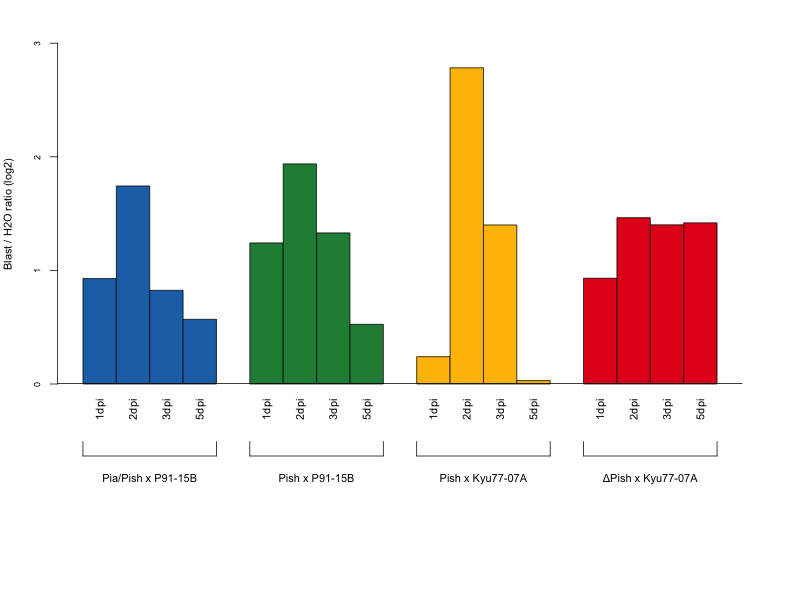
 **Fig. S6(XXI)**: Graphical representation of fold-change in expression of Os01g0934900 gene at 1, 2, 3 and 5- days post-inoculation (dpi) with two *M. oryzae* strains from RiceXPro. Treatments, Pia/Pish × P91-15B and PISH × Kyu77-07A depict incompatible (Resistant) reaction and Pish × P91-15B and ∆PISH × Kyu77-07A depict compatible (Susceptible) reaction. P91-15B and Kyu77-07A are *M. oryzae* strains while Pia, Pish and ∆PISH represent Nipponbare (NB) genotypes carrying respective genes (*Pia* and *Pish*).

Os01g0934900


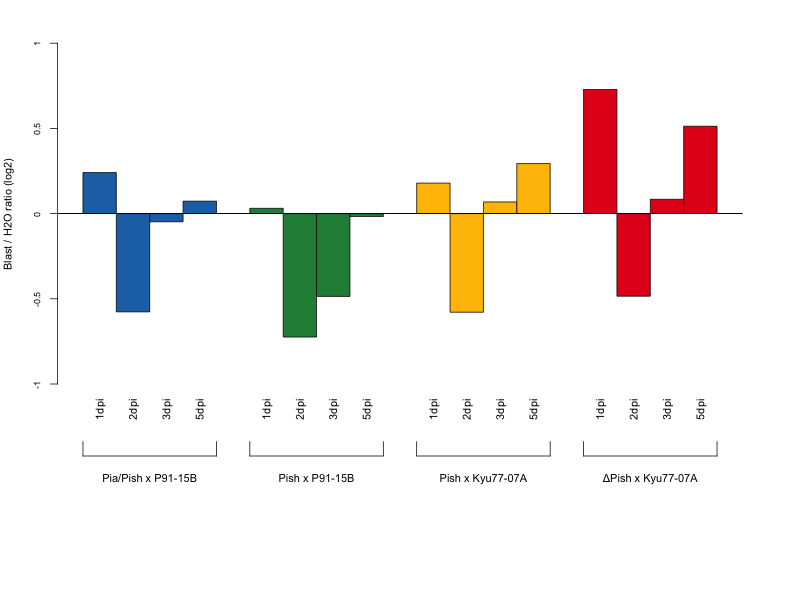
 **Fig. S6(XXII)**: Graphical representation of fold-change in expression of Os01g0936100 gene at 1, 2, 3 and 5- days post-inoculation (dpi) with two *M. oryzae* strains from RiceXPro. Treatments, Pia/Pish × P91-15B and PISH × Kyu77-07A depict incompatible (Resistant) reaction and Pish × P91-15B and ∆PISH × Kyu77-07A depict compatible (Susceptible) reaction. P91-15B and Kyu77-07A are *M. oryzae* strains while Pia, Pish and ∆PISH represent Nipponbare (NB) genotypes carrying respective genes (*Pia* and *Pish*).

Os01g0936100


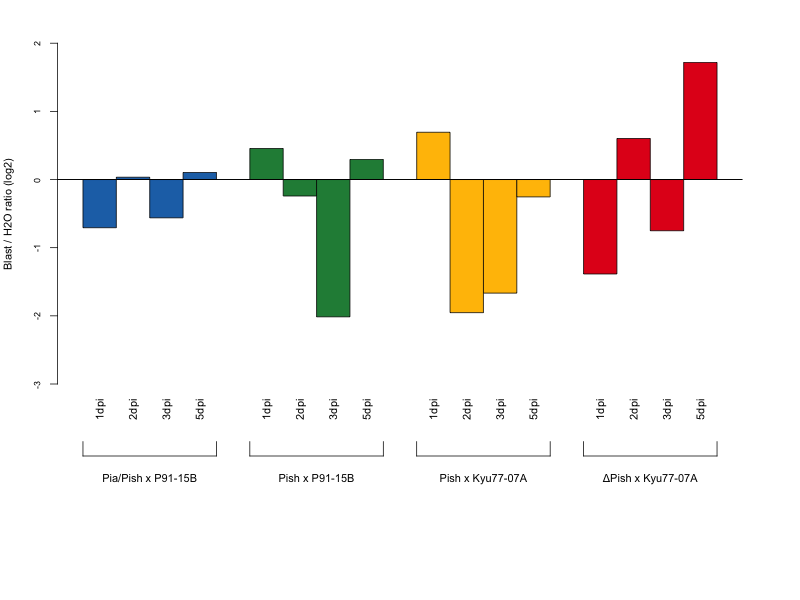
 **Fig. S6(XXIII)**: Graphical representation of fold-change in expression of Os02g0236600 gene at 1, 2, 3 and 5- days post-inoculation (dpi) with two *M. oryzae* strains from RiceXPro. Treatments, Pia/Pish × P91-15B and PISH × Kyu77-07A depict incompatible (Resistant) reaction and Pish × P91-15B and ∆PISH × Kyu77-07A depict compatible (Susceptible) reaction. P91-15B and Kyu77-07A are *M. oryzae* strains while Pia, Pish and ∆PISH represent Nipponbare (NB) genotypes carrying respective genes (*Pia* and *Pish*).

Os02g0236600


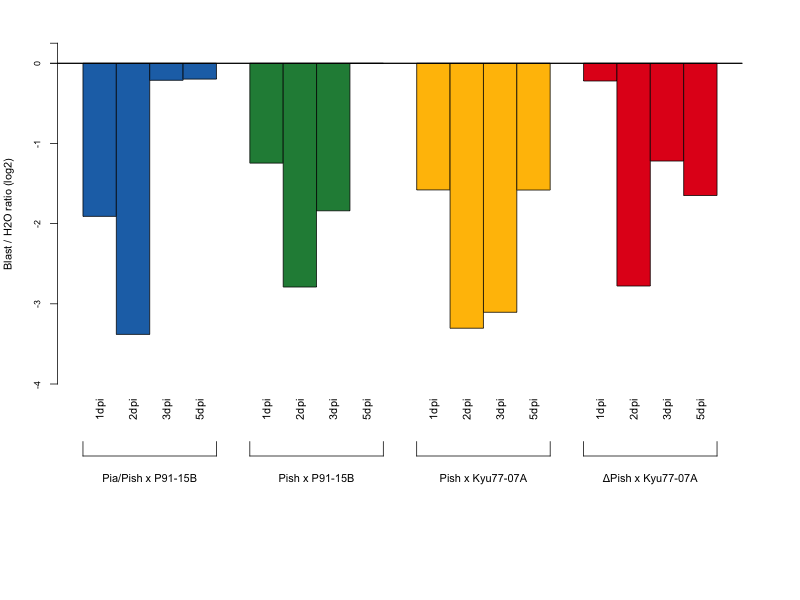
 **Fig. S6(XXIV)**: Graphical representation of fold-change in expression of Os02g0257300 gene at 1, 2, 3 and 5- days post-inoculation (dpi) with two *M. oryzae* strains from RiceXPro. Treatments, Pia/Pish × P91-15B and PISH × Kyu77-07A depict incompatible (Resistant) reaction and Pish × P91-15B and ∆PISH × Kyu77-07A depict compatible (Susceptible) reaction. P91-15B and Kyu77-07A are *M. oryzae* strains while Pia, Pish and ∆PISH represent Nipponbare (NB) genotypes carrying respective genes (*Pia* and *Pish*).


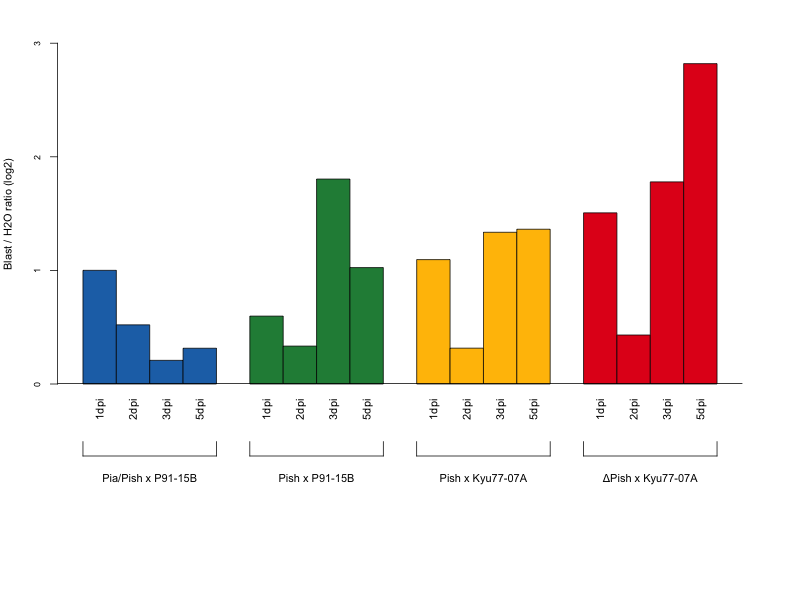
 **Fig. S6(XXV)**: Graphical representation of fold-change in expression of Os02g0437200 gene at 1, 2, 3 and 5- days post-inoculation (dpi) with two *M. oryzae* strains from RiceXPro. Treatments, Pia/Pish × P91-15B and PISH × Kyu77-07A depict incompatible (Resistant) reaction and Pish × P91-15B and ∆PISH × Kyu77-07A depict compatible (Susceptible) reaction. P91-15B and Kyu77-07A are *M. oryzae* strains while Pia, Pish and ∆PISH represent Nipponbare (NB) genotypes carrying respective genes (*Pia* and *Pish*).

Os02g0437200


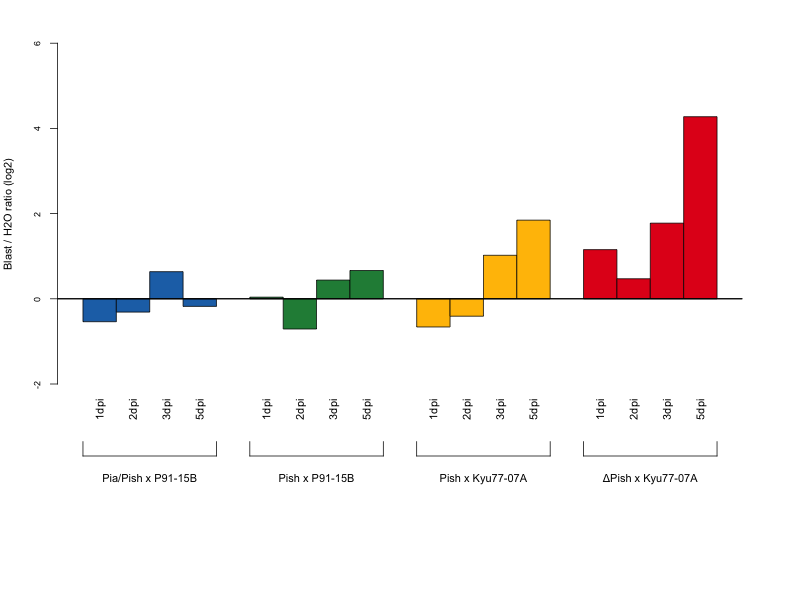
 **Fig. S6(XXVI)**: Graphical representation of fold-change in expression of Os02g0488600 gene at 1, 2, 3 and 5- days post-inoculation (dpi) with two *M. oryzae* strains from RiceXPro. Treatments, Pia/Pish × P91-15B and PISH × Kyu77-07A depict incompatible (Resistant) reaction and Pish × P91-15B and ∆PISH × Kyu77-07A depict compatible (Susceptible) reaction. P91-15B and Kyu77-07A are *M. oryzae* strains while Pia, Pish and ∆PISH represent Nipponbare (NB) genotypes carrying respective genes (*Pia* and *Pish*).

Os02g0488600


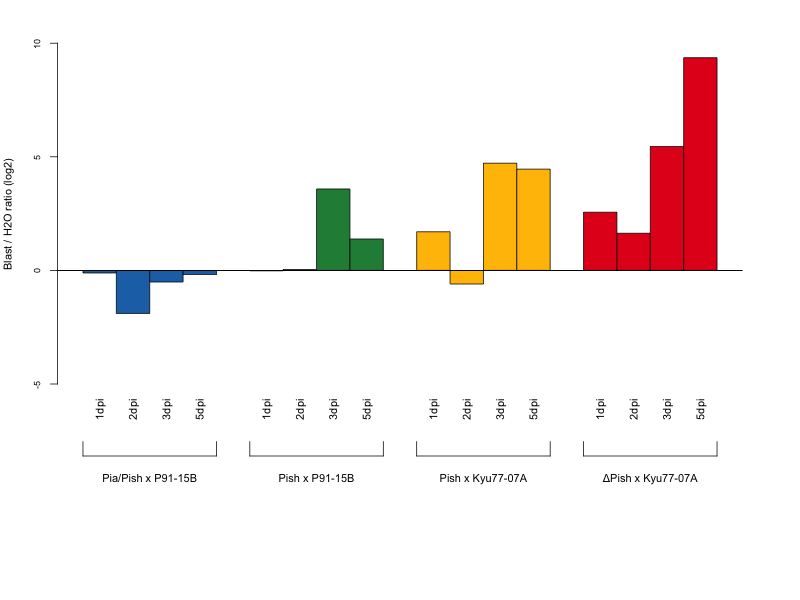
 **Fig. S6(XXVII)**: Graphical representation of fold-change in expression of Os02g0491600 gene at 1, 2, 3 and 5- days post-inoculation (dpi) with two *M. oryzae* strains from RiceXPro. Treatments, Pia/Pish × P91-15B and PISH × Kyu77-07A depict incompatible (Resistant) reaction and Pish × P91-15B and ∆PISH × Kyu77-07A depict compatible (Susceptible) reaction. P91-15B and Kyu77-07A are *M. oryzae* strains while Pia, Pish and ∆PISH represent Nipponbare (NB) genotypes carrying respective genes (*Pia* and *Pish*).

Os02g0491600


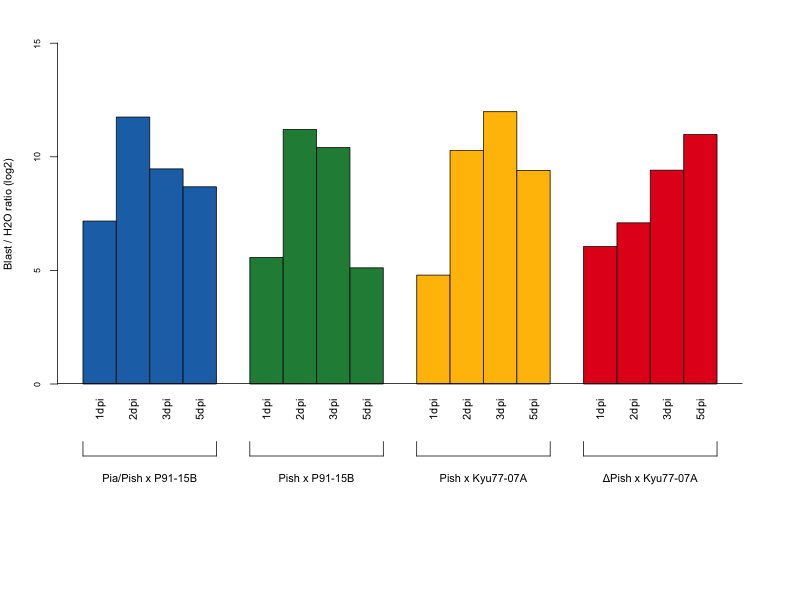
 **Fig. S6(XXVIII)**: Graphical representation of fold-change in expression of Os02g0570400 gene at 1, 2, 3 and 5- days post-inoculation (dpi) with two *M. oryzae* strains from RiceXPro. Treatments, Pia/Pish × P91-15B and PISH × Kyu77-07A depict incompatible (Resistant) reaction and Pish × P91-15B and ∆PISH × Kyu77-07A depict compatible (Susceptible) reaction. P91-15B and Kyu77-07A are *M. oryzae* strains while Pia, Pish and ∆PISH represent Nipponbare (NB) genotypes carrying respective genes (*Pia* and *Pish*).

Os02g0570400


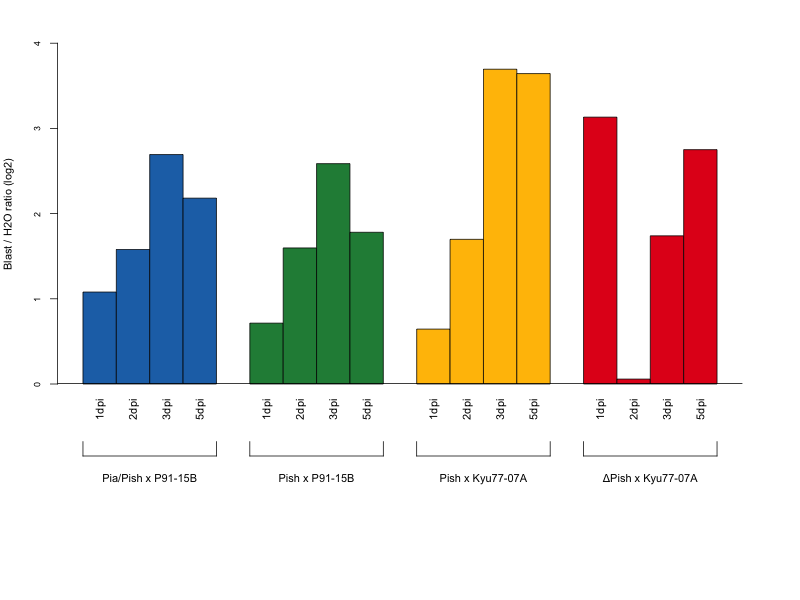
 **Fig. S6(XXIX)**: Graphical representation of fold-change in expression of Os02g0570500 gene at 1, 2, 3 and 5- days post-inoculation (dpi) with two *M. oryzae* strains from RiceXPro. Treatments, Pia/Pish × P91-15B and PISH × Kyu77-07A depict incompatible (Resistant) reaction and Pish × P91-15B and ∆PISH × Kyu77-07A depict compatible (Susceptible) reaction. P91-15B and Kyu77-07A are *M. oryzae* strains while Pia, Pish and ∆PISH represent Nipponbare (NB) genotypes carrying respective genes (*Pia* and *Pish*).

Os02g0570500


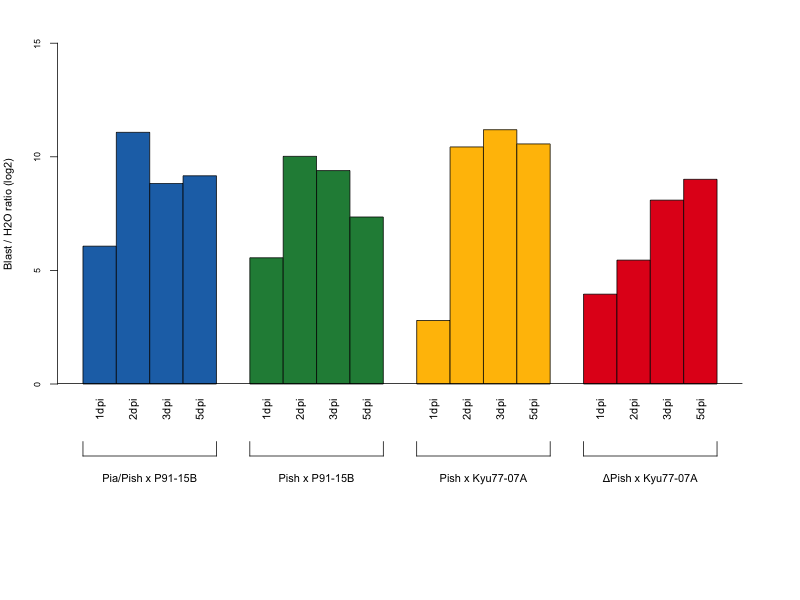
 **Fig. S6(XXX)**: Graphical representation of fold-change in expression of Os02g0571100 gene at 1, 2, 3 and 5- days post-inoculation (dpi) with two *M. oryzae* strains from RiceXPro. Treatments, Pia/Pish × P91-15B and PISH × Kyu77-07A depict incompatible (Resistant) reaction and Pish × P91-15B and ∆PISH × Kyu77-07A depict compatible (Susceptible) reaction. P91-15B and Kyu77-07A are *M. oryzae* strains while Pia, Pish and ∆PISH represent Nipponbare (NB) genotypes carrying respective genes (*Pia* and *Pish*).

Os02g0571100


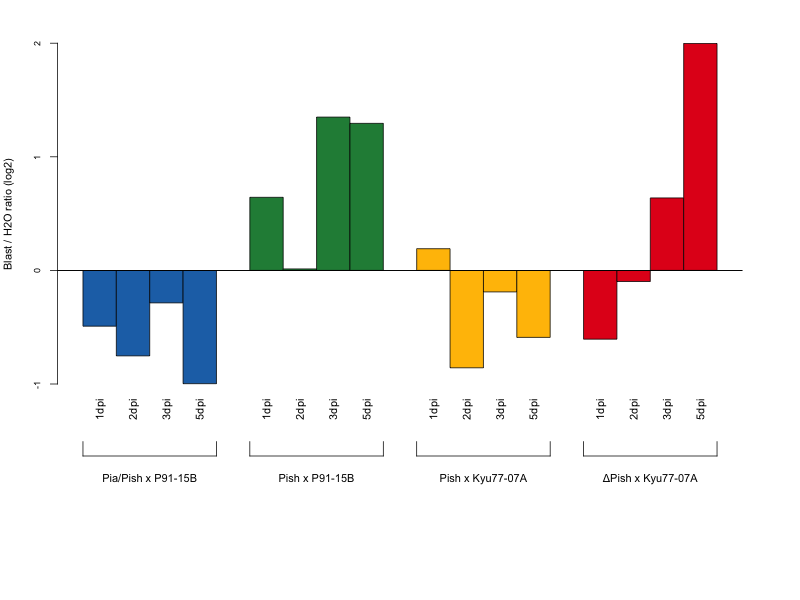
 **Fig. S6(XXXI)**: Graphical representation of fold-change in expression of Os02g0584700 gene at 1, 2, 3 and 5- days post-inoculation (dpi) with two *M. oryzae* strains from RiceXPro. Treatments, Pia/Pish × P91-15B and PISH × Kyu77-07A depict incompatible (Resistant) reaction and Pish × P91-15B and ∆PISH × Kyu77-07A depict compatible (Susceptible) reaction. P91-15B and Kyu77-07A are *M. oryzae* strains while Pia, Pish and ∆PISH represent Nipponbare (NB) genotypes carrying respective genes (*Pia* and *Pish*).

Os02g0584700


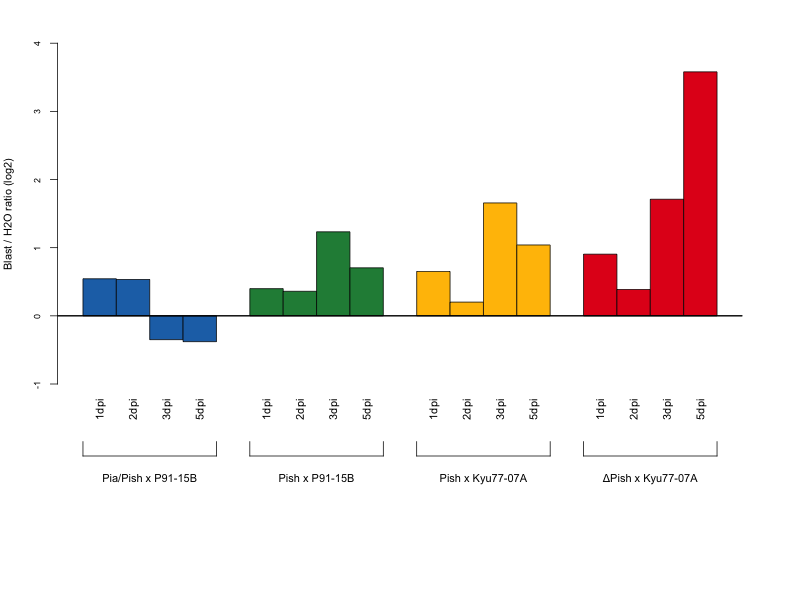
 **Fig. S6(XXXII)**: Graphical representation of fold-change in expression of Os02g0584800 gene at 1, 2, 3 and 5- days post-inoculation (dpi) with two *M. oryzae* strains from RiceXPro. Treatments, Pia/Pish × P91-15B and PISH × Kyu77-07A depict incompatible (Resistant) reaction and Pish × P91-15B and ∆PISH × Kyu77-07A depict compatible (Susceptible) reaction. P91-15B and Kyu77-07A are *M. oryzae* strains while Pia, Pish and ∆PISH represent Nipponbare (NB) genotypes carrying respective genes (*Pia* and *Pish*).

Os02g0584800


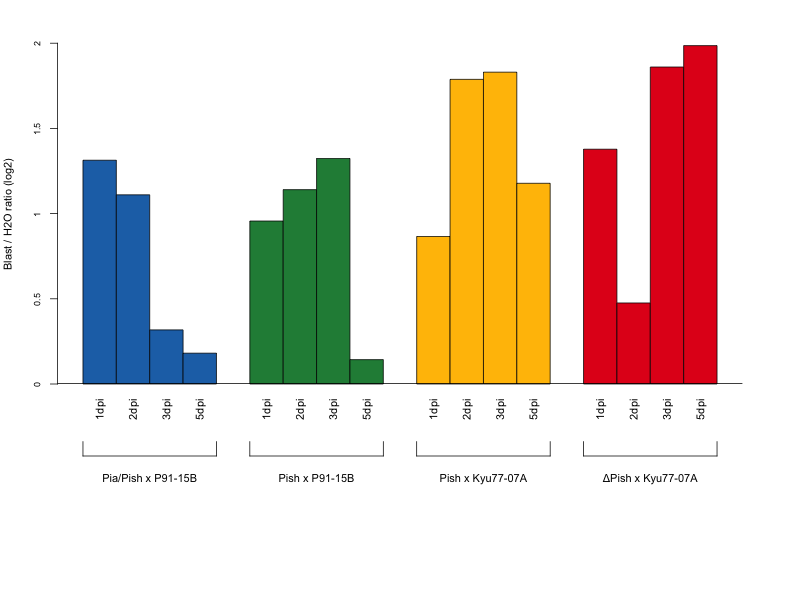
 **Fig. S6(XXXIII)**: Graphical representation of fold-change in expression of Os02g0621700 gene at 1, 2, 3 and 5- days post-inoculation (dpi) with two *M. oryzae* strains from RiceXPro. Treatments, Pia/Pish × P91-15B and PISH × Kyu77-07A depict incompatible (Resistant) reaction and Pish × P91-15B and ∆PISH × Kyu77-07A depict compatible (Susceptible) reaction. P91-15B and Kyu77-07A are *M. oryzae* strains while Pia, Pish and ∆PISH represent Nipponbare (NB) genotypes carrying respective genes (*Pia* and *Pish*).

Os02g0621700


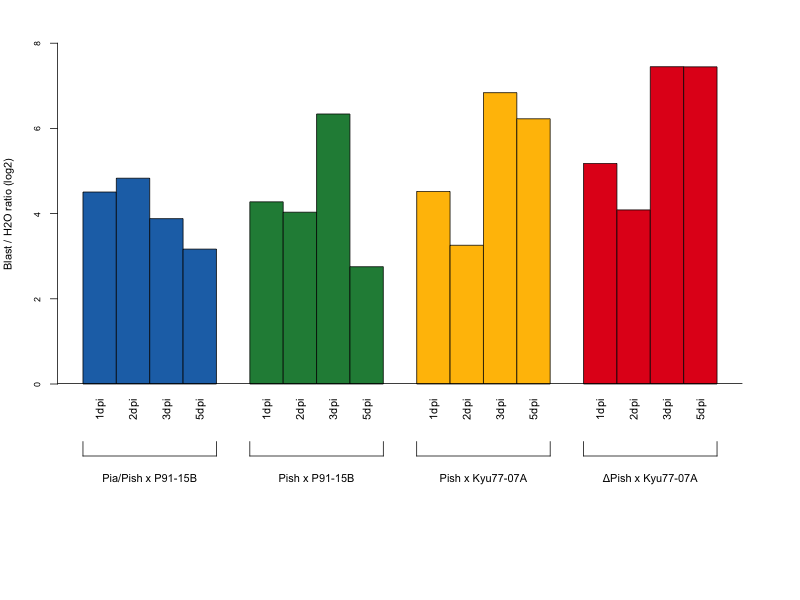
 **Fig. S6(XXXIV)**: Graphical representation of fold-change in expression of Os02g0624300 gene at 1, 2, 3 and 5- days post-inoculation (dpi) with two *M. oryzae* strains from RiceXPro. Treatments, Pia/Pish × P91-15B and PISH × Kyu77-07A depict incompatible (Resistant) reaction and Pish × P91-15B and ∆PISH × Kyu77-07A depict compatible (Susceptible) reaction. P91-15B and Kyu77-07A are *M. oryzae* strains while Pia, Pish and ∆PISH represent Nipponbare (NB) genotypes carrying respective genes (*Pia* and *Pish*).

Os02g0624300


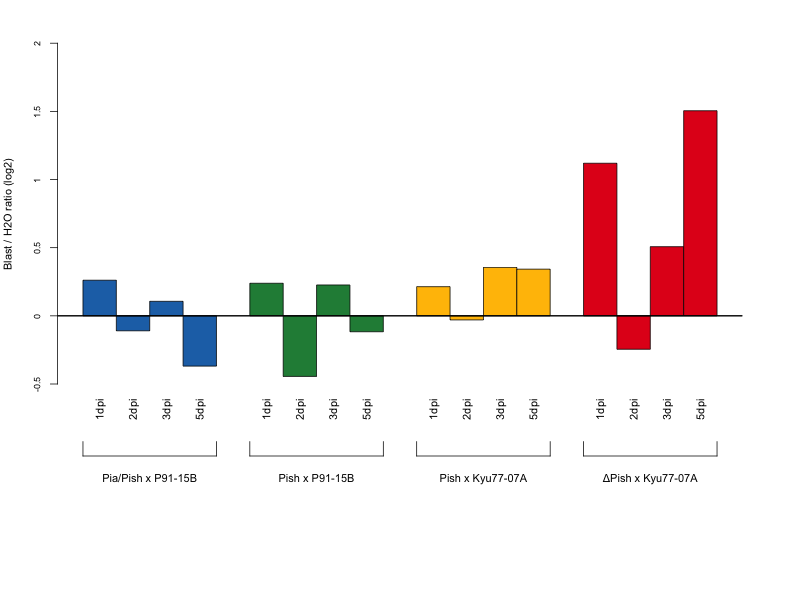
 **Fig. S6(XXXV)**: Graphical representation of fold-change in expression of Os02g0664000 gene at 1, 2, 3 and 5- days post-inoculation (dpi) with two *M. oryzae* strains from RiceXPro. Treatments, Pia/Pish × P91-15B and PISH × Kyu77-07A depict incompatible (Resistant) reaction and Pish × P91-15B and ∆PISH × Kyu77-07A depict compatible (Susceptible) reaction. P91-15B and Kyu77-07A are *M. oryzae* strains while Pia, Pish and ∆PISH represent Nipponbare (NB) genotypes carrying respective genes (*Pia* and *Pish*).

Os02g0664000


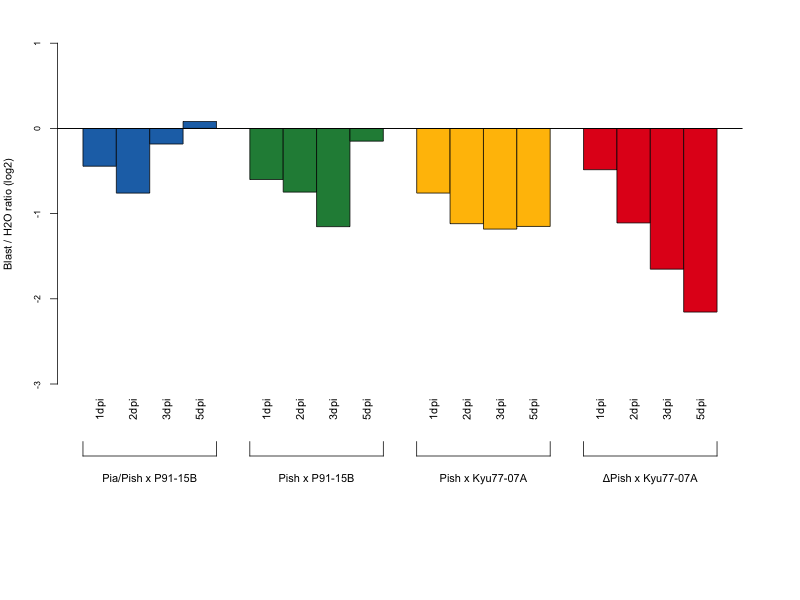
 **Fig. S6(XXXVI)**: Graphical representation of fold-change in expression of Os02g0665400 gene at 1, 2, 3 and 5- days post-inoculation (dpi) with two *M. oryzae* strains from RiceXPro. Treatments, Pia/Pish × P91-15B and PISH × Kyu77-07A depict incompatible (Resistant) reaction and Pish × P91-15B and ∆PISH × Kyu77-07A depict compatible (Susceptible) reaction. P91-15B and Kyu77-07A are *M. oryzae* strains while Pia, Pish and ∆PISH represent Nipponbare (NB) genotypes carrying respective genes (*Pia* and *Pish*).

Os02g0665400


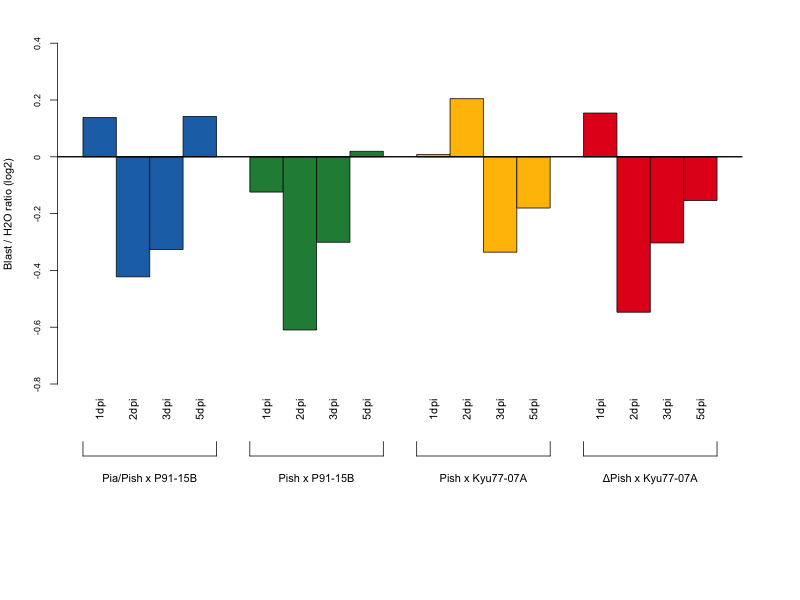
 **Fig. S6(XXXVII)**: Graphical representation of fold-change in expression of Os02g0743100 gene at 1, 2, 3 and 5- days post-inoculation (dpi) with two *M. oryzae* strains from RiceXPro. Treatments, Pia/Pish × P91-15B and PISH × Kyu77-07A depict incompatible (Resistant) reaction and Pish × P91-15B and ∆PISH × Kyu77-07A depict compatible (Susceptible) reaction. P91-15B and Kyu77-07A are *M. oryzae* strains while Pia, Pish and ∆PISH represent Nipponbare (NB) genotypes carrying respective genes (*Pia* and *Pish*).

Os02g0743100


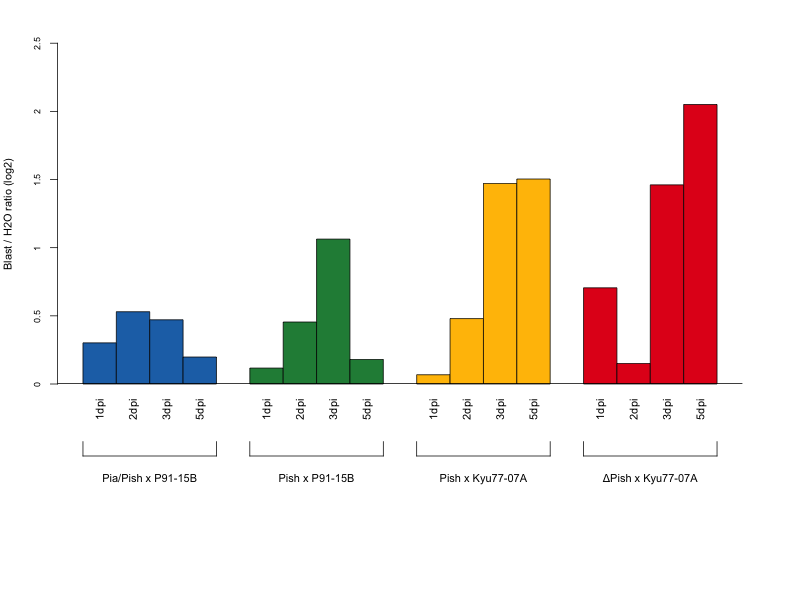
 **Fig. S6(XXXVIII)**: Graphical representation of fold-change in expression of Os02g0745600 gene at 1, 2, 3 and 5- days post-inoculation (dpi) with two *M. oryzae* strains from RiceXPro. Treatments, Pia/Pish × P91-15B and PISH × Kyu77-07A depict incompatible (Resistant) reaction and Pish × P91-15B and ∆PISH × Kyu77-07A depict compatible (Susceptible) reaction. P91-15B and Kyu77-07A are *M. oryzae* strains while Pia, Pish and ∆PISH represent Nipponbare (NB) genotypes carrying respective genes (*Pia* and *Pish*).

Os02g0745600


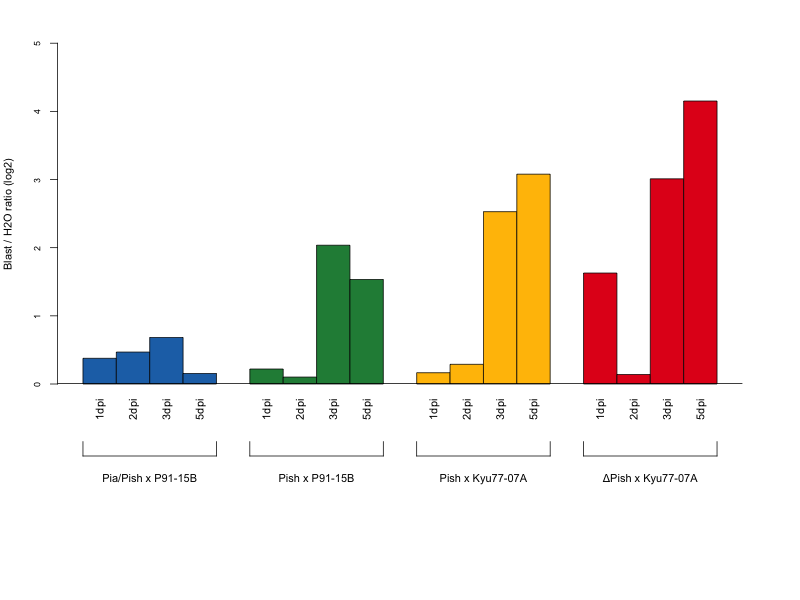
 **Fig. S6(XXXIX)**: Graphical representation of fold-change in expression of Os02g0759400 gene at 1, 2, 3 and 5- days post-inoculation (dpi) with two *M. oryzae* strains from RiceXPro. Treatments, Pia/Pish × P91-15B and PISH × Kyu77-07A depict incompatible (Resistant) reaction and Pish × P91-15B and ∆PISH × Kyu77-07A depict compatible (Susceptible) reaction. P91-15B and Kyu77-07A are *M. oryzae* strains while Pia, Pish and ∆PISH represent Nipponbare (NB) genotypes carrying respective genes (*Pia* and *Pish*).

Os02g0759400


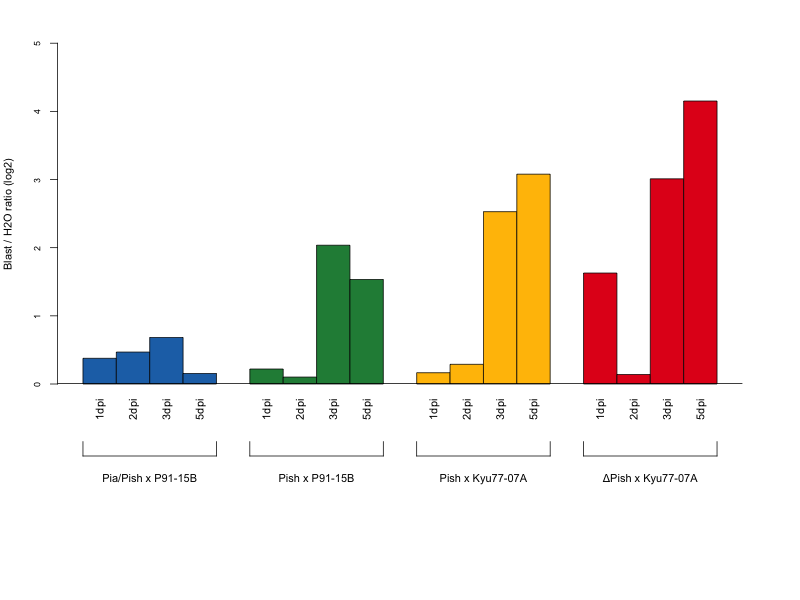
 **Fig. S6(XL)**: Graphical representation of fold-change in expression of Os02g0759400 gene at 1, 2, 3 and 5- days post-inoculation (dpi) with two *M. oryzae* strains from RiceXPro. Treatments, Pia/Pish × P91-15B and PISH × Kyu77-07A depict incompatible (Resistant) reaction and Pish × P91-15B and ∆PISH × Kyu77-07A depict compatible (Susceptible) reaction. P91-15B and Kyu77-07A are *M. oryzae* strains while Pia, Pish and ∆PISH represent Nipponbare (NB) genotypes carrying respective genes (*Pia* and *Pish*).

Os02g0759400


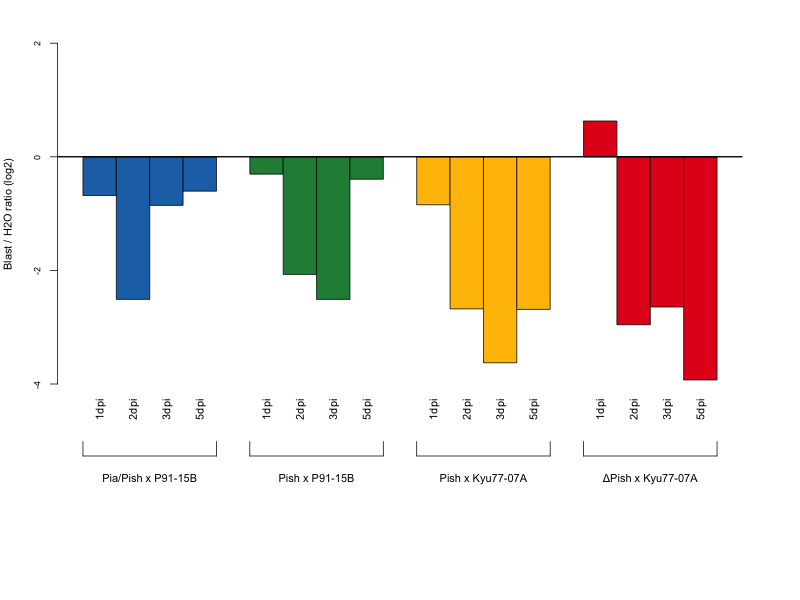
 **Fig. S6(XLI)**: Graphical representation of fold-change in expression of Os02g0764500 gene at 1, 2, 3 and 5- days post-inoculation (dpi) with two *M. oryzae* strains from RiceXPro. Treatments, Pia/Pish × P91-15B and PISH × Kyu77-07A depict incompatible (Resistant) reaction and Pish × P91-15B and ∆PISH × Kyu77-07A depict compatible (Susceptible) reaction. P91-15B and Kyu77-07A are *M. oryzae* strains while Pia, Pish and ∆PISH represent Nipponbare (NB) genotypes carrying respective genes (*Pia* and *Pish*).

Os02g0764500


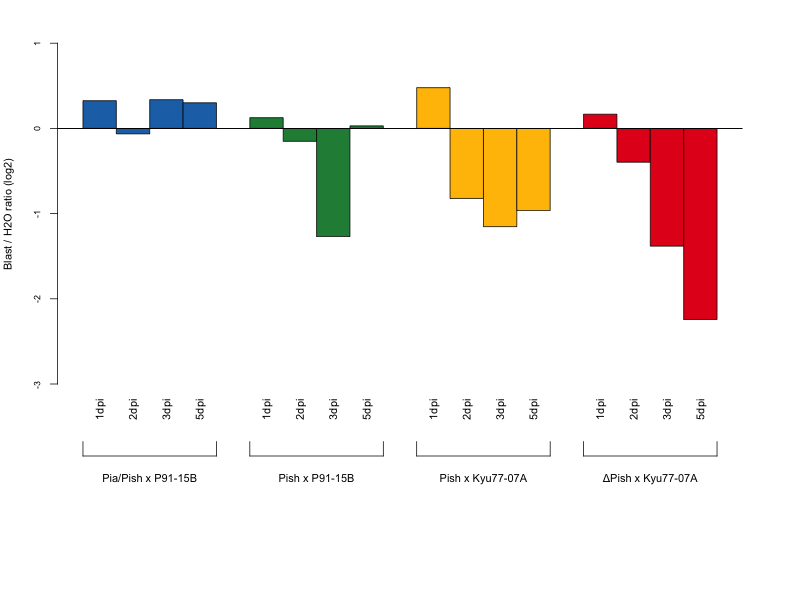
 **Fig. S6(LXII)**: Graphical representation of fold-change in expression of Os03g0328000 gene at 1, 2, 3 and 5- days post-inoculation (dpi) with two *M. oryzae* strains from RiceXPro. Treatments, Pia/Pish × P91-15B and PISH × Kyu77-07A depict incompatible (Resistant) reaction and Pish × P91-15B and ∆PISH × Kyu77-07A depict compatible (Susceptible) reaction. P91-15B and Kyu77-07A are *M. oryzae* strains while Pia, Pish and ∆PISH represent Nipponbare (NB) genotypes carrying respective genes (*Pia* and *Pish*).

Os03g0328000


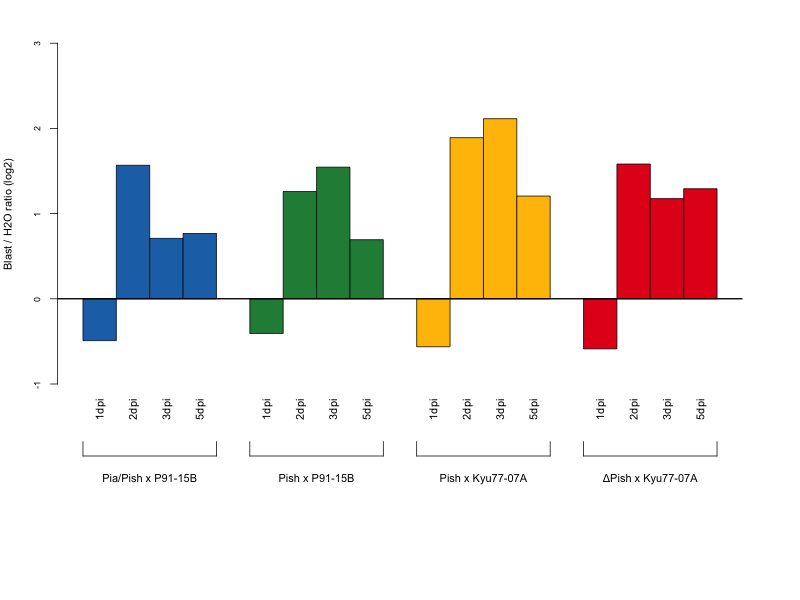
 **Fig. S6(LXIII)**: Graphical representation of fold-change in expression of Os03g0364400 gene at 1, 2, 3 and 5- days post-inoculation (dpi) with two *M. oryzae* strains from RiceXPro. Treatments, Pia/Pish × P91-15B and PISH × Kyu77-07A depict incompatible (Resistant) reaction and Pish × P91-15B and ∆PISH × Kyu77-07A depict compatible (Susceptible) reaction. P91-15B and Kyu77-07A are *M. oryzae* strains while Pia, Pish and ∆PISH represent Nipponbare (NB) genotypes carrying respective genes (*Pia* and *Pish*).

Os03g0364400


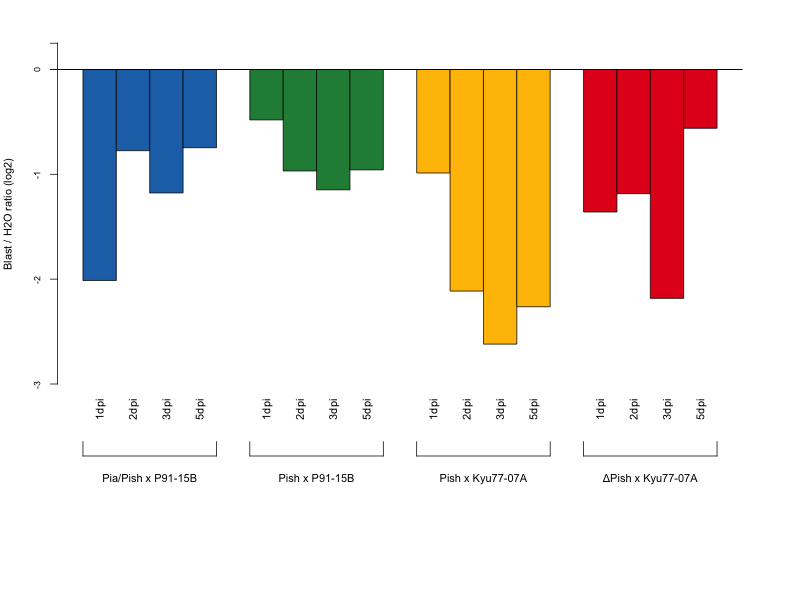
 **Fig. S6(LXIV)**: Graphical representation of fold-change in expression of Os03g0674700 gene at 1, 2, 3 and 5- days post-inoculation (dpi) with two *M. oryzae* strains from RiceXPro. Treatments, Pia/Pish × P91-15B and PISH × Kyu77-07A depict incompatible (Resistant) reaction and Pish × P91-15B and ∆PISH × Kyu77-07A depict compatible (Susceptible) reaction. P91-15B and Kyu77-07A are *M. oryzae* strains while Pia, Pish and ∆PISH represent Nipponbare (NB) genotypes carrying respective genes (*Pia* and *Pish*).

Os03g0674700


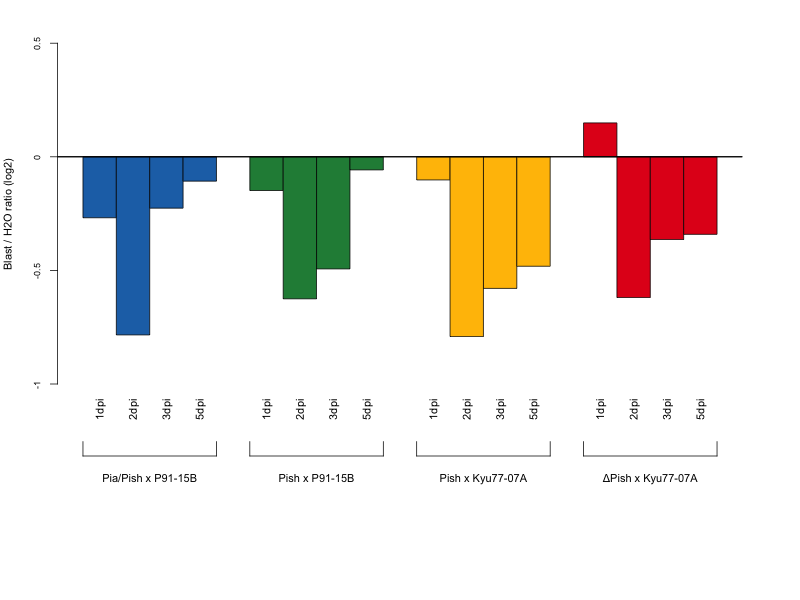
 **Fig. S6(LXV)**: Graphical representation of fold-change in expression of Os03g0680800 gene at 1, 2, 3 and 5- days post-inoculation (dpi) with two *M. oryzae* strains from RiceXPro. Treatments, Pia/Pish × P91-15B and PISH × Kyu77-07A depict incompatible (Resistant) reaction and Pish × P91-15B and ∆PISH × Kyu77-07A depict compatible (Susceptible) reaction. P91-15B and Kyu77-07A are *M. oryzae* strains while Pia, Pish and ∆PISH represent Nipponbare (NB) genotypes carrying respective genes (*Pia* and *Pish*).

Os03g0680800


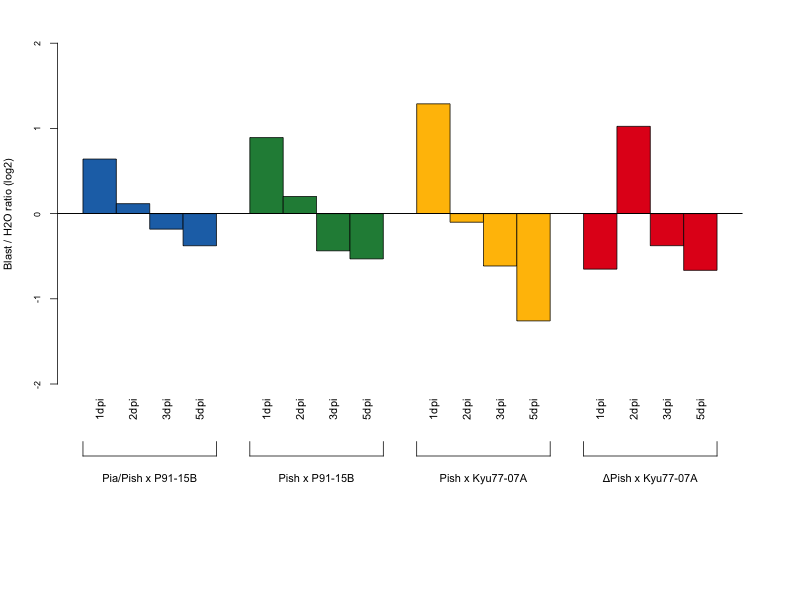
 **Fig. S6(LXVI)**: Graphical representation of fold-change in expression of Os03g0794500 gene at 1, 2, 3 and 5- days post-inoculation (dpi) with two *M. oryzae* strains from RiceXPro. Treatments, Pia/Pish × P91-15B and PISH × Kyu77-07A depict incompatible (Resistant) reaction and Pish × P91-15B and ∆PISH × Kyu77-07A depict compatible (Susceptible) reaction. P91-15B and Kyu77-07A are *M. oryzae* strains while Pia, Pish and ∆PISH represent Nipponbare (NB) genotypes carrying respective genes (*Pia* and *Pish*).

Os03g0794500


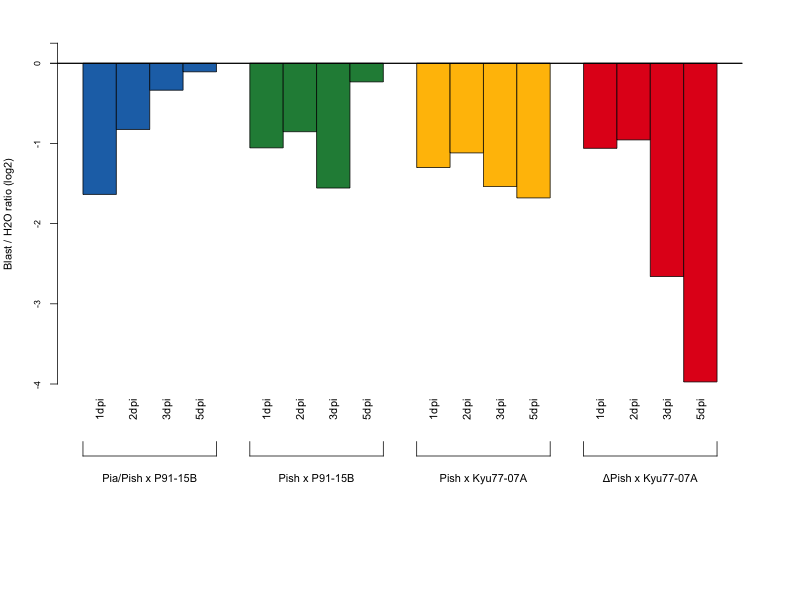
 **Fig. S6(LXVII)**: Graphical representation of fold-change in expression of Os04g0169500 gene at 1, 2, 3 and 5- days post-inoculation (dpi) with two *M. oryzae* strains from RiceXPro. Treatments, Pia/Pish × P91-15B and PISH × Kyu77-07A depict incompatible (Resistant) reaction and Pish × P91-15B and ∆PISH × Kyu77-07A depict compatible (Susceptible) reaction. P91-15B and Kyu77-07A are *M. oryzae* strains while Pia, Pish and ∆PISH represent Nipponbare (NB) genotypes carrying respective genes (*Pia* and *Pish*).

Os04g0169500


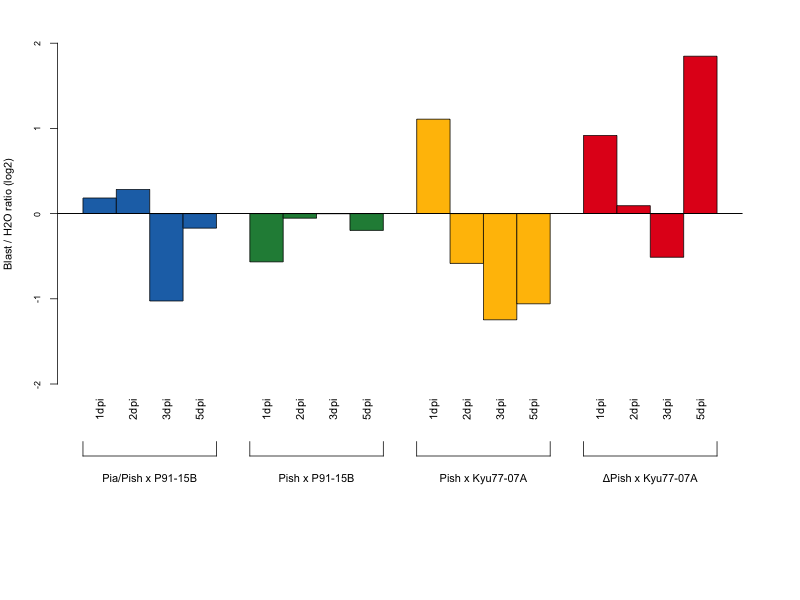
 **Fig. S6(LXVIII)**: Graphical representation of fold-change in expression of Os04g0177300 gene at 1, 2, 3 and 5- days post-inoculation (dpi) with two *M. oryzae* strains from RiceXPro. Treatments, Pia/Pish × P91-15B and PISH × Kyu77-07A depict incompatible (Resistant) reaction and Pish × P91-15B and ∆PISH × Kyu77-07A depict compatible (Susceptible) reaction. P91-15B and Kyu77-07A are *M. oryzae* strains while Pia, Pish and ∆PISH represent Nipponbare (NB) genotypes carrying respective genes (*Pia* and *Pish*).

Os04g0177300


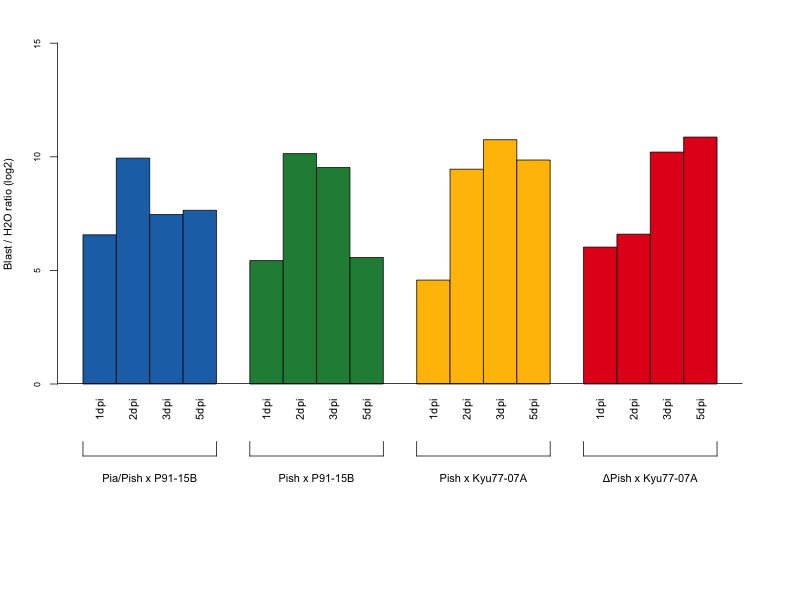
 **Fig. S6(LXIX)**: Graphical representation of fold-change in expression of Os04g0178300 gene at 1, 2, 3 and 5- days post-inoculation (dpi) with two *M. oryzae* strains from RiceXPro. Treatments, Pia/Pish × P91-15B and PISH × Kyu77-07A depict incompatible (Resistant) reaction and Pish × P91-15B and ∆PISH × Kyu77-07A depict compatible (Susceptible) reaction. P91-15B and Kyu77-07A are *M. oryzae* strains while Pia, Pish and ∆PISH represent Nipponbare (NB) genotypes carrying respective genes (*Pia* and *Pish*).

Os04g0178300


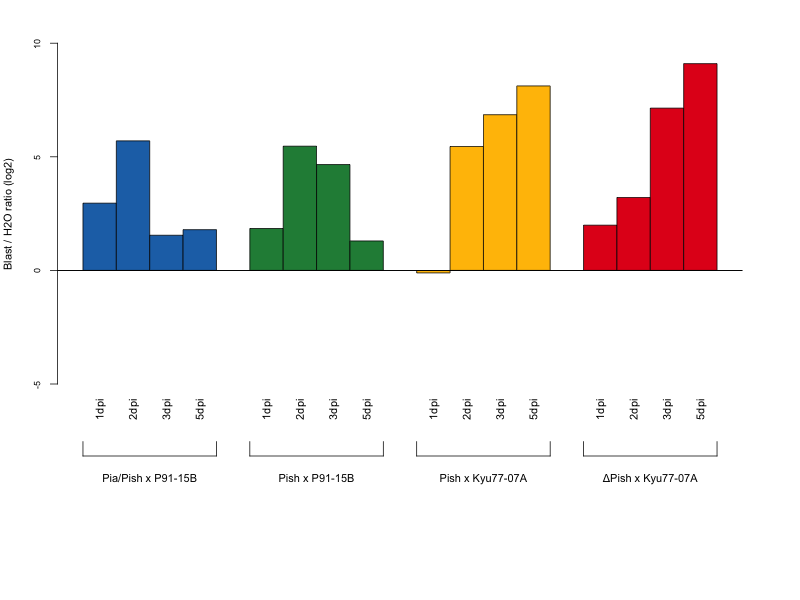
 **Fig. S6(L)**: Graphical representation of fold-change in expression of Os04g0179200 gene at 1, 2, 3 and 5- days post-inoculation (dpi) with two *M. oryzae* strains from RiceXPro. Treatments, Pia/Pish × P91-15B and PISH × Kyu77-07A depict incompatible (Resistant) reaction and Pish × P91-15B and ∆PISH × Kyu77-07A depict compatible (Susceptible) reaction. P91-15B and Kyu77-07A are *M. oryzae* strains while Pia, Pish and ∆PISH represent Nipponbare (NB) genotypes carrying respective genes (*Pia* and *Pish*).

Os04g0179200


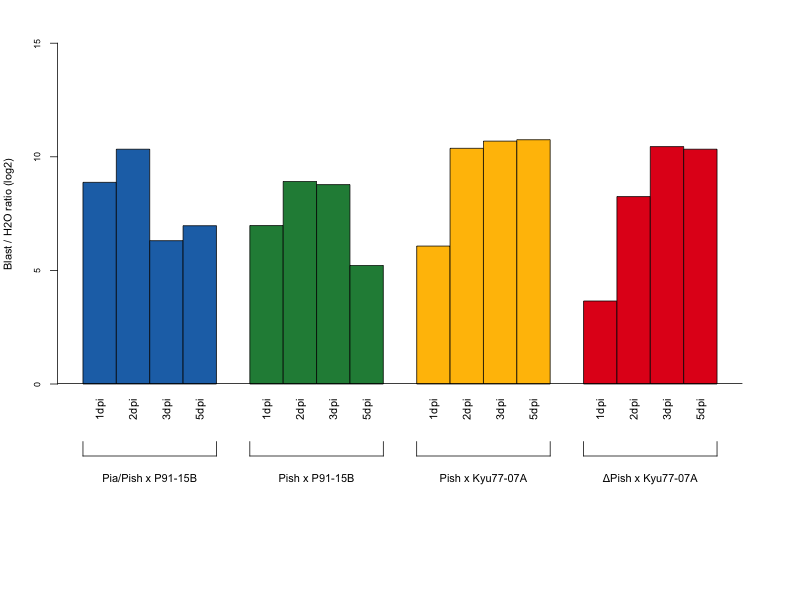
 **Fig. S6(LI)**: Graphical representation of fold-change in expression of Os04g0179700 gene at 1, 2, 3 and 5- days post-inoculation (dpi) with two *M. oryzae* strains from RiceXPro. Treatments, Pia/Pish × P91-15B and PISH × Kyu77-07A depict incompatible (Resistant) reaction and Pish × P91-15B and ∆PISH × Kyu77-07A depict compatible (Susceptible) reaction. P91-15B and Kyu77-07A are *M. oryzae* strains while Pia, Pish and ∆PISH represent Nipponbare (NB) genotypes carrying respective genes (*Pia* and *Pish*).

Os04g0179700


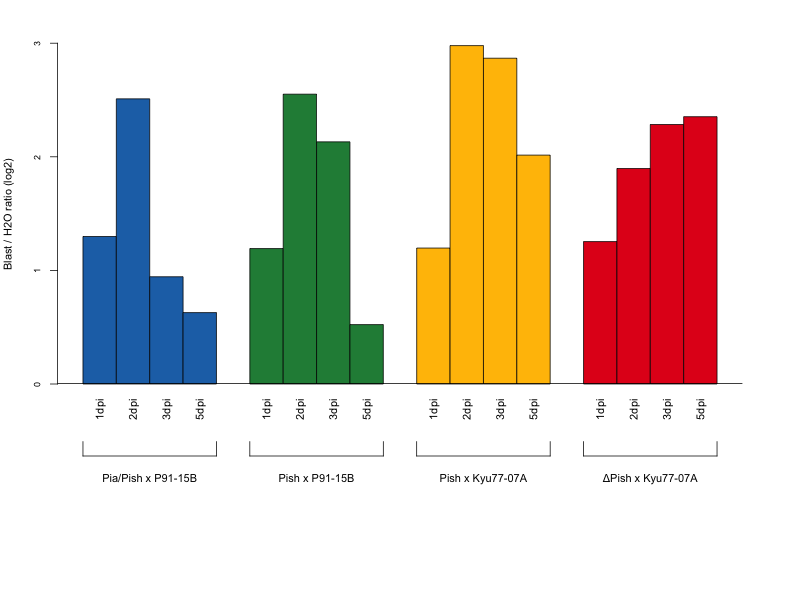
 **Fig. S6(LII)**: Graphical representation of fold-change in expression of Os04g0206700 gene at 1, 2, 3 and 5- days post-inoculation (dpi) with two *M. oryzae* strains from RiceXPro. Treatments, Pia/Pish × P91-15B and PISH × Kyu77-07A depict incompatible (Resistant) reaction and Pish × P91-15B and ∆PISH × Kyu77-07A depict compatible (Susceptible) reaction. P91-15B and Kyu77-07A are *M. oryzae* strains while Pia, Pish and ∆PISH represent Nipponbare (NB) genotypes carrying respective genes (*Pia* and *Pish*).

Os04g0206700


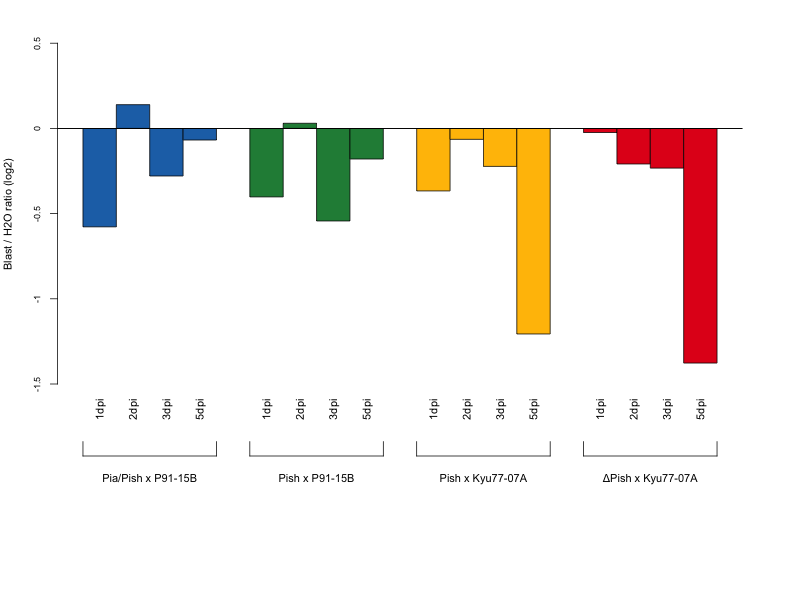
 **Fig. S6(LIII)**: Graphical representation of fold-change in expression of Os04g0509300 gene at 1, 2, 3 and 5- days post-inoculation (dpi) with two *M. oryzae* strains from RiceXPro. Treatments, Pia/Pish × P91-15B and PISH × Kyu77-07A depict incompatible (Resistant) reaction and Pish × P91-15B and ∆PISH × Kyu77-07A depict compatible (Susceptible) reaction. P91-15B and Kyu77-07A are *M. oryzae* strains while Pia, Pish and ∆PISH represent Nipponbare (NB) genotypes carrying respective genes (*Pia* and *Pish*).

Os04g0509300


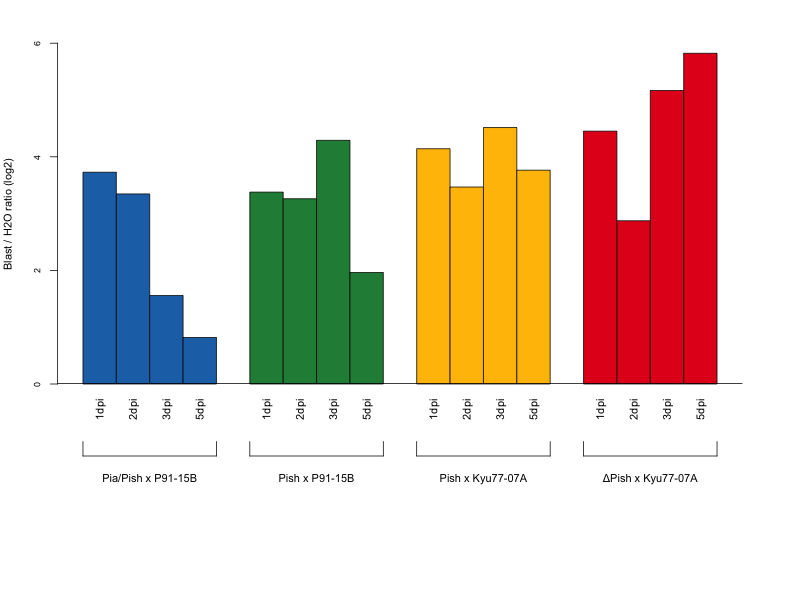
 **Fig. S6(LIV)**: Graphical representation of fold-change in expression of Os04g0517100 gene at 1, 2, 3 and 5- days post-inoculation (dpi) with two *M. oryzae* strains from RiceXPro. Treatments, Pia/Pish × P91-15B and PISH × Kyu77-07A depict incompatible (Resistant) reaction and Pish × P91-15B and ∆PISH × Kyu77-07A depict compatible (Susceptible) reaction. P91-15B and Kyu77-07A are *M. oryzae* strains while Pia, Pish and ∆PISH represent Nipponbare (NB) genotypes carrying respective genes (*Pia* and *Pish*).

Os04g0517100


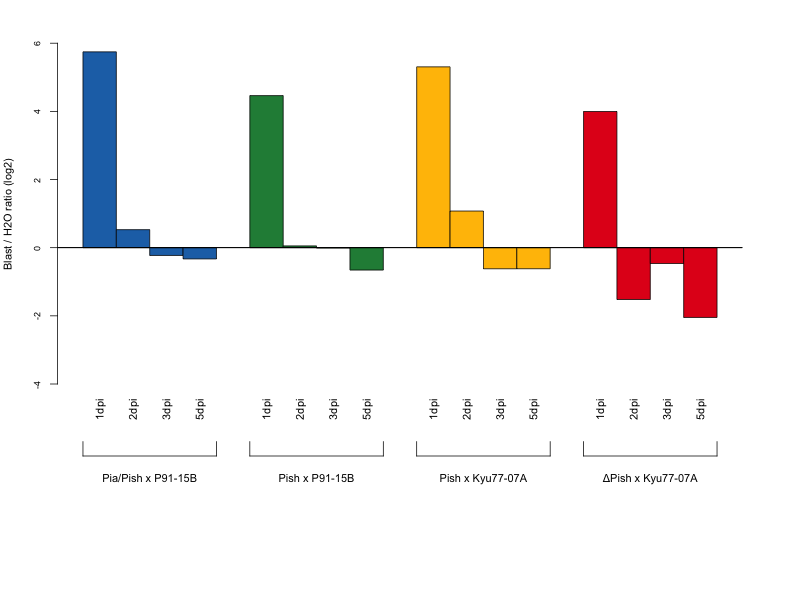
 **Fig. S6(LV)**: Graphical representation of fold-change in expression of Os04g0518400 gene at 1, 2, 3 and 5- days post-inoculation (dpi) with two *M. oryzae* strains from RiceXPro. Treatments, Pia/Pish × P91-15B and PISH × Kyu77-07A depict incompatible (Resistant) reaction and Pish × P91-15B and ∆PISH × Kyu77-07A depict compatible (Susceptible) reaction. P91-15B and Kyu77-07A are *M. oryzae* strains while Pia, Pish and ∆PISH represent Nipponbare (NB) genotypes carrying respective genes (*Pia* and *Pish*).

Os04g0518400


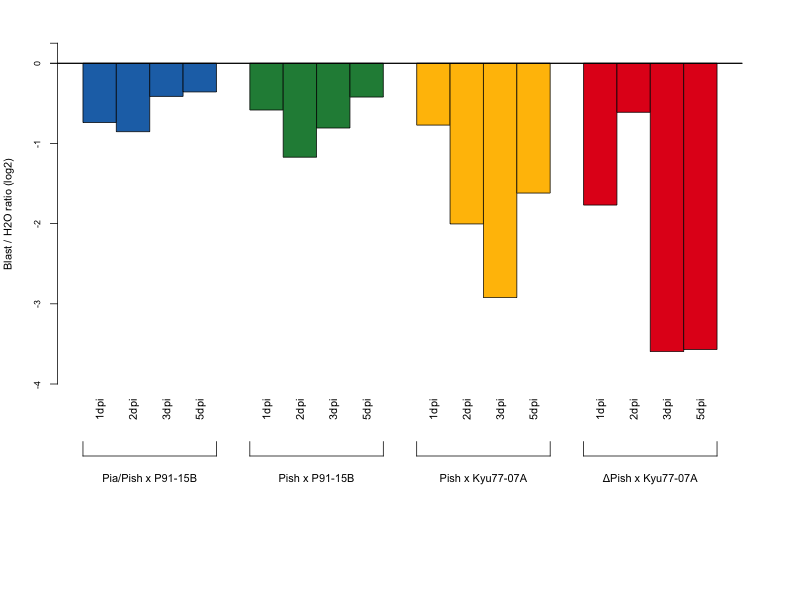
 **Fig. S6(LVI)**: Graphical representation of fold-change in expression of Os04g0520700 gene at 1, 2, 3 and 5- days post-inoculation (dpi) with two *M. oryzae* strains from RiceXPro. Treatments, Pia/Pish × P91-15B and PISH × Kyu77-07A depict incompatible (Resistant) reaction and Pish × P91-15B and ∆PISH × Kyu77-07A depict compatible (Susceptible) reaction. P91-15B and Kyu77-07A are *M. oryzae* strains while Pia, Pish and ∆PISH represent Nipponbare (NB) genotypes carrying respective genes (*Pia* and *Pish*).

Os04g0520700


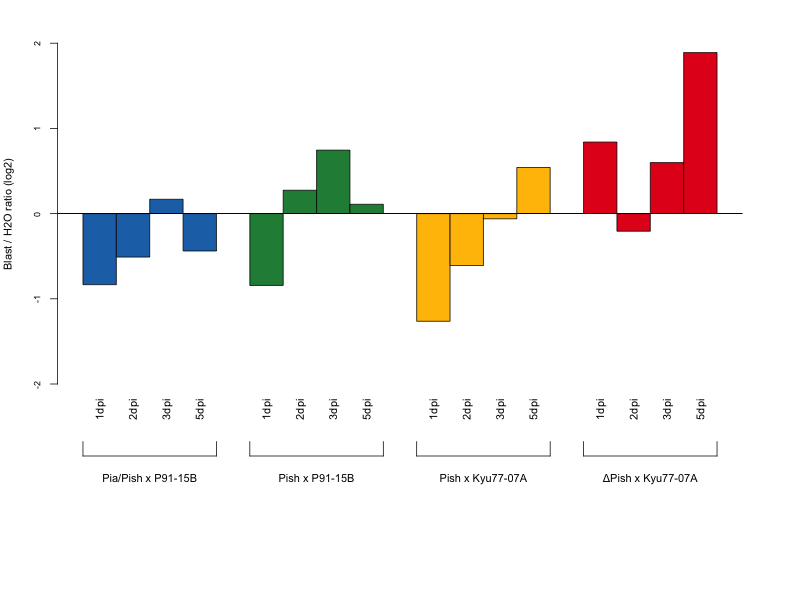
 **Fig. S6(LVII)**: Graphical representation of fold-change in expression of Os04g0573200 gene at 1, 2, 3 and 5- days post-inoculation (dpi) with two *M. oryzae* strains from RiceXPro. Treatments, Pia/Pish × P91-15B and PISH × Kyu77-07A depict incompatible (Resistant) reaction and Pish × P91-15B and ∆PISH × Kyu77-07A depict compatible (Susceptible) reaction. P91-15B and Kyu77-07A are *M. oryzae* strains while Pia, Pish and ∆PISH represent Nipponbare (NB) genotypes carrying respective genes (*Pia* and *Pish*).

Os04g0573200


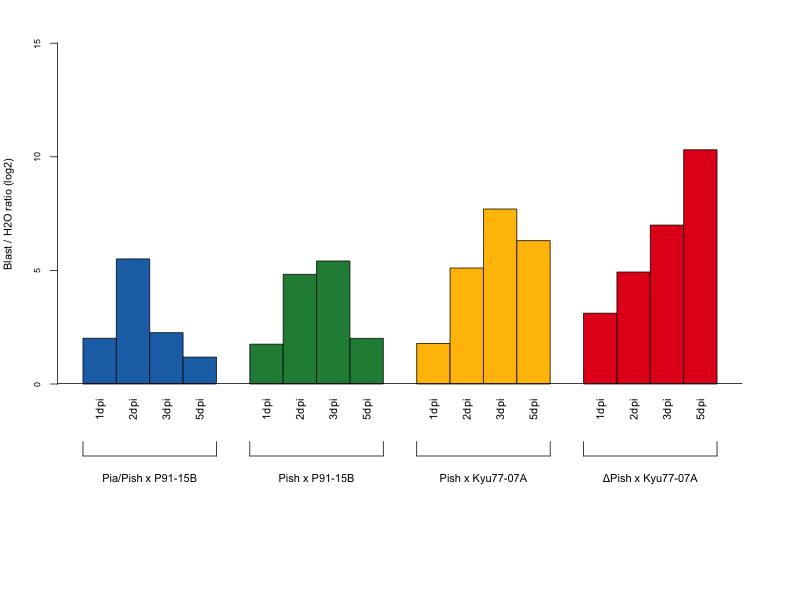
 **Fig. S6(LVIII)**: Graphical representation of fold-change in expression of Os04g0578000 gene at 1, 2, 3 and 5- days post-inoculation (dpi) with two *M. oryzae* strains from RiceXPro. Treatments, Pia/Pish × P91-15B and PISH × Kyu77-07A depict incompatible (Resistant) reaction and Pish × P91-15B and ∆PISH × Kyu77-07A depict compatible (Susceptible) reaction. P91-15B and Kyu77-07A are *M. oryzae* strains while Pia, Pish and ∆PISH represent Nipponbare (NB) genotypes carrying respective genes (*Pia* and *Pish*).

Os04g0578000


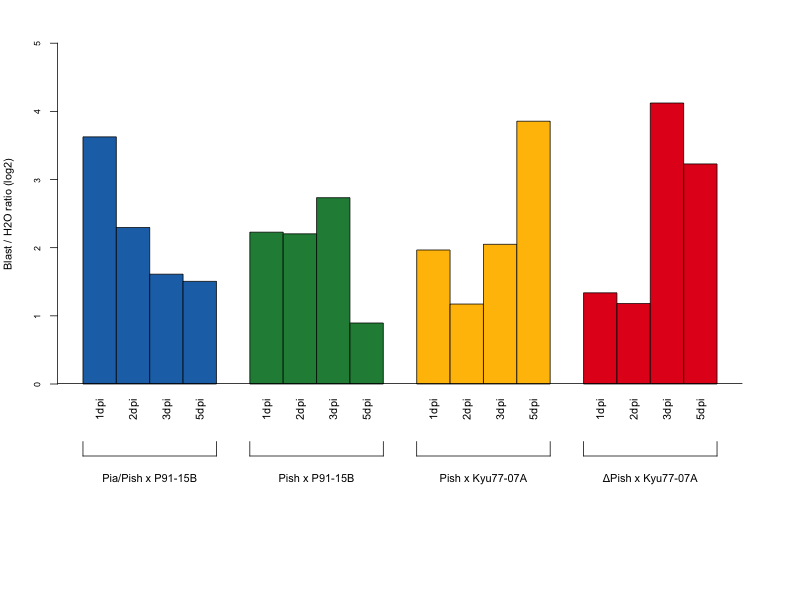
 **Fig. S6(LIX)**: Graphical representation of fold-change in expression of Os04g0664900 gene at 1, 2, 3 and 5- days post-inoculation (dpi) with two *M. oryzae* strains from RiceXPro. Treatments, Pia/Pish × P91-15B and PISH × Kyu77-07A depict incompatible (Resistant) reaction and Pish × P91-15B and ∆PISH × Kyu77-07A depict compatible (Susceptible) reaction. P91-15B and Kyu77-07A are *M. oryzae* strains while Pia, Pish and ∆PISH represent Nipponbare (NB) genotypes carrying respective genes (*Pia* and *Pish*).

Os04g0664900


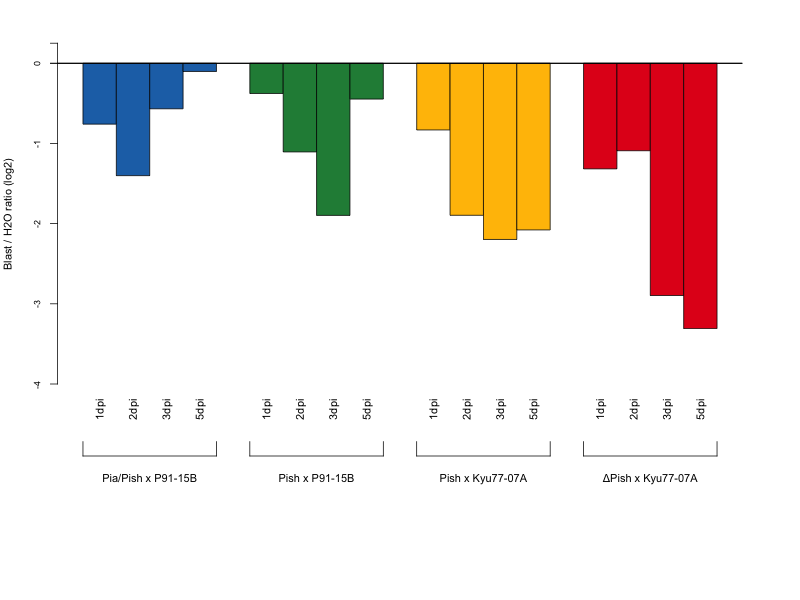
 **Fig. S6(LX)**: Graphical representation of fold-change in expression of Os04g0671900 gene at 1, 2, 3 and 5- days post-inoculation (dpi) with two *M. oryzae* strains from RiceXPro. Treatments, Pia/Pish × P91-15B and PISH × Kyu77-07A depict incompatible (Resistant) reaction and Pish × P91-15B and ∆PISH × Kyu77-07A depict compatible (Susceptible) reaction. P91-15B and Kyu77-07A are *M. oryzae* strains while Pia, Pish and ∆PISH represent Nipponbare (NB) genotypes carrying respective genes (*Pia* and *Pish*).

Os04g0671900


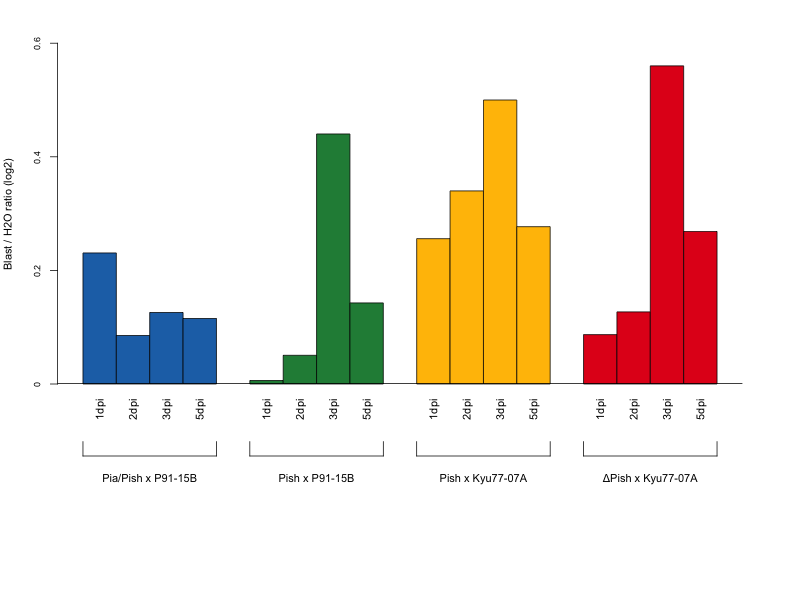
 **Fig. S6(LXI)**: Graphical representation of fold-change in expression of Os05g0135800gene at 1, 2, 3 and 5- days post-inoculation (dpi) with two *M. oryzae* strains from RiceXPro. Treatments, Pia/Pish × P91-15B and PISH × Kyu77-07A depict incompatible (Resistant) reaction and Pish × P91-15B and ∆PISH × Kyu77-07A depict compatible (Susceptible) reaction. P91-15B and Kyu77-07A are *M. oryzae* strains while Pia, Pish and ∆PISH represent Nipponbare (NB) genotypes carrying respective genes (*Pia* and *Pish*).

Os05g0135800


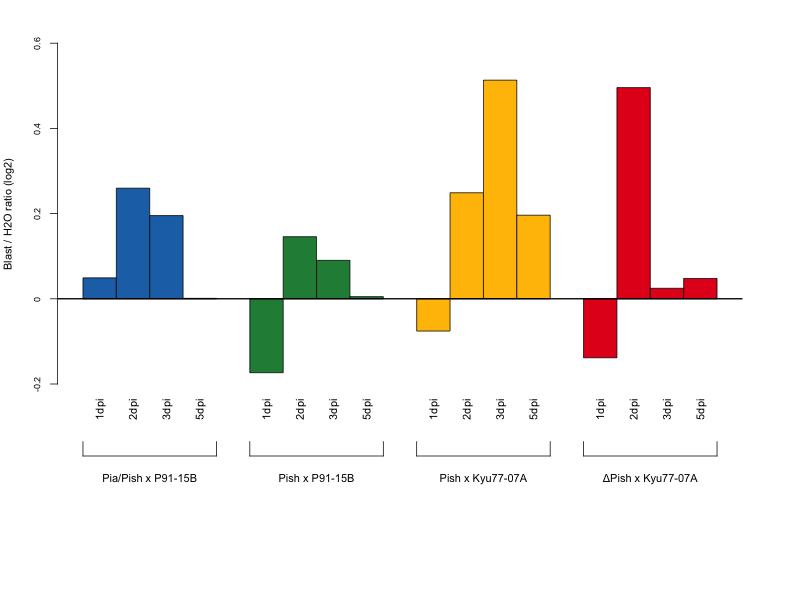
 **Fig. S6(LXII)**: Graphical representation of fold-change in expression of Os05g0150300 gene at 1, 2, 3 and 5- days post-inoculation (dpi) with two *M. oryzae* strains from RiceXPro. Treatments, Pia/Pish × P91-15B and PISH × Kyu77-07A depict incompatible (Resistant) reaction and Pish × P91-15B and ∆PISH × Kyu77-07A depict compatible (Susceptible) reaction. P91-15B and Kyu77-07A are *M. oryzae* strains while Pia, Pish and ∆PISH represent Nipponbare (NB) genotypes carrying respective genes (*Pia* and *Pish*).

Os05g0150300


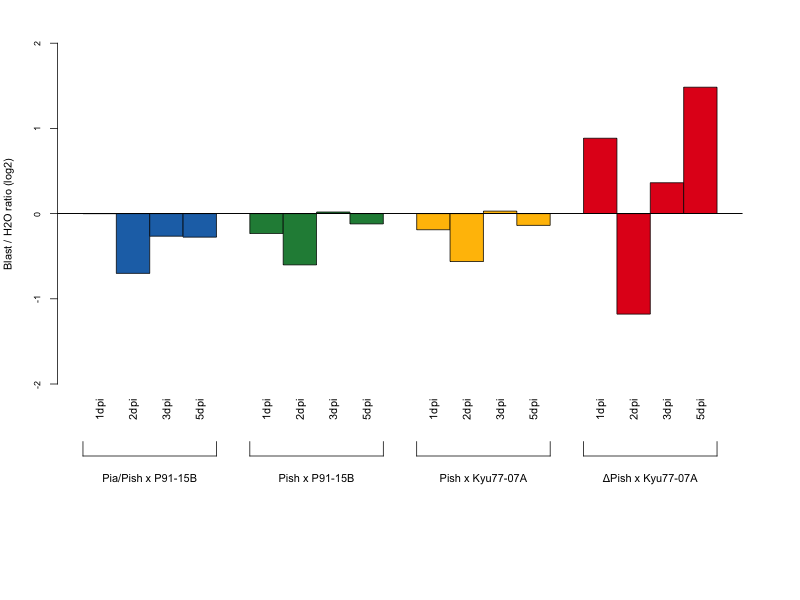
 **Fig. S6(v)**: Graphical representation of fold-change in expression of Os01g0343200 gene at 1, 2, 3 and 5- days post-inoculation (dpi) with two *M. oryzae* strains from RiceXPro. Treatments, Pia/Pish × P91-15B and PISH × Kyu77-07A depict incompatible (Resistant) reaction and Pish × P91-15B and ∆PISH × Kyu77-07A depict compatible (Susceptible) reaction. P91-15B and Kyu77-07A are *M. oryzae* strains while Pia, Pish and ∆PISH represent Nipponbare (NB) genotypes carrying respective genes (*Pia* and *Pish*).

Os05g0154600


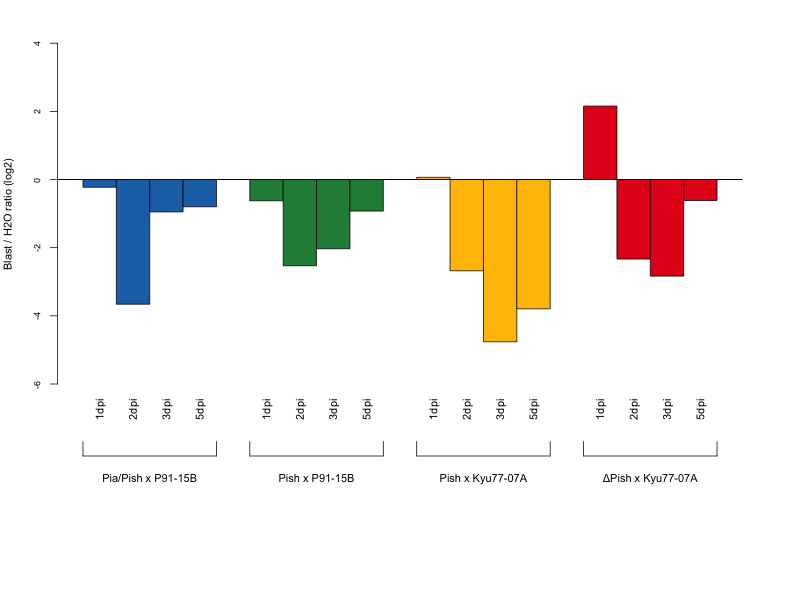
 **Fig. S6(LXIV)**: Graphical representation of fold-change in expression of Os05g0160300gene at 1, 2, 3 and 5- days post-inoculation (dpi) with two *M. oryzae* strains from RiceXPro. Treatments, Pia/Pish × P91-15B and PISH × Kyu77-07A depict incompatible (Resistant) reaction and Pish × P91-15B and ∆PISH × Kyu77-07A depict compatible (Susceptible) reaction. P91-15B and Kyu77-07A are *M. oryzae* strains while Pia, Pish and ∆PISH represent Nipponbare (NB) genotypes carrying respective genes (*Pia* and *Pish*).

Os05g0160300


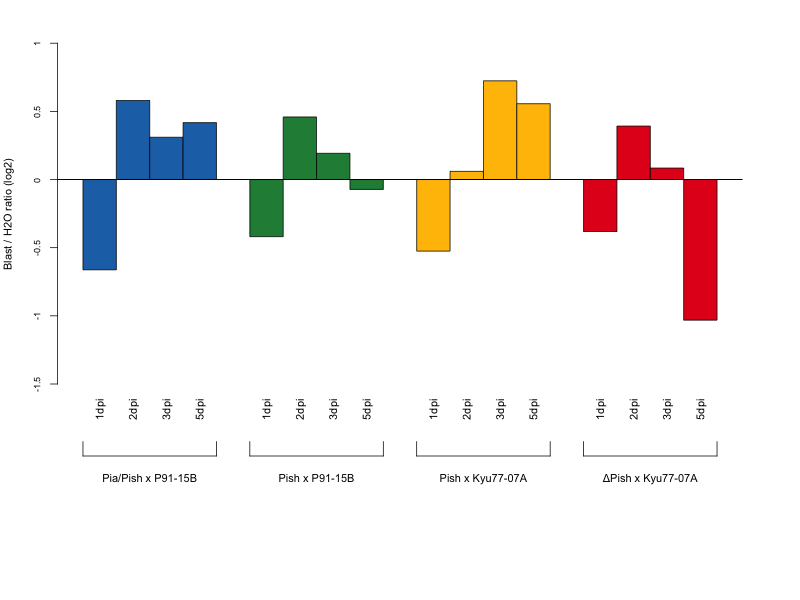
 **Fig. S6(LXV)**: Graphical representation of fold-change in expression of Os05g0164900 gene at 1, 2, 3 and 5- days post-inoculation (dpi) with two *M. oryzae* strains from RiceXPro. Treatments, Pia/Pish × P91-15B and PISH × Kyu77-07A depict incompatible (Resistant) reaction and Pish × P91-15B and ∆PISH × Kyu77-07A depict compatible (Susceptible) reaction. P91-15B and Kyu77-07A are *M. oryzae* strains while Pia, Pish and ∆PISH represent Nipponbare (NB) genotypes carrying respective genes (*Pia* and *Pish*).

Os05g0164900


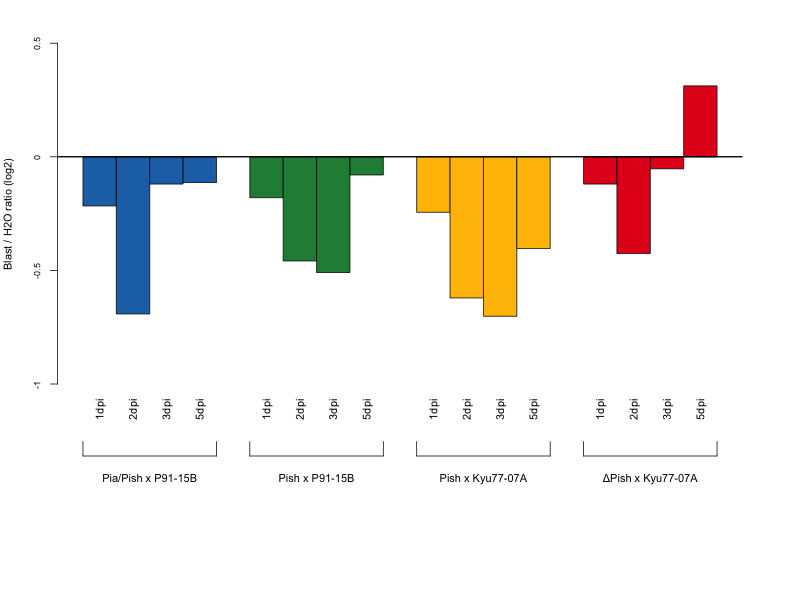
 **Fig. S6(LXVI)**: Graphical representation of fold-change in expression of Os05g0489600 gene at 1, 2, 3 and 5- days post-inoculation (dpi) with two *M. oryzae* strains from RiceXPro. Treatments, Pia/Pish × P91-15B and PISH × Kyu77-07A depict incompatible (Resistant) reaction and Pish × P91-15B and ∆PISH × Kyu77-07A depict compatible (Susceptible) reaction. P91-15B and Kyu77-07A are *M. oryzae* strains while Pia, Pish and ∆PISH represent Nipponbare (NB) genotypes carrying respective genes (*Pia* and *Pish*).

Os05g0489600


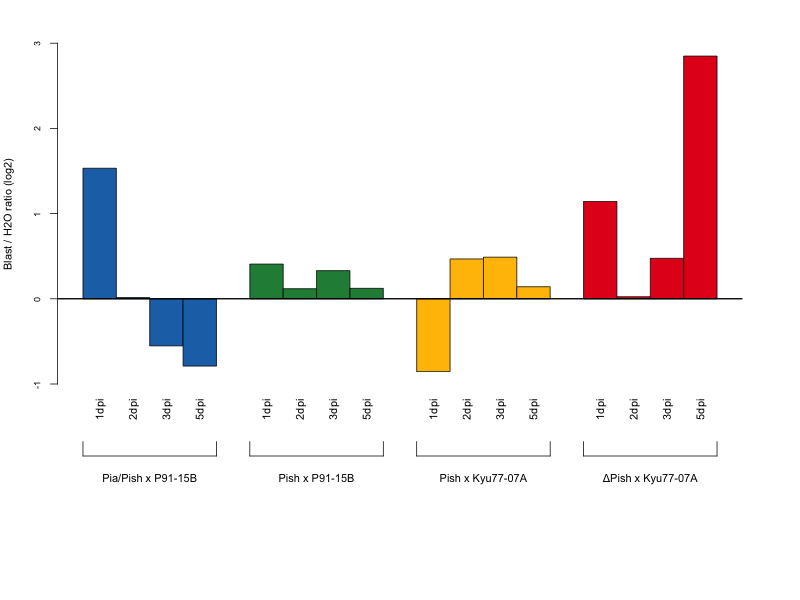
 **Fig. S6(LXVII)**: Graphical representation of fold-change in expression of Os05g0496400 gene at 1, 2, 3 and 5- days post-inoculation (dpi) with two *M. oryzae* strains from RiceXPro. Treatments, Pia/Pish × P91-15B and PISH × Kyu77-07A depict incompatible (Resistant) reaction and Pish × P91-15B and ∆PISH × Kyu77-07A depict compatible (Susceptible) reaction. P91-15B and Kyu77-07A are *M. oryzae* strains while Pia, Pish and ∆PISH represent Nipponbare (NB) genotypes carrying respective genes (*Pia* and *Pish*).

Os05g0496400


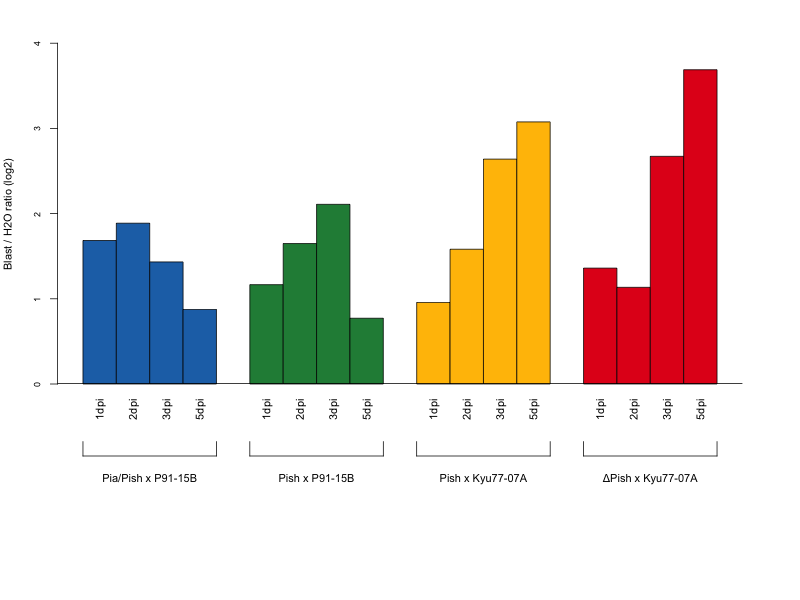
 **Fig. S6(LXVIII)**: Graphical representation of fold-change in expression of Os05g0496500 gene at 1, 2, 3 and 5- days post-inoculation (dpi) with two *M. oryzae* strains from RiceXPro. Treatments, Pia/Pish × P91-15B and PISH × Kyu77-07A depict incompatible (Resistant) reaction and Pish × P91-15B and ∆PISH × Kyu77-07A depict compatible (Susceptible) reaction. P91-15B and Kyu77-07A are *M. oryzae* strains while Pia, Pish and ∆PISH represent Nipponbare (NB) genotypes carrying respective genes (*Pia* and *Pish*).

Os05g0496500


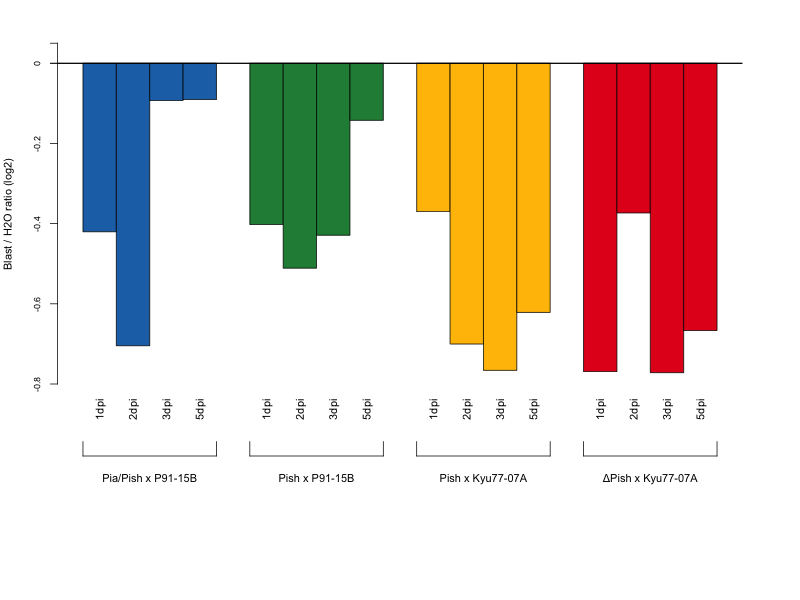
 **Fig. S6(LXIX)**: Graphical representation of fold-change in expression of Os05g0498700 gene at 1, 2, 3 and 5- days post-inoculation (dpi) with two *M. oryzae* strains from RiceXPro. Treatments, Pia/Pish × P91-15B and PISH × Kyu77-07A depict incompatible (Resistant) reaction and Pish × P91-15B and ∆PISH × Kyu77-07A depict compatible (Susceptible) reaction. P91-15B and Kyu77-07A are *M. oryzae* strains while Pia, Pish and ∆PISH represent Nipponbare (NB) genotypes carrying respective genes (*Pia* and *Pish*).

Os05g0498700


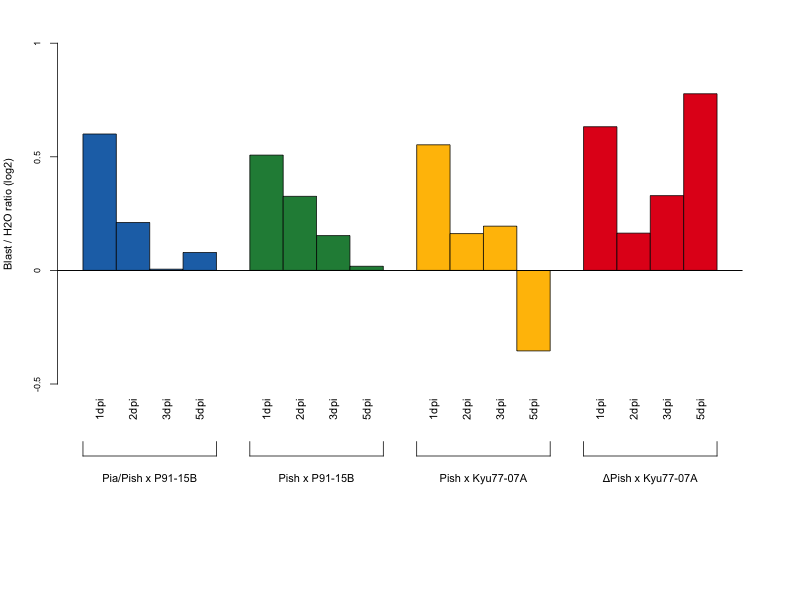
 **Fig. S6(LXX)**: Graphical representation of fold-change in expression of Os05g0522500 gene at 1, 2, 3 and 5- days post-inoculation (dpi) with two *M. oryzae* strains from RiceXPro. Treatments, Pia/Pish × P91-15B and PISH × Kyu77-07A depict incompatible (Resistant) reaction and Pish × P91-15B and ∆PISH × Kyu77-07A depict compatible (Susceptible) reaction. P91-15B and Kyu77-07A are *M. oryzae* strains while Pia, Pish and ∆PISH represent Nipponbare (NB) genotypes carrying respective genes (*Pia* and *Pish*).

Os05g0522500


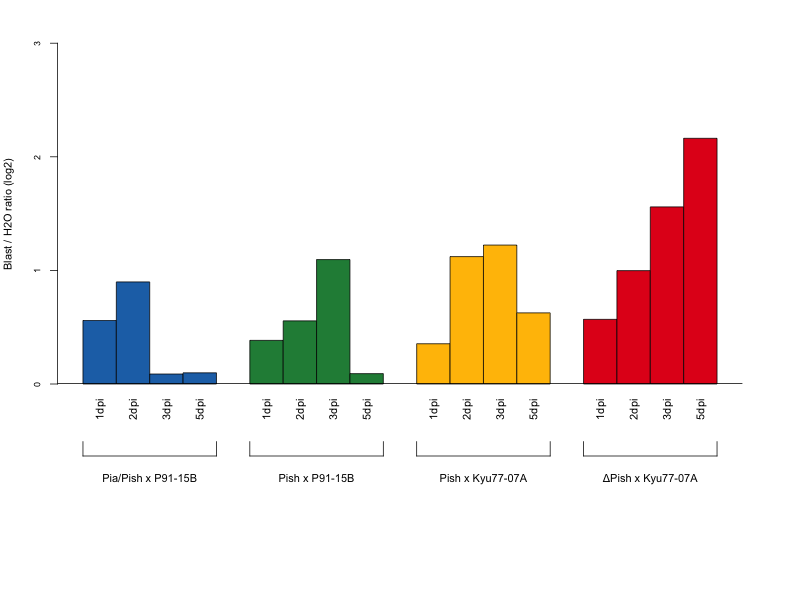
 **Fig. S6(LXXI)**: Graphical representation of fold-change in expression of Os06g0111400 gene at 1, 2, 3 and 5- days post-inoculation (dpi) with two *M. oryzae* strains from RiceXPro. Treatments, Pia/Pish × P91-15B and PISH × Kyu77-07A depict incompatible (Resistant) reaction and Pish × P91-15B and ∆PISH × Kyu77-07A depict compatible (Susceptible) reaction. P91-15B and Kyu77-07A are *M. oryzae* strains while Pia, Pish and ∆PISH represent Nipponbare (NB) genotypes carrying respective genes (*Pia* and *Pish*).

Os06g0111400


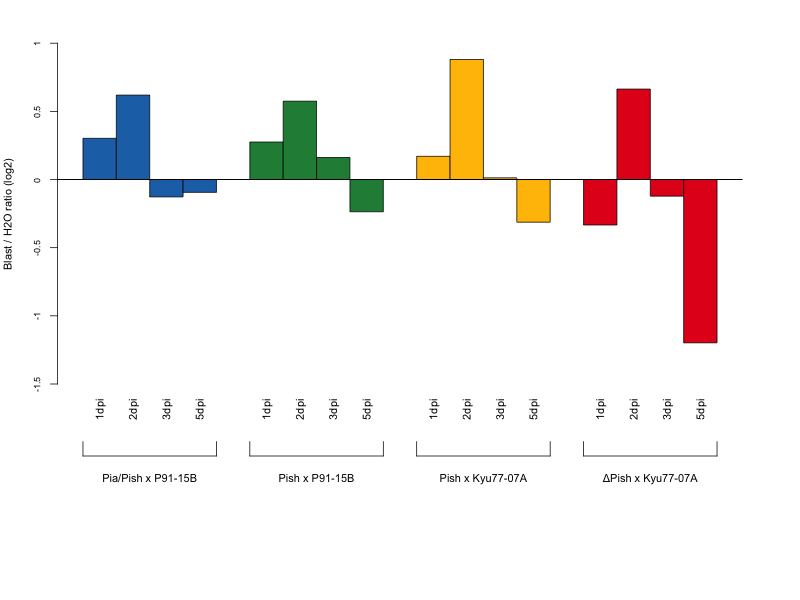
 **Fig. S6(LXXII)**: Graphical representation of fold-change in expression of Os06g0112400 gene at 1, 2, 3 and 5- days post-inoculation (dpi) with two *M. oryzae* strains from RiceXPro. Treatments, Pia/Pish × P91-15B and PISH × Kyu77-07A depict incompatible (Resistant) reaction and Pish × P91-15B and ∆PISH × Kyu77-07A depict compatible (Susceptible) reaction. P91-15B and Kyu77-07A are *M. oryzae* strains while Pia, Pish and ∆PISH represent Nipponbare (NB) genotypes carrying respective genes (*Pia* and *Pish*).

Os06g0112400


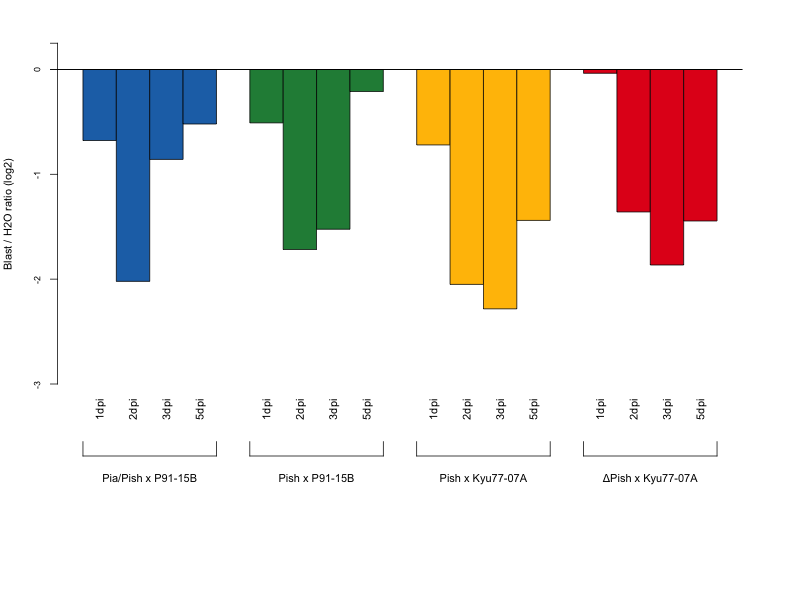
 **Fig. S6(LXXIII)**: Graphical representation of fold-change in expression of Os06g0115300 gene at 1, 2, 3 and 5- days post-inoculation (dpi) with two *M. oryzae* strains from RiceXPro. Treatments, Pia/Pish × P91-15B and PISH × Kyu77-07A depict incompatible (Resistant) reaction and Pish × P91-15B and ∆PISH × Kyu77-07A depict compatible (Susceptible) reaction. P91-15B and Kyu77-07A are *M. oryzae* strains while Pia, Pish and ∆PISH represent Nipponbare (NB) genotypes carrying respective genes (*Pia* and *Pish*).

Os06g0115300


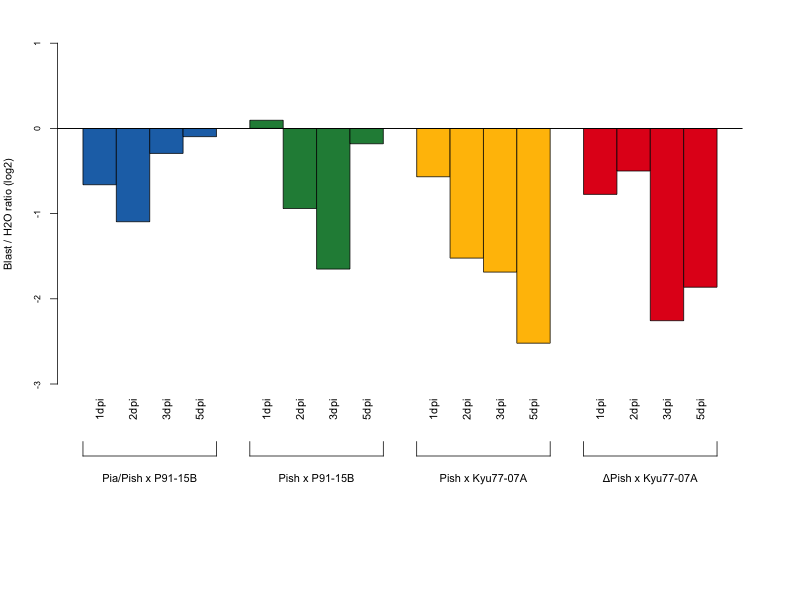
 **Fig. S6(LXXIV)**: Graphical representation of fold-change in expression of Os06g0116200 gene at 1, 2, 3 and 5- days post-inoculation (dpi) with two *M. oryzae* strains from RiceXPro. Treatments, Pia/Pish × P91-15B and PISH × Kyu77-07A depict incompatible (Resistant) reaction and Pish × P91-15B and ∆PISH × Kyu77-07A depict compatible (Susceptible) reaction. P91-15B and Kyu77-07A are *M. oryzae* strains while Pia, Pish and ∆PISH represent Nipponbare (NB) genotypes carrying respective genes (*Pia* and *Pish*).

Os06g0116200


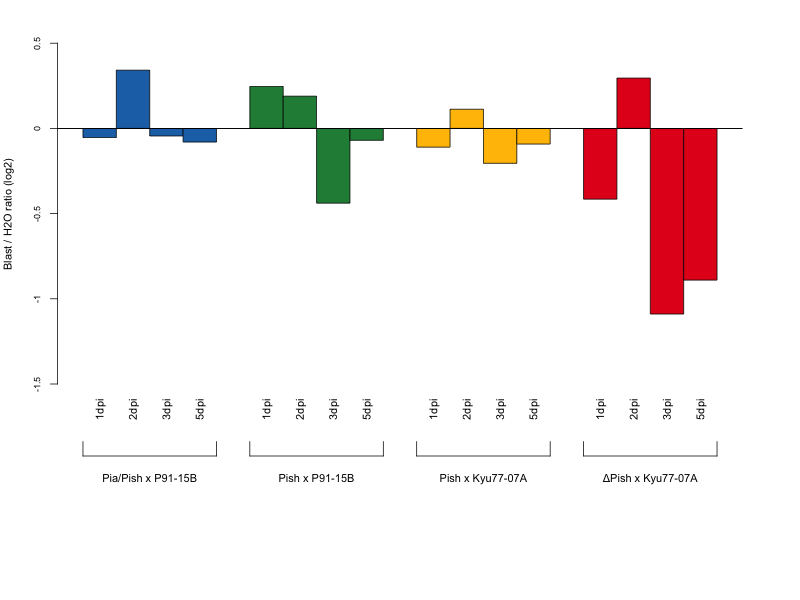
 **Fig. S6(LXXV)**: Graphical representation of fold-change in expression of Os06g0116800 gene at 1, 2, 3 and 5- days post-inoculation (dpi) with two *M. oryzae* strains from RiceXPro. Treatments, Pia/Pish × P91-15B and PISH × Kyu77-07A depict incompatible (Resistant) reaction and Pish × P91-15B and ∆PISH × Kyu77-07A depict compatible (Susceptible) reaction. P91-15B and Kyu77-07A are *M. oryzae* strains while Pia, Pish and ∆PISH represent Nipponbare (NB) genotypes carrying respective genes (*Pia* and *Pish*).

Os06g0116800


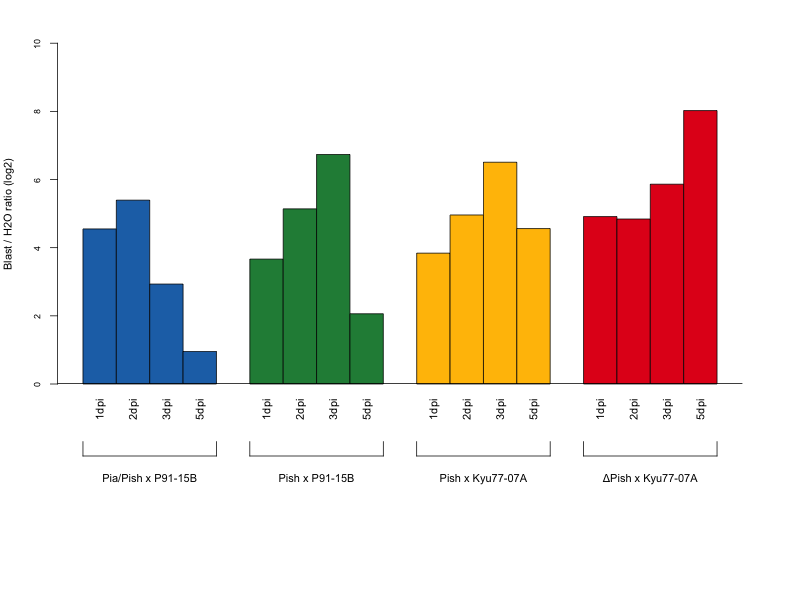
 **Fig. S6(LXXVI)**: Graphical representation of fold-change in expression of Os06g0128800 gene at 1, 2, 3 and 5- days post-inoculation (dpi) with two *M. oryzae* strains from RiceXPro. Treatments, Pia/Pish × P91-15B and PISH × Kyu77-07A depict incompatible (Resistant) reaction and Pish × P91-15B and ∆PISH × Kyu77-07A depict compatible (Susceptible) reaction. P91-15B and Kyu77-07A are *M. oryzae* strains while Pia, Pish and ∆PISH represent Nipponbare (NB) genotypes carrying respective genes (*Pia* and *Pish*).

Os06g0128800


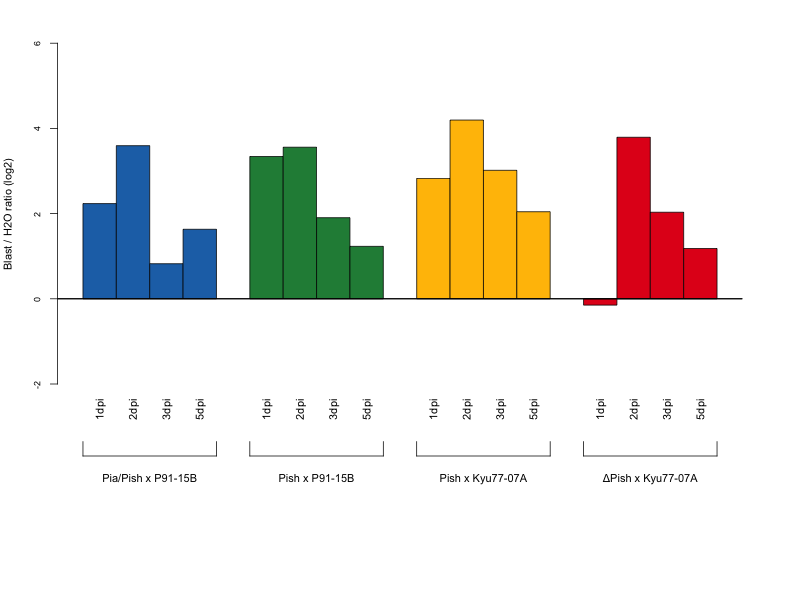
 **Fig. S6(LXXVII)**: Graphical representation of fold-change in expression of Os06g0129100 gene at 1, 2, 3 and 5- days post-inoculation (dpi) with two *M. oryzae* strains from RiceXPro. Treatments, Pia/Pish × P91-15B and PISH × Kyu77-07A depict incompatible (Resistant) reaction and Pish × P91-15B and ∆PISH × Kyu77-07A depict compatible (Susceptible) reaction. P91-15B and Kyu77-07A are *M. oryzae* strains while Pia, Pish and ∆PISH represent Nipponbare (NB) genotypes carrying respective genes (*Pia* and *Pish*).

Os06g0129100


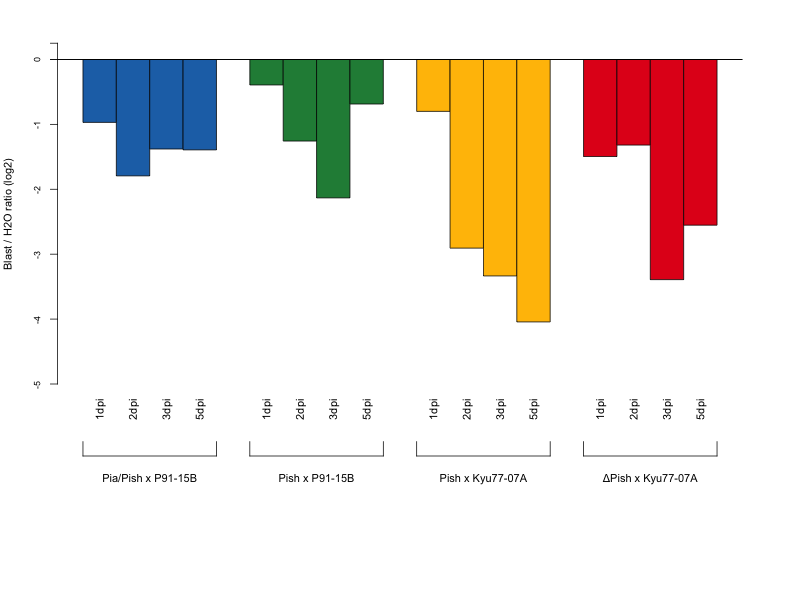
 **Fig. S6(LXXVIII)**: Graphical representation of fold-change in expression of Os06g0129600 gene at 1, 2, 3 and 5- days post-inoculation (dpi) with two *M. oryzae* strains from RiceXPro. Treatments, Pia/Pish × P91-15B and PISH × Kyu77-07A depict incompatible (Resistant) reaction and Pish × P91-15B and ∆PISH × Kyu77-07A depict compatible (Susceptible) reaction. P91-15B and Kyu77-07A are *M. oryzae* strains while Pia, Pish and ∆PISH represent Nipponbare (NB) genotypes carrying respective genes (*Pia* and *Pish*).

Os06g0129600


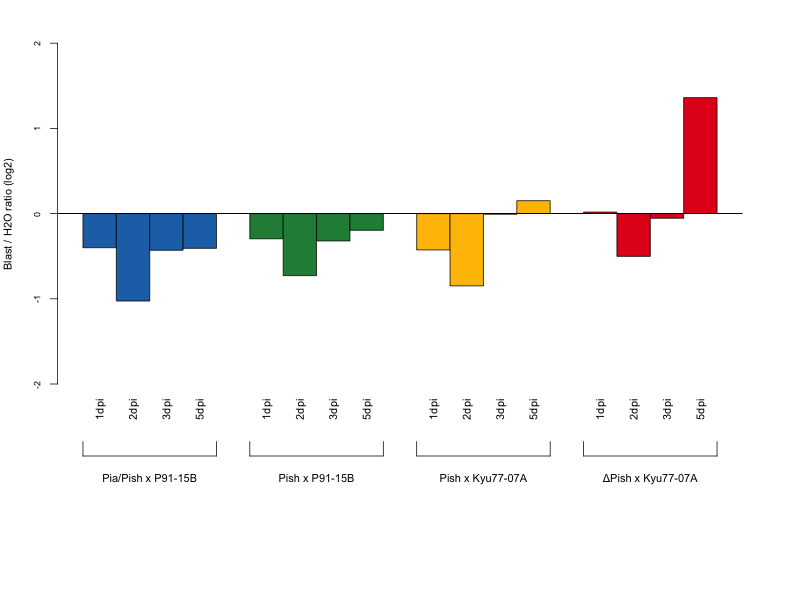
 **Fig. S6(LXXIX)**: Graphical representation of fold-change in expression of Os06g0130000 gene at 1, 2, 3 and 5- days post-inoculation (dpi) with two *M. oryzae* strains from RiceXPro. Treatments, Pia/Pish × P91-15B and PISH × Kyu77-07A depict incompatible (Resistant) reaction and Pish × P91-15B and ∆PISH × Kyu77-07A depict compatible (Susceptible) reaction. P91-15B and Kyu77-07A are *M. oryzae* strains while Pia, Pish and ∆PISH represent Nipponbare (NB) genotypes carrying respective genes (*Pia* and *Pish*).

Os06g0130000


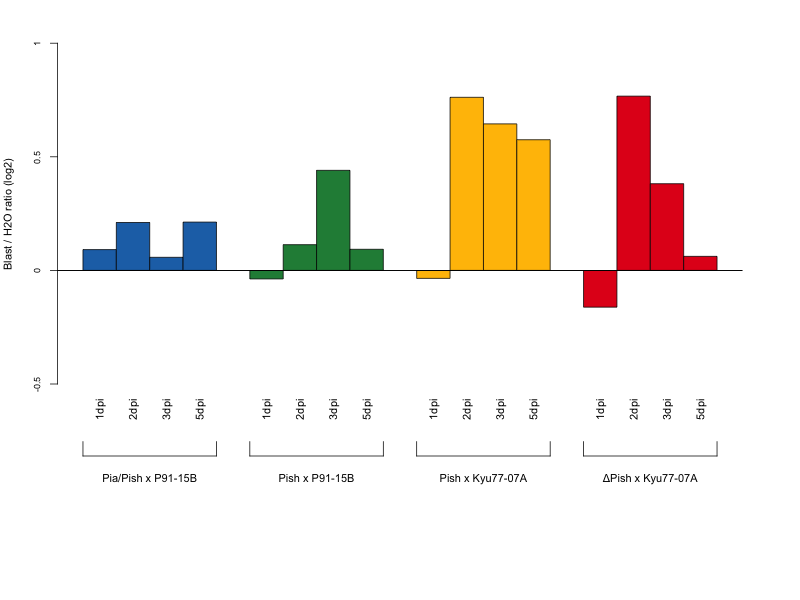
 **Fig. S6(LXXX)**: Graphical representation of fold-change in expression of Os06g0140800 gene at 1, 2, 3 and 5- days post-inoculation (dpi) with two *M. oryzae* strains from RiceXPro. Treatments, Pia/Pish × P91-15B and PISH × Kyu77-07A depict incompatible (Resistant) reaction and Pish × P91-15B and ∆PISH × Kyu77-07A depict compatible (Susceptible) reaction. P91-15B and Kyu77-07A are *M. oryzae* strains while Pia, Pish and ∆PISH represent Nipponbare (NB) genotypes carrying respective genes (*Pia* and *Pish*).

Os06g0140800


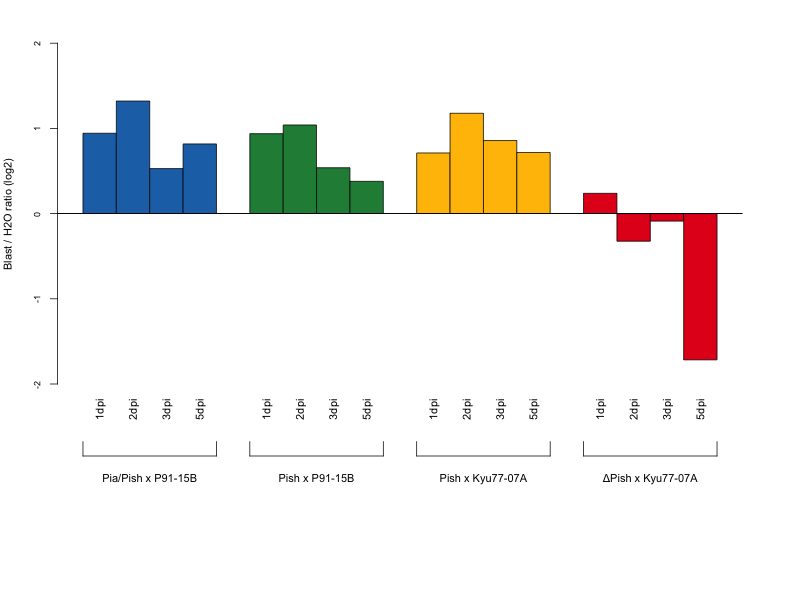
 **Fig. S6(LXXXI)**: Graphical representation of fold-change in expression of Os06g0163000 gene at 1, 2, 3 and 5- days post-inoculation (dpi) with two *M. oryzae* strains from RiceXPro. Treatments, Pia/Pish × P91-15B and PISH × Kyu77-07A depict incompatible (Resistant) reaction and Pish × P91-15B and ∆PISH × Kyu77-07A depict compatible (Susceptible) reaction. P91-15B and Kyu77-07A are *M. oryzae* strains while Pia, Pish and ∆PISH represent Nipponbare (NB) genotypes carrying respective genes (*Pia* and *Pish*).

Os06g0163000


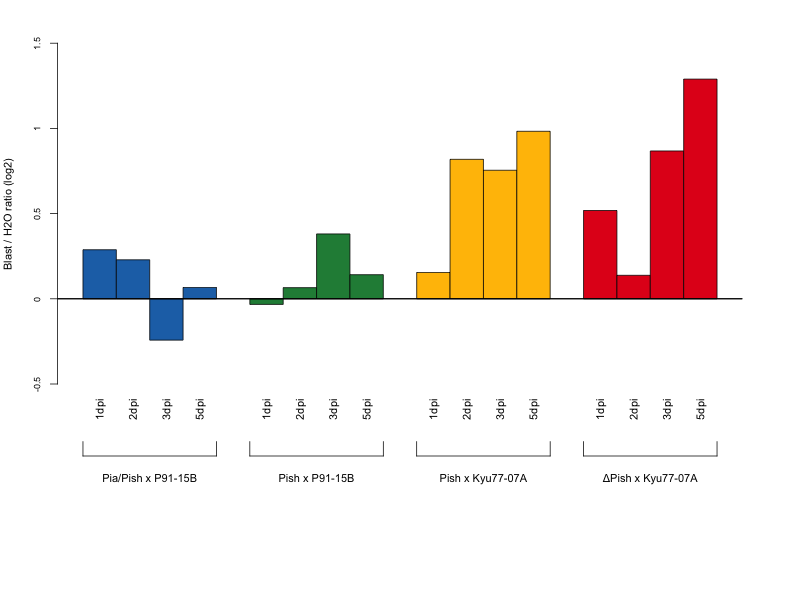
 **Fig. S6(LXXXII)**: Graphical representation of fold-change in expression of Os06g0166900 gene at 1, 2, 3 and 5- days post-inoculation (dpi) with two *M. oryzae* strains from RiceXPro. Treatments, Pia/Pish × P91-15B and PISH × Kyu77-07A depict incompatible (Resistant) reaction and Pish × P91-15B and ∆PISH × Kyu77-07A depict compatible (Susceptible) reaction. P91-15B and Kyu77-07A are *M. oryzae* strains while Pia, Pish and ∆PISH represent Nipponbare (NB) genotypes carrying respective genes (*Pia* and *Pish*).

Os06g0166900


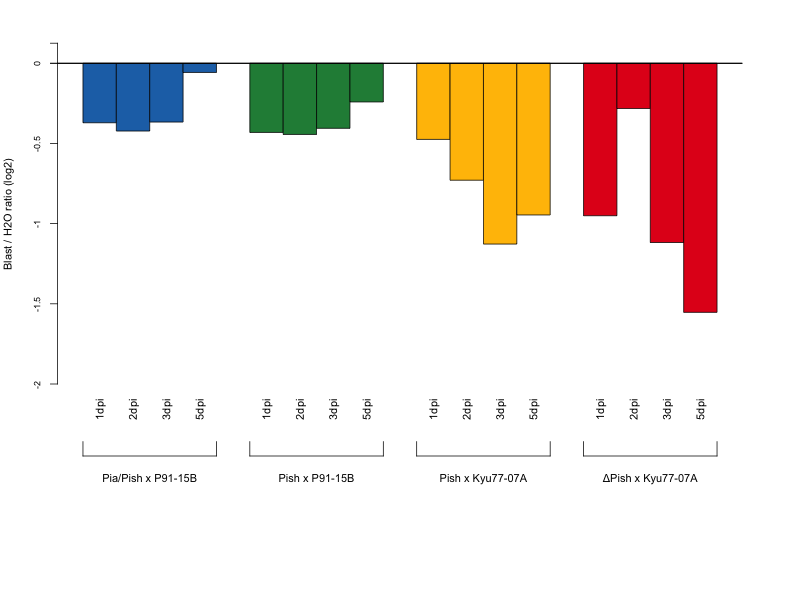
 **Fig. S6(LXXXIII)**: Graphical representation of fold-change in expression of Os06g0168800 gene at 1, 2, 3 and 5- days post-inoculation (dpi) with two *M. oryzae* strains from RiceXPro. Treatments, Pia/Pish × P91-15B and PISH × Kyu77-07A depict incompatible (Resistant) reaction and Pish × P91-15B and ∆PISH × Kyu77-07A depict compatible (Susceptible) reaction. P91-15B and Kyu77-07A are *M. oryzae* strains while Pia, Pish and ∆PISH represent Nipponbare (NB) genotypes carrying respective genes (*Pia* and *Pish*).

Os06g0168800


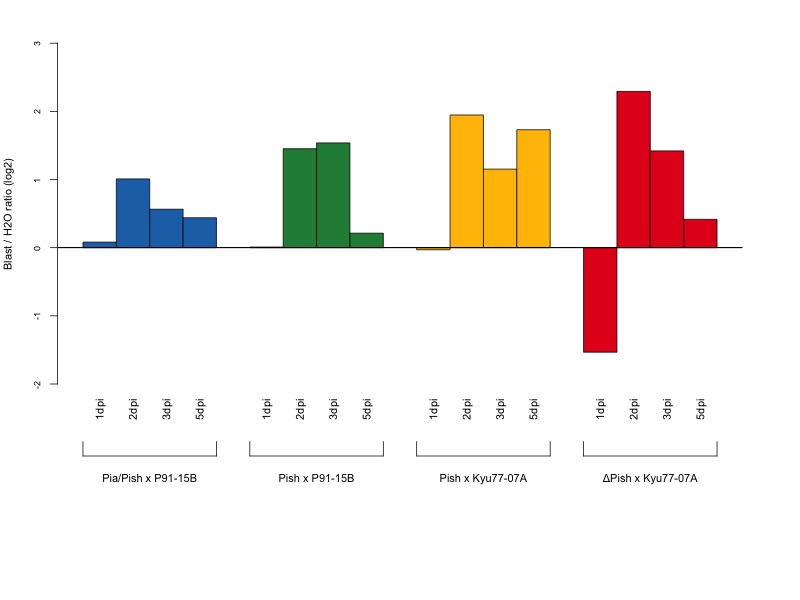
 **Fig. S6(LXXXIV)**: Graphical representation of fold-change in expression of Os06g0172800 gene at 1, 2, 3 and 5- days post-inoculation (dpi) with two *M. oryzae* strains from RiceXPro. Treatments, Pia/Pish × P91-15B and PISH × Kyu77-07A depict incompatible (Resistant) reaction and Pish × P91-15B and ∆PISH × Kyu77-07A depict compatible (Susceptible) reaction. P91-15B and Kyu77-07A are *M. oryzae* strains while Pia, Pish and ∆PISH represent Nipponbare (NB) genotypes carrying respective genes (*Pia* and *Pish*).

Os06g0172800


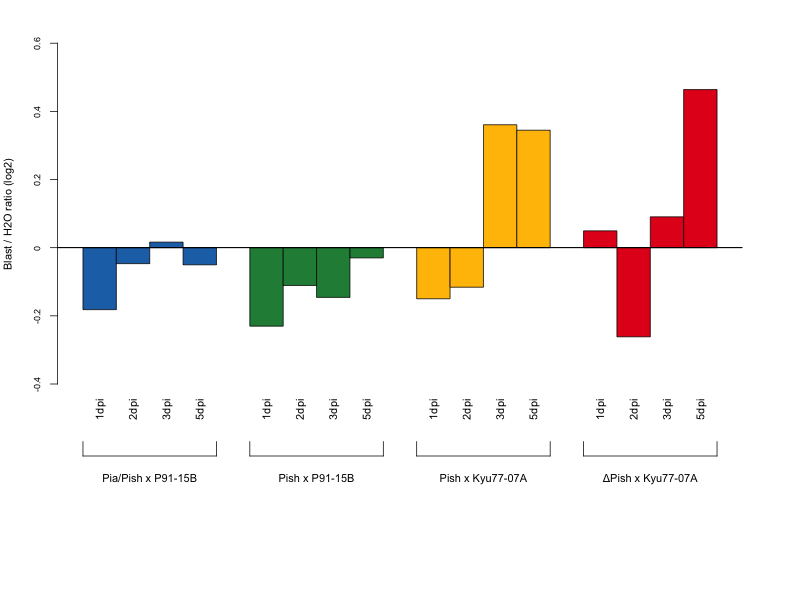
 **Fig. S6(LXXXV)**: Graphical representation of fold-change in expression of Os06g0183800 gene at 1, 2, 3 and 5- days post-inoculation (dpi) with two *M. oryzae* strains from RiceXPro. Treatments, Pia/Pish × P91-15B and PISH × Kyu77-07A depict incompatible (Resistant) reaction and Pish × P91-15B and ∆PISH × Kyu77-07A depict compatible (Susceptible) reaction. P91-15B and Kyu77-07A are *M. oryzae* strains while Pia, Pish and ∆PISH represent Nipponbare (NB) genotypes carrying respective genes (*Pia* and *Pish*).

Os06g0183800


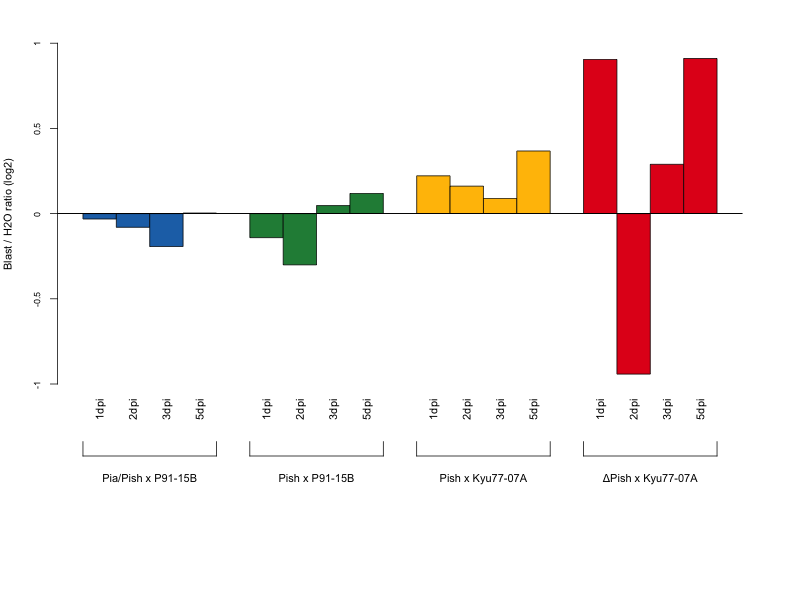
 **Fig. S6(LXXXVI)**: Graphical representation of fold-change in expression of Os06g0193400 gene at 1, 2, 3 and 5- days post-inoculation (dpi) with two *M. oryzae* strains from RiceXPro. Treatments, Pia/Pish × P91-15B and PISH × Kyu77-07A depict incompatible (Resistant) reaction and Pish × P91-15B and ∆PISH × Kyu77-07A depict compatible (Susceptible) reaction. P91-15B and Kyu77-07A are *M. oryzae* strains while Pia, Pish and ∆PISH represent Nipponbare (NB) genotypes carrying respective genes (*Pia* and *Pish*).

Os06g0193400


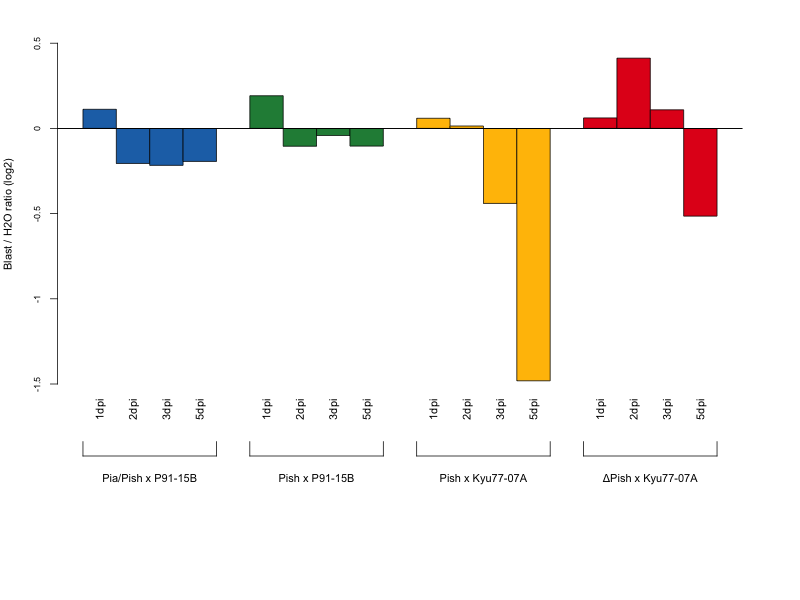
 **Fig. S6(LXXXVII)**: Graphical representation of fold-change in expression of Os06g0286500 gene at 1, 2, 3 and 5- days post-inoculation (dpi) with two *M. oryzae* strains from RiceXPro. Treatments, Pia/Pish × P91-15B and PISH × Kyu77-07A depict incompatible (Resistant) reaction and Pish × P91-15B and ∆PISH × Kyu77-07A depict compatible (Susceptible) reaction. P91-15B and Kyu77-07A are *M. oryzae* strains while Pia, Pish and ∆PISH represent Nipponbare (NB) genotypes carrying respective genes (*Pia* and *Pish*).

Os06g0286500


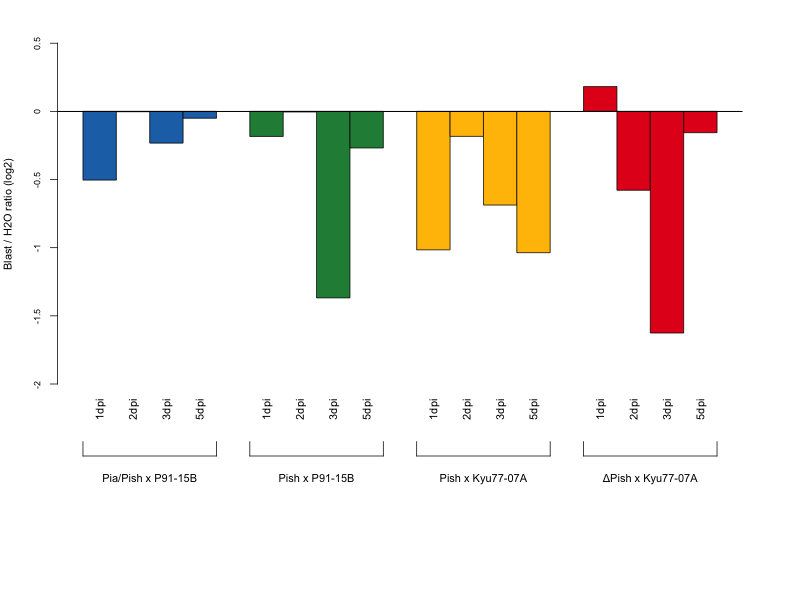
 **Fig. S6(LXXXVIII)**: Graphical representation of fold-change in expression of Os06g0286700 gene at 1, 2, 3 and 5- days post-inoculation (dpi) with two *M. oryzae* strains from RiceXPro. Treatments, Pia/Pish × P91-15B and PISH × Kyu77-07A depict incompatible (Resistant) reaction and Pish × P91-15B and ∆PISH × Kyu77-07A depict compatible (Susceptible) reaction. P91-15B and Kyu77-07A are *M. oryzae* strains while Pia, Pish and ∆PISH represent Nipponbare (NB) genotypes carrying respective genes (*Pia* and *Pish*).

Os06g0286700


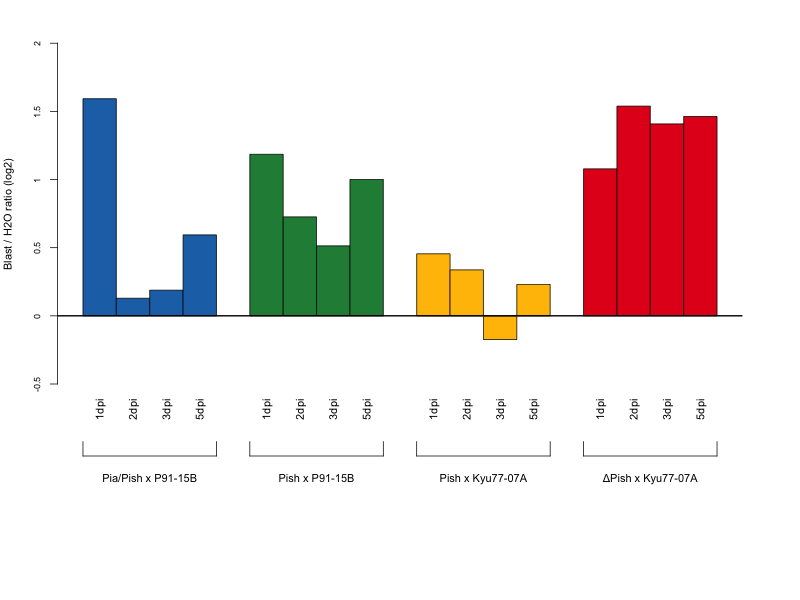
 **Fig. S6(LXXXIX)**: Graphical representation of fold-change in expression of Os06g0541600 gene at 1, 2, 3 and 5- days post-inoculation (dpi) with two *M. oryzae* strains from RiceXPro. Treatments, Pia/Pish × P91-15B and PISH × Kyu77-07A depict incompatible (Resistant) reaction and Pish × P91-15B and ∆PISH × Kyu77-07A depict compatible (Susceptible) reaction. P91-15B and Kyu77-07A are *M. oryzae* strains while Pia, Pish and ∆PISH represent Nipponbare (NB) genotypes carrying respective genes (*Pia* and *Pish*).

Os06g0541600


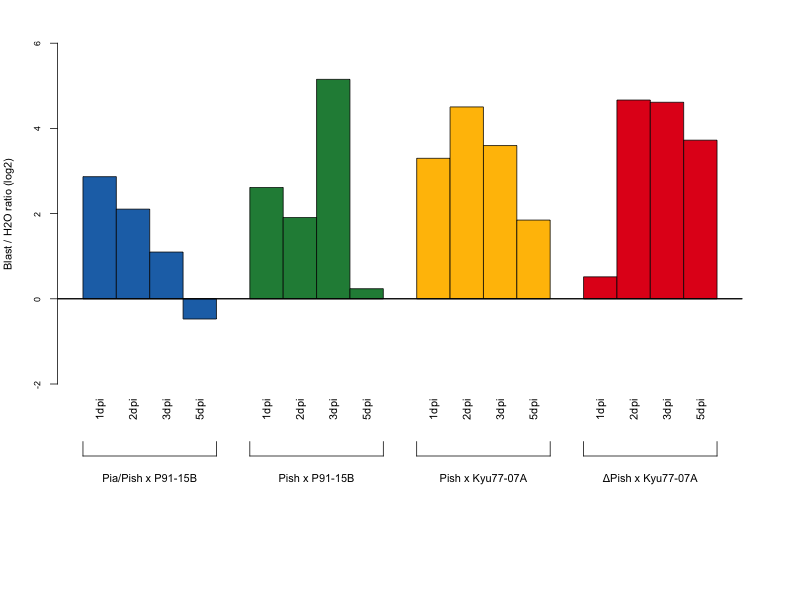
 **Fig. S6(XC)**: Graphical representation of fold-change in expression of Os06g0547400 gene at 1, 2, 3 and 5- days post-inoculation (dpi) with two *M. oryzae* strains from RiceXPro. Treatments, Pia/Pish × P91-15B and PISH × Kyu77-07A depict incompatible (Resistant) reaction and Pish × P91-15B and ∆PISH × Kyu77-07A depict compatible (Susceptible) reaction. P91-15B and Kyu77-07A are *M. oryzae* strains while Pia, Pish and ∆PISH represent Nipponbare (NB) genotypes carrying respective genes (*Pia* and *Pish*).

Os06g0547400


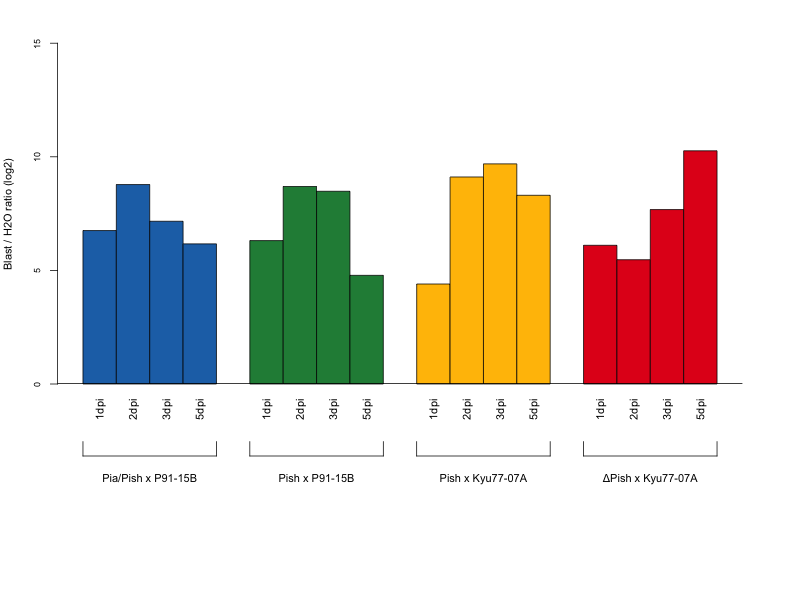
 **Fig. S6(XCI)**: Graphical representation of fold-change in expression of Os06g0569500 gene at 1, 2, 3 and 5- days post-inoculation (dpi) with two *M. oryzae* strains from RiceXPro. Treatments, Pia/Pish × P91-15B and PISH × Kyu77-07A depict incompatible (Resistant) reaction and Pish × P91-15B and ∆PISH × Kyu77-07A depict compatible (Susceptible) reaction. P91-15B and Kyu77-07A are *M. oryzae* strains while Pia, Pish and ∆PISH represent Nipponbare (NB) genotypes carrying respective genes (*Pia* and *Pish*).

Os06g0569500


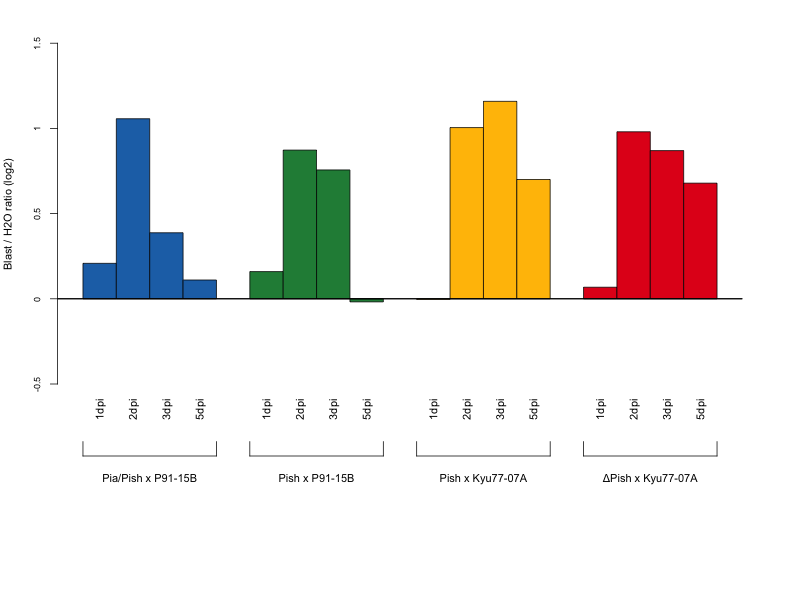
 **Fig. S6(XCII)**: Graphical representation of fold-change in expression of Os06g0605600 gene at 1, 2, 3 and 5- days post-inoculation (dpi) with two *M. oryzae* strains from RiceXPro. Treatments, Pia/Pish × P91-15B and PISH × Kyu77-07A depict incompatible (Resistant) reaction and Pish × P91-15B and ∆PISH × Kyu77-07A depict compatible (Susceptible) reaction. P91-15B and Kyu77-07A are *M. oryzae* strains while Pia, Pish and ∆PISH represent Nipponbare (NB) genotypes carrying respective genes (*Pia* and *Pish*).

Os06g0605600


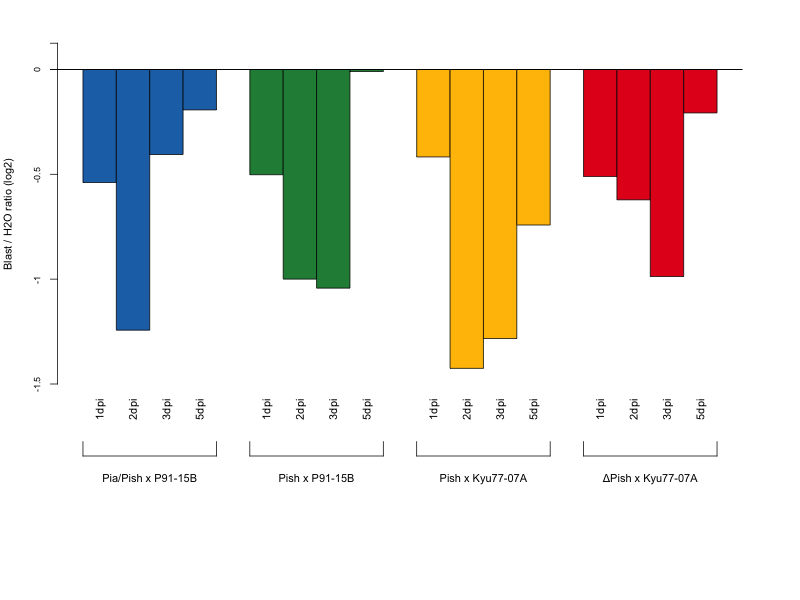
 **Fig. S6(XCIII)**: Graphical representation of fold-change in expression of Os06g0612800 gene at 1, 2, 3 and 5- days post-inoculation (dpi) with two *M. oryzae* strains from RiceXPro. Treatments, Pia/Pish × P91-15B and PISH × Kyu77-07A depict incompatible (Resistant) reaction and Pish × P91-15B and ∆PISH × Kyu77-07A depict compatible (Susceptible) reaction. P91-15B and Kyu77-07A are *M. oryzae* strains while Pia, Pish and ∆PISH represent Nipponbare (NB) genotypes carrying respective genes (*Pia* and *Pish*).

Os06g0612800


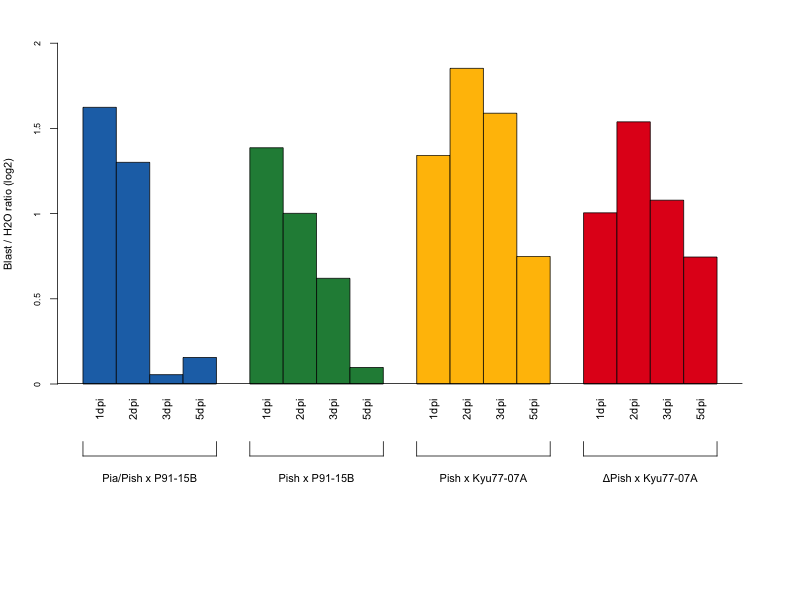


**Fig. S6(XCIV)**: Graphical representation of fold-change in expression of Os06g0622700 gene at 1, 2, 3 and 5- days post-inoculation (dpi) with two *M. oryzae* strains from RiceXPro. Treatments, Pia/Pish × P91-15B and PISH × Kyu77-07A depict incompatible (Resistant) reaction and Pish × P91-15B and ∆PISH × Kyu77-07A depict compatible (Susceptible) reaction. P91-15B and Kyu77-07A are *M. oryzae* strains while Pia, Pish and ∆PISH represent Nipponbare (NB) genotypes carrying respective genes (*Pia* and *Pish*).

Os06g0622700

**Fig. S6(XCV)**: Graphical representation of fold-change in expression of Os07g0188800 gene at 1, 2, 3 and 5- days post-inoculation (dpi) with two *M. oryzae* strains from RiceXPro. Treatments, Pia/Pish × P91-15B and PISH × Kyu77-07A depict incompatible (Resistant) reaction and Pish × P91-15B and ∆PISH × Kyu77-07A depict compatible (Susceptible) reaction. P91-15B and Kyu77-07A are *M. oryzae* strains while Pia, Pish and ∆PISH represent Nipponbare (NB) genotypes carrying respective genes (*Pia* and *Pish*).

Os07g0188800

**Fig. S6(XCVI)**: Graphical representation of fold-change in expression of Os07g0467500 gene at 1, 2, 3 and 5- days post-inoculation (dpi) with two *M. oryzae* strains from RiceXPro. Treatments, Pia/Pish × P91-15B and PISH × Kyu77-07A depict incompatible (Resistant) reaction and Pish × P91-15B and ∆PISH × Kyu77-07A depict compatible (Susceptible) reaction. P91-15B and Kyu77-07A are *M. oryzae* strains while Pia, Pish and ∆PISH represent Nipponbare (NB) genotypes carrying respective genes (*Pia* and *Pish*).

Os07g0467500

**Fig. S6(XCVII)**: Graphical representation of fold-change in expression of Os07g0481400 gene at 1, 2, 3 and 5- days post-inoculation (dpi) with two *M. oryzae* strains from RiceXPro. Treatments, Pia/Pish × P91-15B and PISH × Kyu77-07A depict incompatible (Resistant) reaction and Pish × P91-15B and ∆PISH × Kyu77-07A depict compatible (Susceptible) reaction. P91-15B and Kyu77-07A are *M. oryzae* strains while Pia, Pish and ∆PISH represent Nipponbare (NB) genotypes carrying respective genes (*Pia* and *Pish*).

Os07g0481400

**Fig. S6(XCVIII)**: Graphical representation of fold-change in expression of Os07g0658300 gene at 1, 2, 3 and 5- days post-inoculation (dpi) with two *M. oryzae* strains from RiceXPro. Treatments, Pia/Pish × P91-15B and PISH × Kyu77-07A depict incompatible (Resistant) reaction and Pish × P91-15B and ∆PISH × Kyu77-07A depict compatible (Susceptible) reaction. P91-15B and Kyu77-07A are *M. oryzae* strains while Pia, Pish and ∆PISH represent Nipponbare (NB) genotypes carrying respective genes (*Pia* and *Pish*).

**Fig. S6(XCIX)**: Graphical representation of fold-change in expression of Os07g0660200 gene at 1, 2, 3 and 5- days post-inoculation (dpi) with two *M. oryzae* strains from RiceXPro. Treatments, Pia/Pish × P91-15B and PISH × Kyu77-07A depict incompatible (Resistant) reaction and Pish × P91-15B and ∆PISH × Kyu77-07A depict compatible (Susceptible) reaction. P91-15B and Kyu77-07A are *M. oryzae* strains while Pia, Pish and ∆PISH represent Nipponbare (NB) genotypes carrying respective genes (*Pia* and *Pish*).

**Fig. S6(C)**: Graphical representation of fold-change in expression of Os11g0194900 gene at 1, 2, 3 and 5- days post-inoculation (dpi) with two *M. oryzae* strains from RiceXPro. Treatments, Pia/Pish × P91-15B and PISH × Kyu77-07A depict incompatible (Resistant) reaction and Pish × P91-15B and ∆PISH × Kyu77-07A depict compatible (Susceptible) reaction. P91-15B and Kyu77-07A are *M. oryzae* strains while Pia, Pish and ∆PISH represent Nipponbare (NB) genotypes carrying respective genes (*Pia* and *Pish*).

**Fig. S6(CI)**: Graphical representation of fold-change in expression of Os11g0206700 gene at 1, 2, 3 and 5- days post-inoculation (dpi) with two *M. oryzae* strains from RiceXPro. Treatments, Pia/Pish × P91-15B and PISH × Kyu77-07A depict incompatible (Resistant) reaction and Pish × P91-15B and ∆PISH × Kyu77-07A depict compatible (Susceptible) reaction. P91-15B and Kyu77-07A are *M. oryzae* strains while Pia, Pish and ∆PISH represent Nipponbare (NB) genotypes carrying respective genes (*Pia* and *Pish*).

**Fig. S6(CII)**: Graphical representation of fold-change in expression of Os11g0213500 gene at 1, 2, 3 and 5- days post-inoculation (dpi) with two *M. oryzae* strains from RiceXPro. Treatments, Pia/Pish × P91-15B and PISH × Kyu77-07A depict incompatible (Resistant) reaction and Pish × P91-15B and ∆PISH × Kyu77-07A depict compatible (Susceptible) reaction. P91-15B and Kyu77-07A are *M. oryzae* strains while Pia, Pish and ∆PISH represent Nipponbare (NB) genotypes carrying respective genes (*Pia* and *Pish*).

**Fig. S6(CIII)**: Graphical representation of fold-change in expression of Os11g0224900 gene at 1, 2, 3 and 5- days post-inoculation (dpi) with two *M. oryzae* strains from RiceXPro. Treatments, Pia/Pish × P91-15B and PISH × Kyu77-07A depict incompatible (Resistant) reaction and Pish × P91-15B and ∆PISH × Kyu77-07A depict compatible (Susceptible) reaction. P91-15B and Kyu77-07A are *M. oryzae* strains while Pia, Pish and ∆PISH represent Nipponbare (NB) genotypes carrying respective genes (*Pia* and *Pish*).

**Fig. S6(CIV)**: Graphical representation of fold-change in expression of Os11g0225100 gene at 1, 2, 3 and 5- days post-inoculation (dpi) with two *M. oryzae* strains from RiceXPro. Treatments, Pia/Pish × P91-15B and PISH × Kyu77-07A depict incompatible (Resistant) reaction and Pish × P91-15B and ∆PISH × Kyu77-07A depict compatible (Susceptible) reaction. P91-15B and Kyu77-07A are *M. oryzae* strains while Pia, Pish and ∆PISH represent Nipponbare (NB) genotypes carrying respective genes (*Pia* and *Pish*).

**Fig. S6(CV)**: Graphical representation of fold-change in expression of Os11g0225300 gene at 1, 2, 3 and 5- days post-inoculation (dpi) with two *M. oryzae* strains from RiceXPro. Treatments, Pia/Pish × P91-15B and PISH × Kyu77-07A depict incompatible (Resistant) reaction and Pish × P91-15B and ∆PISH × Kyu77-07A depict compatible (Susceptible) reaction. P91-15B and Kyu77-07A are *M. oryzae* strains while Pia, Pish and ∆PISH represent Nipponbare (NB) genotypes carrying respective genes (*Pia* and *Pish*).

**Fig. S6(CVI)**: Graphical representation of fold-change in expression of Os11g0225300 gene at 1, 2, 3 and 5- days post-inoculation (dpi) with two *M. oryzae* strains from RiceXPro. Treatments, Pia/Pish × P91-15B and PISH × Kyu77-07A depict incompatible (Resistant) reaction and Pish × P91-15B and ∆PISH × Kyu77-07A depict compatible (Susceptible) reaction. P91-15B and Kyu77-07A are *M. oryzae* strains while Pia, Pish and ∆PISH represent Nipponbare (NB) genotypes carrying respective genes (*Pia* and *Pish*).

**Fig. S6(CVII)**: Graphical representation of fold-change in expression of Os11g0264200 gene at 1, 2, 3 and 5- days post-inoculation (dpi) with two *M. oryzae* strains from RiceXPro. Treatments, Pia/Pish × P91-15B and PISH × Kyu77-07A depict incompatible (Resistant) reaction and Pish × P91-15B and ∆PISH × Kyu77-07A depict compatible (Susceptible) reaction. P91-15B and Kyu77-07A are *M. oryzae* strains while Pia, Pish and ∆PISH represent Nipponbare (NB) genotypes carrying respective genes (*Pia* and *Pish*).

**Fig. S6(CVIII)**: Graphical representation of fold-change in expression of Os11g0264200 gene at 1, 2, 3 and 5- days post-inoculation (dpi) with two *M. oryzae* strains from RiceXPro. Treatments, Pia/Pish × P91-15B and PISH × Kyu77-07A depict incompatible (Resistant) reaction and Pish × P91-15B and ∆PISH × Kyu77-07A depict compatible (Susceptible) reaction. P91-15B and Kyu77-07A are *M. oryzae* strains while Pia, Pish and ∆PISH represent Nipponbare (NB) genotypes carrying respective genes (*Pia* and *Pish*).

**Fig. S6(CIX)**: Graphical representation of fold-change in expression of Os11g0264200 gene at 1, 2, 3 and 5- days post-inoculation (dpi) with two *M. oryzae* strains from RiceXPro. Treatments, Pia/Pish × P91-15B and PISH × Kyu77-07A depict incompatible (Resistant) reaction and Pish × P91-15B and ∆PISH × Kyu77-07A depict compatible (Susceptible) reaction. P91-15B and Kyu77-07A are *M. oryzae* strains while Pia, Pish and ∆PISH represent Nipponbare (NB) genotypes carrying respective genes (*Pia* and *Pish*).

**Fig. S6(CX)**: Graphical representation of fold-change in expression of Os11g0588600 gene at 1, 2, 3 and 5- days post-inoculation (dpi) with two *M. oryzae* strains from RiceXPro. Treatments, Pia/Pish × P91-15B and PISH × Kyu77-07A depict incompatible (Resistant) reaction and Pish × P91-15B and ∆PISH × Kyu77-07A depict compatible (Susceptible) reaction. P91-15B and Kyu77-07A are *M. oryzae* strains while Pia, Pish and ∆PISH represent Nipponbare (NB) genotypes carrying respective genes (*Pia* and *Pish*).

**Fig. S6(CXI)**: Graphical representation of fold-change in expression of Os11g0588600 gene at 1, 2, 3 and 5- days post-inoculation (dpi) with two *M. oryzae* strains from RiceXPro. Treatments, Pia/Pish × P91-15B and PISH × Kyu77-07A depict incompatible (Resistant) reaction and Pish × P91-15B and ∆PISH × Kyu77-07A depict compatible (Susceptible) reaction. P91-15B and Kyu77-07A are *M. oryzae* strains while Pia, Pish and ∆PISH represent Nipponbare (NB) genotypes carrying respective genes (*Pia* and *Pish*).

**Fig. S6(CXII)**: Graphical representation of fold-change in expression of Os11g0590700 gene at 1, 2, 3 and 5- days post-inoculation (dpi) with two *M. oryzae* strains from RiceXPro. Treatments, Pia/Pish × P91-15B and PISH × Kyu77-07A depict incompatible (Resistant) reaction and Pish × P91-15B and ∆PISH × Kyu77-07A depict compatible (Susceptible) reaction. P91-15B and Kyu77-07A are *M. oryzae* strains while Pia, Pish and ∆PISH represent Nipponbare (NB) genotypes carrying respective genes (*Pia* and *Pish*).

**Fig. S6(CXIII)**: Graphical representation of fold-change in expression of Os11g0598500 gene at 1, 2, 3 and 5- days post-inoculation (dpi) with two *M. oryzae* strains from RiceXPro. Treatments, Pia/Pish × P91-15B and PISH × Kyu77-07A depict incompatible (Resistant) reaction and Pish × P91-15B and ∆PISH × Kyu77-07A depict compatible (Susceptible) reaction. P91-15B and Kyu77-07A are *M. oryzae* strains while Pia, Pish and ∆PISH represent Nipponbare (NB) genotypes carrying respective genes (*Pia* and *Pish*).

**Fig. S6(CXIV)**: Graphical representation of fold-change in expression of Os11g0598500 gene at 1, 2, 3 and 5- days post-inoculation (dpi) with two *M. oryzae* strains from RiceXPro. Treatments, Pia/Pish × P91-15B and PISH × Kyu77-07A depict incompatible (Resistant) reaction and Pish × P91-15B and ∆PISH × Kyu77-07A depict compatible (Susceptible) reaction. P91-15B and Kyu77-07A are *M. oryzae* strains while Pia, Pish and ∆PISH represent Nipponbare (NB) genotypes carrying respective genes (*Pia* and *Pish*).

**Fig. S6(CXV)**: Graphical representation of fold-change in expression of Os11g0598500 gene at 1, 2, 3 and 5- days post-inoculation (dpi) with two *M. oryzae* strains from RiceXPro. Treatments, Pia/Pish × P91-15B and PISH × Kyu77-07A depict incompatible (Resistant) reaction and Pish × P91-15B and ∆PISH × Kyu77-07A depict compatible (Susceptible) reaction. P91-15B and Kyu77-07A are *M. oryzae* strains while Pia, Pish and ∆PISH represent Nipponbare (NB) genotypes carrying respective genes (*Pia* and *Pish*).

**Fig. S6(CXVI)**: Graphical representation of fold-change in expression of Os11g0621300 gene at 1, 2, 3 and 5- days post-inoculation (dpi) with two *M. oryzae* strains from RiceXPro. Treatments, Pia/Pish × P91-15B and PISH × Kyu77-07A depict incompatible (Resistant) reaction and Pish × P91-15B and ∆PISH × Kyu77-07A depict compatible (Susceptible) reaction. P91-15B and Kyu77-07A are *M. oryzae* strains while Pia, Pish and ∆PISH represent Nipponbare (NB) genotypes carrying respective genes (*Pia* and *Pish*).

**Fig. S6(CXVII)**: Graphical representation of fold-change in expression of Os11g0633500 gene at 1, 2, 3 and 5- days post-inoculation (dpi) with two *M. oryzae* strains from RiceXPro. Treatments, Pia/Pish × P91-15B and PISH × Kyu77-07A depict incompatible (Resistant) reaction and Pish × P91-15B and ∆PISH × Kyu77-07A depict compatible (Susceptible) reaction. P91-15B and Kyu77-07A are *M. oryzae* strains while Pia, Pish and ∆PISH represent Nipponbare (NB) genotypes carrying respective genes (*Pia* and *Pish*).

**Fig. S6(CXVIII)**: Graphical representation of fold-change in expression of Os11g0639100 gene at 1, 2, 3 and 5- days post-inoculation (dpi) with two *M. oryzae* strains from RiceXPro. Treatments, Pia/Pish × P91-15B and PISH × Kyu77-07A depict incompatible (Resistant) reaction and Pish × P91-15B and ∆PISH × Kyu77-07A depict compatible (Susceptible) reaction. P91-15B and Kyu77-07A are *M. oryzae* strains while Pia, Pish and ∆PISH represent Nipponbare (NB) genotypes carrying respective genes (*Pia* and *Pish*).

**Fig. S6(CXIX)**: Graphical representation of fold-change in expression of Os11g0673600 gene at 1, 2, 3 and 5- days post-inoculation (dpi) with two *M. oryzae* strains from RiceXPro. Treatments, Pia/Pish × P91-15B and PISH × Kyu77-07A depict incompatible (Resistant) reaction and Pish × P91-15B and ∆PISH × Kyu77-07A depict compatible (Susceptible) reaction. P91-15B and Kyu77-07A are *M. oryzae* strains while Pia, Pish and ∆PISH represent Nipponbare (NB) genotypes carrying respective genes (*Pia* and *Pish*).

**Fig. S6(CXX)**: Graphical representation of fold-change in expression of Os11g0673900 gene at 1, 2, 3 and 5- days post-inoculation (dpi) with two *M. oryzae* strains from RiceXPro. Treatments, Pia/Pish × P91-15B and PISH × Kyu77-07A depict incompatible (Resistant) reaction and Pish × P91-15B and ∆PISH × Kyu77-07A depict compatible (Susceptible) reaction. P91-15B and Kyu77-07A are *M. oryzae* strains while Pia, Pish and ∆PISH represent Nipponbare (NB) genotypes carrying respective genes (*Pia* and *Pish*).

**Fig. S6(CXXI)**: Graphical representation of fold-change in expression of Os11g0677400 gene at 1, 2, 3 and 5- days post-inoculation (dpi) with two *M. oryzae* strains from RiceXPro. Treatments, Pia/Pish × P91-15B and PISH × Kyu77-07A depict incompatible (Resistant) reaction and Pish × P91-15B and ∆PISH × Kyu77-07A depict compatible (Susceptible) reaction. P91-15B and Kyu77-07A are *M. oryzae* strains while Pia, Pish and ∆PISH represent Nipponbare (NB) genotypes carrying respective genes (*Pia* and *Pish*).

**Fig. S6(CXXII)**: Graphical representation of fold-change in expression of Os11g0681400 gene at 1, 2, 3 and 5- days post-inoculation (dpi) with two *M. oryzae* strains from RiceXPro. Treatments, Pia/Pish × P91-15B and PISH × Kyu77-07A depict incompatible (Resistant) reaction and Pish × P91-15B and ∆PISH × Kyu77-07A depict compatible (Susceptible) reaction. P91-15B and Kyu77-07A are *M. oryzae* strains while Pia, Pish and ∆PISH represent Nipponbare (NB) genotypes carrying respective genes (*Pia* and *Pish*).

**Fig. S6(CXXIII)**: Graphical representation of fold-change in expression of Os11g0682600 gene at 1, 2, 3 and 5- days post-inoculation (dpi) with two *M. oryzae* strains from RiceXPro. Treatments, Pia/Pish × P91-15B and PISH × Kyu77-07A depict incompatible (Resistant) reaction and Pish × P91-15B and ∆PISH × Kyu77-07A depict compatible (Susceptible) reaction. P91-15B and Kyu77-07A are *M. oryzae* strains while Pia, Pish and ∆PISH represent Nipponbare (NB) genotypes carrying respective genes (*Pia* and *Pish*).

**Fig. S6(CXXIV)**: Graphical representation of fold-change in expression of Os11g0683500 gene at 1, 2, 3 and 5- days post-inoculation (dpi) with two *M. oryzae* strains from RiceXPro. Treatments, Pia/Pish × P91-15B and PISH × Kyu77-07A depict incompatible (Resistant) reaction and Pish × P91-15B and ∆PISH × Kyu77-07A depict compatible (Susceptible) reaction. P91-15B and Kyu77-07A are *M. oryzae* strains while Pia, Pish and ∆PISH represent Nipponbare (NB) genotypes carrying respective genes (*Pia* and *Pish*).

**Fig. S6(CXXV)**: Graphical representation of fold-change in expression of Os11g0683500 gene at 1, 2, 3 and 5- days post-inoculation (dpi) with two *M. oryzae* strains from RiceXPro. Treatments, Pia/Pish × P91-15B and PISH × Kyu77-07A depict incompatible (Resistant) reaction and Pish × P91-15B and ∆PISH × Kyu77-07A depict compatible (Susceptible) reaction. P91-15B and Kyu77-07A are *M. oryzae* strains while Pia, Pish and ∆PISH represent Nipponbare (NB) genotypes carrying respective genes (*Pia* and *Pish*).

**Fig. S6(CXXVI)**: Graphical representation of fold-change in expression of Os11g0683500 gene at 1, 2, 3 and 5- days post-inoculation (dpi) with two *M. oryzae* strains from RiceXPro. Treatments, Pia/Pish × P91-15B and PISH × Kyu77-07A depict incompatible (Resistant) reaction and Pish × P91-15B and ∆PISH × Kyu77-07A depict compatible (Susceptible) reaction. P91-15B and Kyu77-07A are *M. oryzae* strains while Pia, Pish and ∆PISH represent Nipponbare (NB) genotypes carrying respective genes (*Pia* and *Pish*).

**Fig. S6(CXXVII)**: Graphical representation of fold-change in expression of RXP_3001-Os11g0683800 gene at 1, 2, 3 and 5- days post-inoculation (dpi) with two *M. oryzae* strains from RiceXPro. Treatments, Pia/Pish × P91-15B and PISH × Kyu77-07A depict incompatible (Resistant) reaction and Pish × P91-15B and ∆PISH × Kyu77-07A depict compatible (Susceptible) reaction. P91-15B and Kyu77-07A are *M. oryzae* strains while Pia, Pish and ∆PISH represent Nipponbare (NB) genotypes carrying respective genes (*Pia* and *Pish*).

**Fig. S6(CXXVIII)**: Graphical representation of fold-change in expression of RXP_3001-Os11g0683800 gene at 1, 2, 3 and 5- days post-inoculation (dpi) with two *M. oryzae* strains from RiceXPro. Treatments, Pia/Pish × P91-15B and PISH × Kyu77-07A depict incompatible (Resistant) reaction and Pish × P91-15B and ∆PISH × Kyu77-07A depict compatible (Susceptible) reaction. P91-15B and Kyu77-07A are *M. oryzae* strains while Pia, Pish and ∆PISH represent Nipponbare (NB) genotypes carrying respective genes (*Pia* and *Pish*).

**Fig. S6(CXXIX)**: Graphical representation of fold-change in expression of RXP_3001- Os11g0684100 gene at 1, 2, 3 and 5- days post-inoculation (dpi) with two *M. oryzae* strains from RiceXPro. Treatments, Pia/Pish × P91-15B and PISH × Kyu77-07A depict incompatible (Resistant) reaction and Pish × P91-15B and ∆PISH × Kyu77-07A depict compatible (Susceptible) reaction. P91-15B and Kyu77-07A are *M. oryzae* strains while Pia, Pish and ∆PISH represent Nipponbare (NB) genotypes carrying respective genes (*Pia* and *Pish*).

**Fig. S6(CXXX)**: Graphical representation of fold-change in expression of RXP_3001- Os11g0684100 gene at 1, 2, 3 and 5- days post-inoculation (dpi) with two *M. oryzae* strains from RiceXPro. Treatments, Pia/Pish × P91-15B and PISH × Kyu77-07A depict incompatible (Resistant) reaction and Pish × P91-15B and ∆PISH × Kyu77-07A depict compatible (Susceptible) reaction. P91-15B and Kyu77-07A are *M. oryzae* strains while Pia, Pish and ∆PISH represent Nipponbare (NB) genotypes carrying respective genes (*Pia* and *Pish*).

**Fig. S6(CXXXI)**: Graphical representation of fold-change in expression of RXP_3001- Os11g0689100 gene at 1, 2, 3 and 5- days post-inoculation (dpi) with two *M. oryzae* strains from RiceXPro. Treatments, Pia/Pish × P91-15B and PISH × Kyu77-07A depict incompatible (Resistant) reaction and Pish × P91-15B and ∆PISH × Kyu77-07A depict compatible (Susceptible) reaction. P91-15B and Kyu77-07A are *M. oryzae* strains while Pia, Pish and ∆PISH represent Nipponbare (NB) genotypes carrying respective genes (*Pia* and *Pish*).

**Fig. S6(CXXXII)**: Graphical representation of fold-change in expression of RXP_3001- Os11g0689100 gene at 1, 2, 3 and 5- days post-inoculation (dpi) with two *M. oryzae* strains from RiceXPro. Treatments, Pia/Pish × P91-15B and PISH × Kyu77-07A depict incompatible (Resistant) reaction and Pish × P91-15B and ∆PISH × Kyu77-07A depict compatible (Susceptible) reaction. P91-15B and Kyu77-07A are *M. oryzae* strains while Pia, Pish and ∆PISH represent Nipponbare (NB) genotypes carrying respective genes (*Pia* and *Pish*).
